# C–C Bond Cleavage in the Late-Stage Biosynthesis of Huperzine Alkaloids Occurs via Enzymatic Retro-Aza-Prins Reaction

**DOI:** 10.1021/jacs.4c10410

**Published:** 2025-05-09

**Authors:** Stefan E. Payer, Mario Prejanò, Philipp Kögl, Tamara Reiter, Eva-Maria Pferschy-Wenzig, Fahmi Himo, Wolfgang Kroutil

**Affiliations:** † Institute of Chemistry, University of Graz, BioTechMed Graz, Heinrichstrasse 28, A-8010 Graz, Austria; ‡ Enzyan Biocatalysis GmbH, Stiftingtalstraße 14, A-8010 Graz, Austria; § Department of Organic Chemistry, Arrhenius Laboratory, Stockholm University, SE-106 91 Stockholm, Sweden; ∥ Dipartimento di Chimica e Tecnologie Chimiche, Università della Calabria, Via P. Bucci, 87036 Rende, Italy; ⊥ Institute of Pharmaceutical Sciences, Pharmacognosy, University of Graz, Beethovenstrasse 8, A-8010 Graz, Austria; # Field of Excellence BioHealth, University of Graz, 8010 Graz, Austria

## Abstract

The demand for novel enzyme-catalyzed reactions in chemical synthesis has spurred the development of many new-to-nature reactions. Additionally, detailed analysis of biosynthetic pathways can uncover unprecedented chemical/enzymatic mechanisms. In this study, we revisited the catalytic mechanism of the 2-oxoglutarate-dependent dioxygenase Pt2OGD-1, involved in the biosynthesis of huperzine alkaloids. Our experimental and computational investigations uncovered a previously unknown enzymatic C–C bond cleavage in the piperidine ring of the alkaloid scaffold, resembling an oxidative retro-aza-Prins reaction. Here, this transformation is initiated by hydrogen abstraction, followed by electron transfer at the 4-position of the heterocycle, triggering ring opening and finally resulting in the loss of a carbon atom as formaldehyde. This discovery expands the toolbox of reactions, enhances our understanding of these enzymes, and may facilitate their application in the biotechnological production of pharmaceutically relevant alkaloid scaffolds as well as the development of biocatalysts with similar activities.

## Graphical abstract

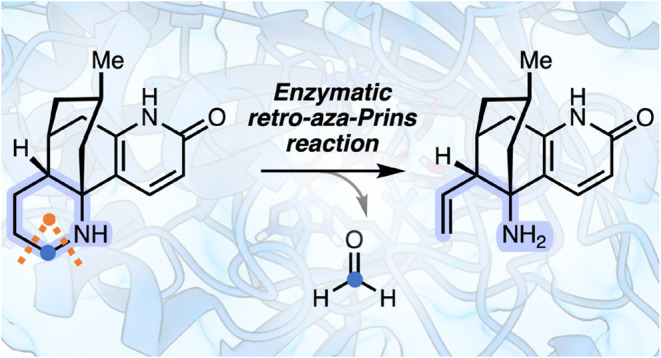

## Introduction

Biosynthetic pathways toward secondary metabolites are a rich source for novel biocatalysts^1–4^ that have been exploited in both industry and academia for the synthesis of pharmaceuticals, agrochemicals, and fragrance compounds.^5–9^ Consequently, many established named reactions from organic chemistry are mirrored in biological synthetic routes.^10,11^ Interestingly, not all named reactions have a known natural analogue, prompting the design of new-to-nature reactions.^12^ However, as many biosynthetic pathways remain unexplored, it is also plausible that novel enzymatic reactions analogous to a named reaction will be discovered. Here, we illustrate this for a retro-aza-Prins reaction.

A combined metabolomics/transcriptomics approach has recently led to the discovery of a set of enzymes involved in the late-stage biosynthesis of the potent acetylcholinesterase (AChE)-inhibitor huperzine A (**6**) in different parts of the Lycopodiaceae plant Huperzia tetrasticha (synonym: Phlegmariurus tetrastichus) (Scheme 1a).^13,14^ Polyketide-synthase-like enzymes and neofunctional carbonic anhydrases are involved in the initial scaffold assembly of compound **1** from l-lysine and acetyl-CoA. Yet unknown enzymes facilitate the cyclization and conversion of **1** to the tetracyclic precursor flabellidine (**2**). Finally, several 2-oxoglutarate-dependent dioxygenases (2OGDs) were found to be responsible for the late-stage oxidative tailoring and diversification of the alkaloid scaffold. Among the latter, 2OGD-1 is particularly intriguing since it catalyzes the oxidative excision of C9 from ring C in huperzine B (**4**) along with the installation of the characteristic terminal olefin in huperzine C (**5**), a direct precursor of **6**.

**Scheme 1:**
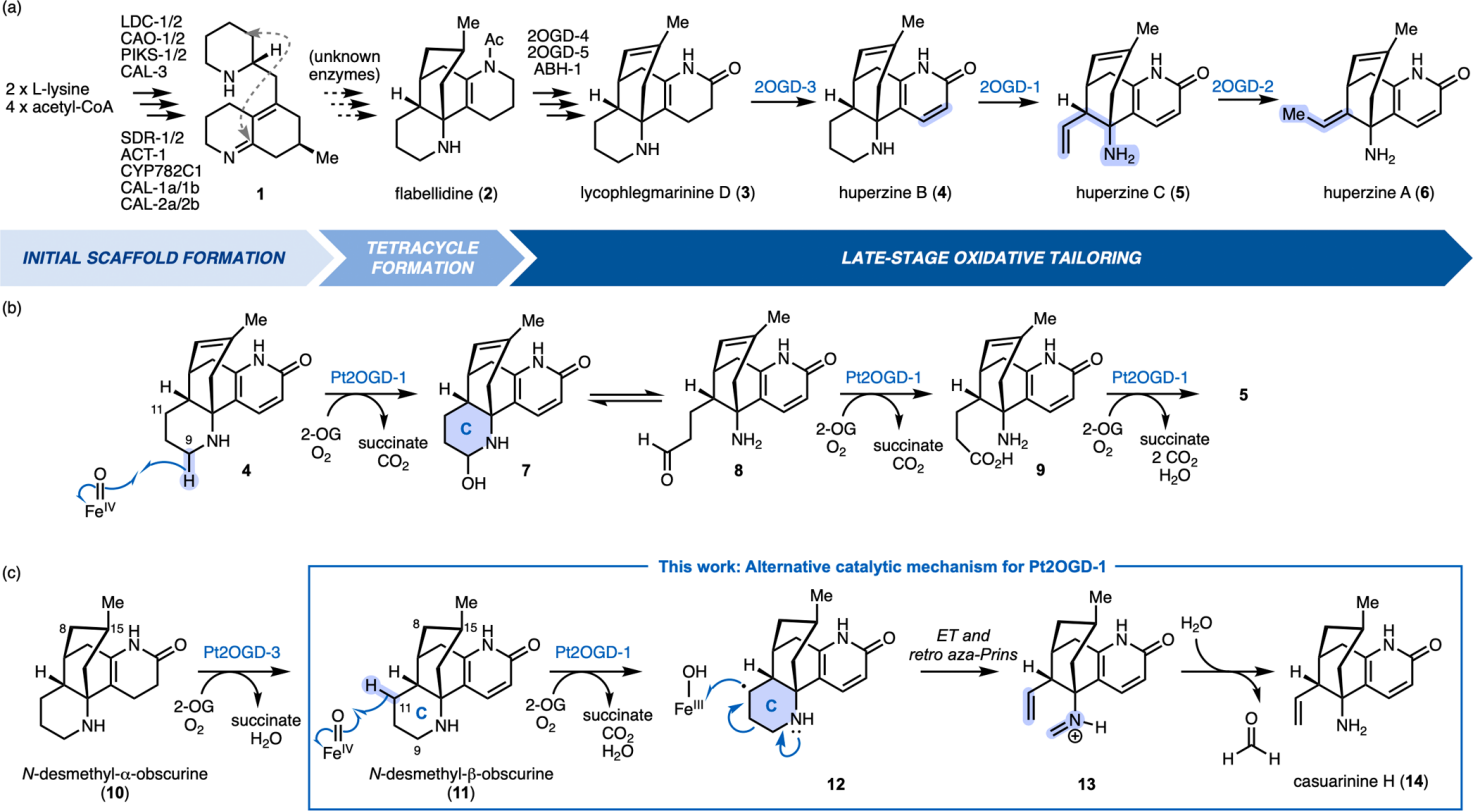
Biosynthesis of Huperzine A and Mechanistic Proposals for the Late-Stage Oxidative Modification Catalyzed by Pt2OGD-1^a^

a (a) The biosynthesis of the neuroactive AChE inhibitor huperzine A is proposed to proceed in three stages which include the following enzymes: LDC: lysine-6 decarboxylase; CAO: copper amine oxidase; PIKS: piperidyl ketide synthase; CAL: a-carbonic anhydrase-like enzyme, SDR: short-chain dehydrogenase/reductase; ACT: acyltransferase; CYP782C1: cytochrome P450 dioxygenase; 2OGD: 2-oxoglutarate dependent dioxygenase; ABH: α/β hydrolase family enzyme; uncharacterized steps are indicated by dashed arrows. (b) Previously, three consecutive oxidation events catalyzed by the same enzyme to open ring C via C–N bond cleavage and convert **4** into **5** were proposed. (c) Alternatively, the same transformation could be achieved with a single oxidative activation and ring C opening via C–C bond cleavage. Pt2OGD-3 and Pt2OGD-1 were described to convert the Δ8,15 unsaturated compounds **3** and **4**, but also act on the saturated congeners **10** and **11**, respectively.

2OGDs are nonheme iron-containing enzymes that activate molecular oxygen through decarboxylation of the cosubstrate 2-oxoglutarate (2-OG). The resulting iron­(IV)-oxo (“ferryl”) species abstracts a hydrogen atom from a substrate and generates an open-shell radical species that can further react via manifold pathways. Usually, a rebound event to the iron­(III)-hydroxyl species leads to the hydroxylated substrate derivative. However, by stabilizing certain substrate conformations and controlling the access of cosubstrates, e.g., halides, the enzyme environment may also facilitate alternative reaction modes.^15^ This gives rise to a broad repertoire of (stereoselective) biotransformations catalyzed by 2OGDs, such as C–H hydroxylation or halogenation, C–C bond formation, dehydrogenation, epoxidation, heteroatom oxidation, rearrangements,^16,17^ and even redox-neutral C=C bond isomerization.^18^

The mechanism proposed in the literature for Pt2OGD-1 (Scheme 1b)^14^ suggests three subsequent oxidation events of the scaffold in **4** to cleave ring C and install the γ,δ-unsaturated amine in **5**. First, hydrogen atom abstraction from C9 of **4** by the catalytically active iron­(IV)-oxo species in Pt2OGD-1 and hydroxylation by the iron­(III)-hydroxy species via a rebound mechanism activate ring C for hydrolytic cleavage. After ring opening, Pt2OGD-1 was suggested to catalyze another oxidation of ensuing amino aldehyde **8** to furnish amino acid **9**, which is decarboxylated in a third oxidative step by the same enzyme, Pt2OGD-1, leading to the final olefin product **5**. Indeed, some multifunctional 2OGDs are known to catalyze more than one oxidation of the same substrate scaffold, e.g., the dioxygenase AsqJ in quinolone biosynthesis,^19,20^ l-Arg double hydroxylation by OrfP,^21^ and a trifunctional clavaminate synthase.^22^ However, the hypothesized sequence in Scheme 1b would require the consecutive presentation of different types of hydrogen atoms to the iron­(IV)-oxo species of a putative multifunctional Pt2OGD-1. This would necessitate significant substrate promiscuity of the active site to harbor the substrate and the different proposed intermediates. Regeneration of the active iron­(IV)-oxo species is contingent upon the release of succinate, followed by binding of 2-oxoglutarate, which often is the rate-limiting step in the catalytic cycle. Usually, the oxidized product also diffuses out of the active site to be replaced by another substrate molecule before activation of molecular oxygen can occur.^23^ Although Pt2OGD-1 and the associated Pt2OGD-3 are known to accept also C8–C15 saturated substrate derivatives (**10**, **11**),^14^ the significantly different polarity, acidity, and flexibility of species **4**, **8** and **9** make it challenging for a single receptor to recognize and bind each in a productive way. In a metabolic context, this would lead to a decreased efficiency due to the loss of intermediate material. Consequently, we investigated alternative mechanistic pathways for this intriguing biotransformation.

## Results and Discussion

To evaluate the feasibility of hydrogen abstraction at a site different from that of C9, docking studies with computed models of Pt2OGD-1 and N-desmethyl-β-obscurine (**11**) were conducted first. The 2-OG/Fe complex was transplanted from experimentally determined 2-OGD structures with a similar fold pattern to two different models of Pt2OGD-1 AlphaFold2 ^24,25^ and ESMfold^26^ followed by geometry optimization by YASARA^27^ (see the Supporting Information). The two models differed significantly in the positioning of the N-terminal loop (residues 1–14), which is predicted with low confidence in both cases (Figures 1a, and S4). In the AlphaFold2 model, the loop blocks the entrance to the active site and interacts with residue Arg211 close to the iron center, significantly constraining the space for any substrate to be docked (Figure S3).

**Figure 1:**
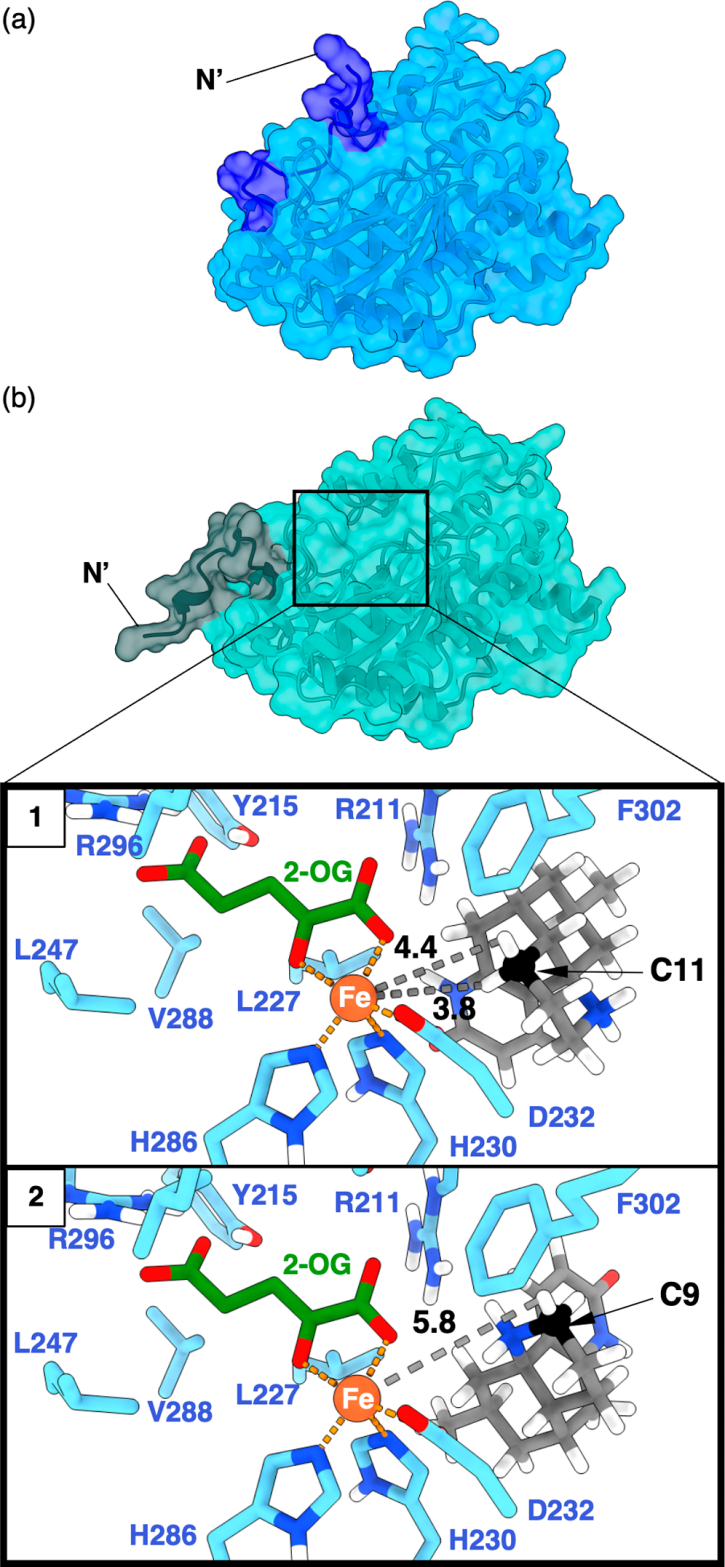
Model comparison and docking study. (a) AlphaFold2 model of Pt2OGD-1 with *N*-terminal loop in blue. (b) ESMFold model of Pt2OGD-1 with the *N*-terminal loop highlighted in gray. Zoom inset: Highest-scoring docking poses of **11** in the ESMFold model presenting the hydrogen atoms on C11 (1) and C9 (2) to the iron­(II)-center (orange) C9 and C11 are highlighted in black. Because the coordination site of the ferryl-oxygen cannot be reliably localized in the model,^30^ the distance between the ligands and the iron center is given instead as gray dashed lines and black numbers (in Å) for comparison.

In contrast, the ESMFold model positioned the N-terminus toward the surface and provided a more spacious active site cavity akin to experimental structures of similarly folded thebaine demethylase from the morphine biosynthesis pathway.^28,29^ With the latter structure model, two binding poses were obtained from docking that present either the C9–H or the C11–H protons of **11** toward the iron­(II)-center (Figure 1b, all docking poses are shown in Figure S2). Pt2OGD-1 is known to have a relaxed substrate specificity regarding the unsaturation at C8 and C15 ^14^ and docking of compound **4** yielded similar results, suggesting that desaturation of the C8–C15 bond would not impact site selectivity for C–H abstraction much (Figure S2). Although the accuracy of these computed models may not be perfect, they led us to think about an alternative mechanism enabled by a binding mode in which C–H abstraction would occur at C11 rather than at C9 as proposed in the previous reaction pathway in Scheme 1b.

We hypothesized that a single C–H oxidation at C-11 could potentially trigger a retrocyclization of ring C to arrive at the natural product casuarinine H (**14**), avoiding multiple diffusion of distinct intermediates into and out of the active site (Scheme 1c). In the process, the C9–C10 bond in ring C would be broken to form the terminal C10=C11 double bond and the formiminium moiety in **13**. This species would hydrolyze spontaneously to release **14** and formaldehyde as a coupled product. As formaldehyde is not part of the literature-proposed reaction sequence (Scheme 1b), detection of this coupled product would support the presence of formimine species **13** and disfavor the stepwise mechanism, which proposes the extrusion of C9 as CO_2_.

We thus started the experimental mechanistic study by attempting to detect formaldehyde in the reaction with recombinant Pt2OGD-1 from Escherichia coli. Given its synthetic accessibility via a convergent seven-step synthetic route from (+)-pulegone,^31^ we selected the alkaloid *N*-desmethyl-β-obscurine (**11**) with a saturated C8–C15 bond as substrate for the biotransformations. Because the obtained dioxygenase biocatalyst displayed its highest activity when used as a lyophilized whole-cell biocatalyst, we first tried to measure nascent formaldehyde in the headspace of the Pt2OGD-1-catalyzed biotransformations. For this purpose, a matrix-robust headspace GC-MS assay for the detection of nascent formaldehyde from whole-cell biotransformations was developed that allowed its indirect quantification in the aqueous phase after derivatization to a fluorinated benzyl-formaldoxime compound. As a background control, samples devoid of cell preparation and/or substrate were analyzed. While the amount of formaldehyde was negligible in the absence of E. coli cell preparation and substrate (Figure S17), a significant background formaldehyde signal from the biocatalyst preparation had to be considered. Upon performing the biotransformation with substrate **11** and Pt2OGD-1, a clear signal for formaldehyde formation in the biotransformation was detected. In another control sample, the biocatalyst preparation was spiked with a defined amount of formaldehyde and incubated under the biotransformation conditions. After subtracting the cell background signal, about 37% of the spiked analyte was recovered, which suggests that endogenous enzymes in the E. coli whole-cell preparation, like dehydrogenases, consume part of the formaldehyde (Table S5).

To confirm these results in the absence of interfering cell background, we purified Pt2OGD-1 and quantified the amount of formed formaldehyde spectrophotometrically after derivatization to a purple dye (Figure 2a).^32^ Indeed, formation of approximate equimolar amounts of formaldehyde and **14** was detected (450 vs 560 μM, respectively) (Figure 2b).

**Figure 2:**
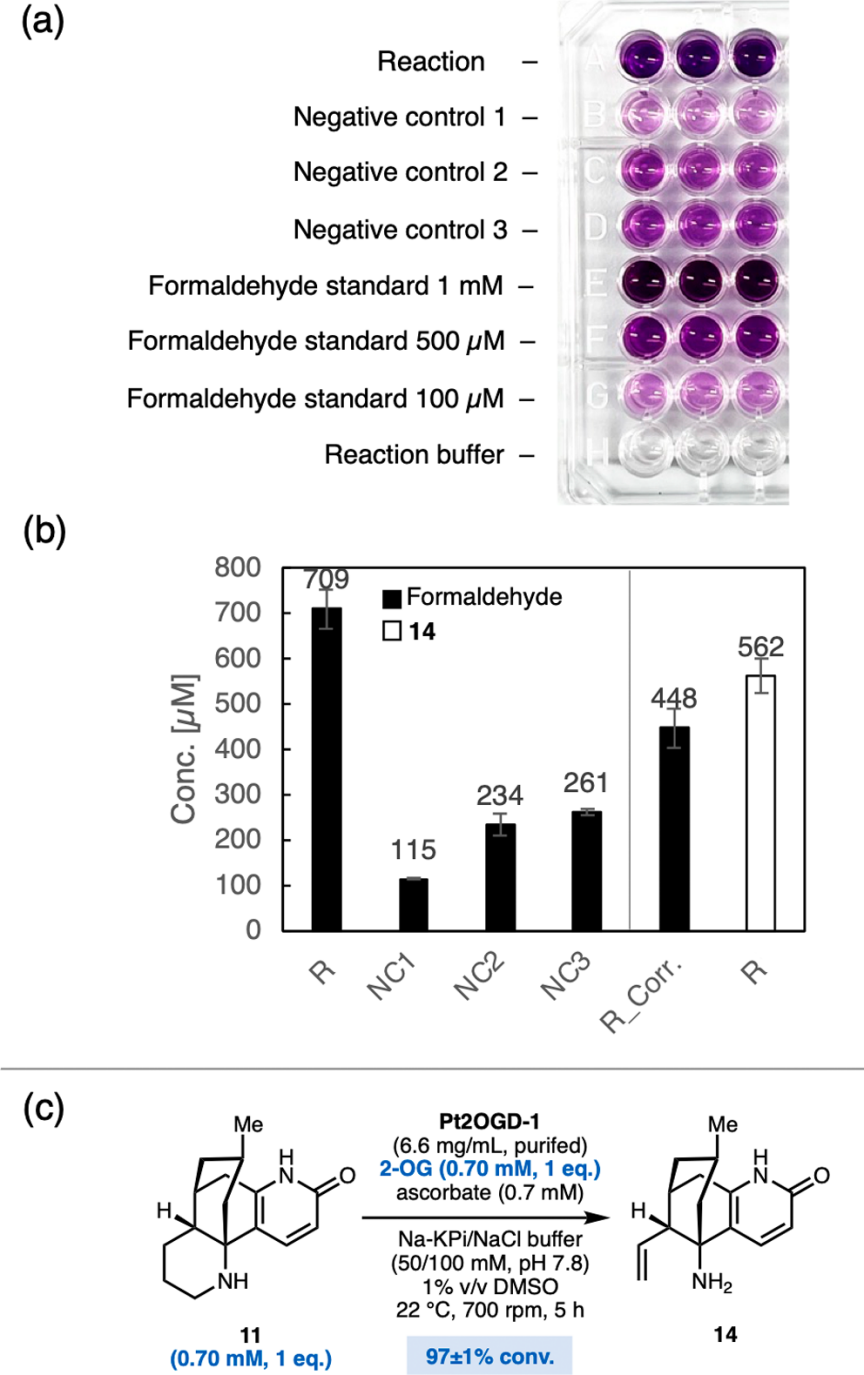
(a) Detection of formaldehyde in Pt2OGD-1-catalyzed biotransformations of **11** with a Purpald assay (performed in triplicate). (b) “R”: Reaction containing substrate **11** (500 μM), purified Pt2OGD-1 (14.2 mg/mL), DMSO (2% v/v), 2-oxoglutarate (1.5 mM), sodium ascorbate (1.5 mM), glycerol (5% v/v), dithiothreitol (25 mM), and NaCl (100 mM) in potassium phosphate buffer (50 mM, pH 7.8). “NC1”: negative control without enzyme Pt2OGD-1; “NC2”: negative control without substrate **11**, “NC3”: negative control without 2-oxoglutarate cosubstrate and ascorbate. Error bars correspond to triplicate experiments. “R_Corr.”: “R” corrected for unspecific background color formation in “NC3”, (c) 97% conversion of **11** was observed with only one single equivalent of 2-oxoglutarate cosubstrate.

Furthermore, the stoichiometry of the reaction was evaluated, as the single-step oxidation of **11** to **14** would require only one equivalent of 2-oxoglutarate (Scheme 1c) while the previously proposed reaction sequence required three equivalents (Scheme 1b). Indeed, when using just one single equivalent of 2-oxoglutarate with purified Pt2OGD-1, a conversion of 97 ± 1% of **11** to mainly **14** was reached, supporting that one equivalent of 2-oxoglutarate is sufficient and the transformation occurs via one oxidation step (Supporting Information, Section 6.4). Together, these findings strongly support that the formation of **14** is induced by a single oxidation event and that formaldehyde is formed as a coupled product, thereby supporting the mechanistic proposal shown in Scheme 1c.

To evaluate the energetic feasibility of the proposed reaction in Scheme 1c, we used density functional theory (DFT) calculations (see the Supporting Information for computational details). A simple model of the iron­(IV)-oxo metal site was used in which the ligands are two imidazoles and two acetates (Figure S13). This coordination sphere is akin to other 2-ketoglutarate-dependent nonheme iron enzymes^33,34^ and is intentionally designed to be as simple as possible to examine the inherent reactivity and avoid bias in the absence of experimental structural information about the active site of Pt2OGD-1.

Using this model, the calculations show that abstraction of the C11 hydrogen of the *N*-desmethyl-β-obscurine substrate (**11**) by the iron­(IV)-oxo species to generate the iron­(III)-hydroxyl compound and a substrate radical has a very feasible barrier of 9.3 kcal/mol (Figure 3a), and the reaction is endothermic by 1.3 kcal/mol.

**Figure 3:**
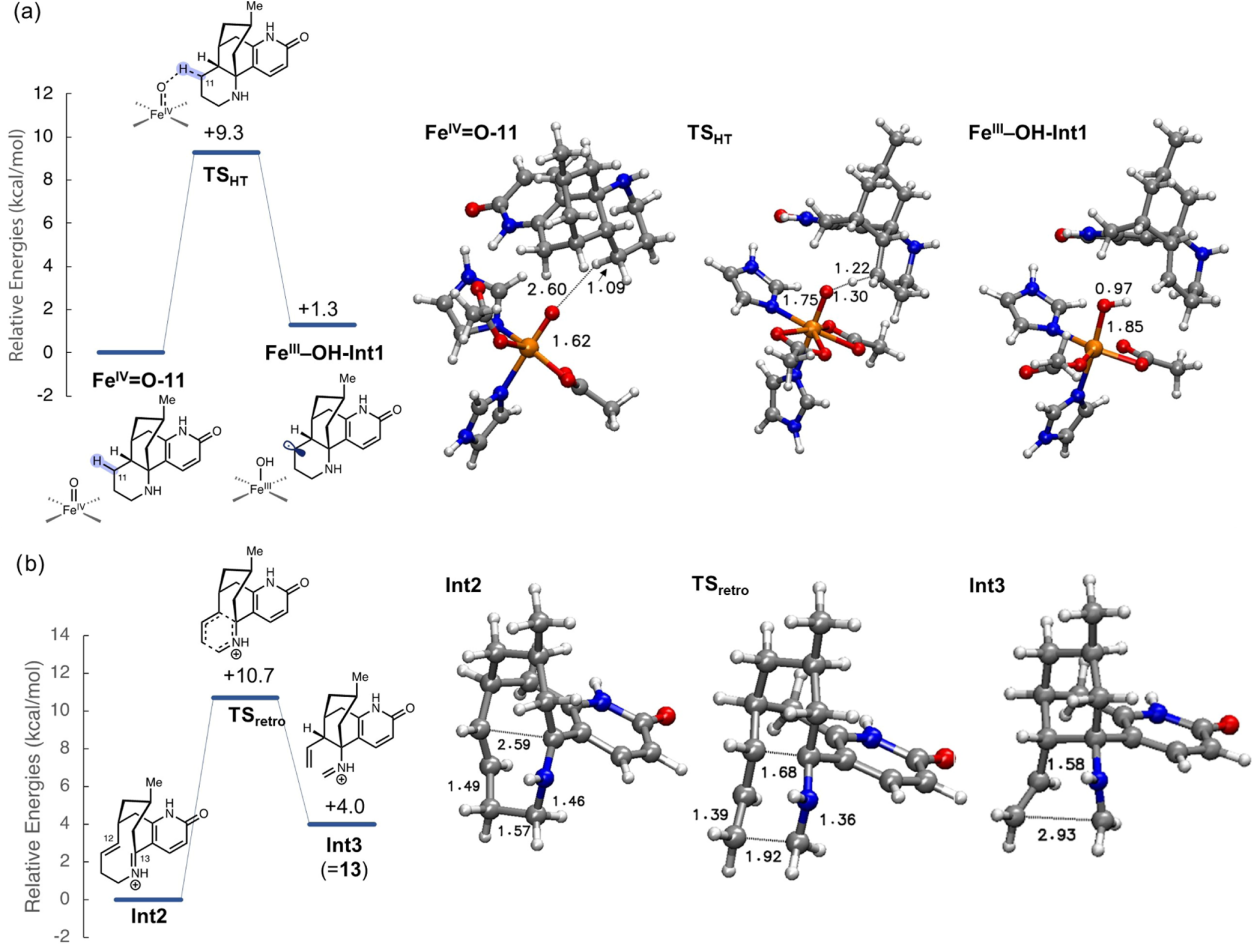
Calculated energy profile and associated optimized structures for (a) the C11 hydrogen abstraction and (b) bond cleavage of the substrate cation.

For the next step, we considered two options, namely, that the C9–C10 bond is cleaved subsequently homolytically or that electron transfer may occur. The calculations showed that a homolytic cleavage of the C9–C10 bond from the substrate radical has a high barrier of 24.1 kcal/mol and can be ruled out (Figure S14). Instead, electron transfer may occur from the substrate radical to the metal center to generate an iron­(II)-hydroxyl compound and a cationic intermediate of the substrate. This process is estimated to be slightly exothermic (−0.4 kcal/mol), and the calculations show that the resulting cation undergoes either a spontaneous ring cleavage to **13** by cleavage of the C9–C10 bond (**Int3** in Figure 3b), or a spontaneous ring expansion by cleavage of the C12–C13 bond (**Int2** in Figure 3B). Just to emphasize, the charge is not observed to be localized at the C11 center.

The transformation starting with formal hydride abstraction followed by opening of the 6-membered ring resembles a retro-aza-Prins reaction. **Int2** is calculated to be 4.0 kcal/mol lower than **Int3**, and the barrier for their interconversion is low (see Figure 3b). This interconversion corresponds to an aza-Cope rearrangement.

Finally, the iminium moiety of **Int3** can conceivably react with a water molecule, inside or outside the enzyme, to produce the final product **14** observed in the experiments along with formaldehyde as a coupled product. This step has not been considered explicitly here, as an overall favorable reaction can be assumed.

For comparison, we also considered the hydrogen atom abstraction from the C9 position of **11** by the iron­(IV)-oxo species, which would correspond to the first step of the previously proposed mechanistic pathway (Scheme 1b). The calculations show that the barrier for this abstraction is very similar to that of the abstraction from the C11 position (9.4 kcal/mol). The resulting radical at the C9 position is about 8 kcal/mol more stable than the radical at the C11 position (Figure S15). Considering the experimental outcomes described above, however, it can be concluded that the enzyme is able to prevent the C9 abstraction, possibly by binding the substrate in an orientation that makes this pathway unviable.

Importantly, we also considered the possibility of a rebound mechanism occurring at the iron­(III)-hydroxy species and the substrate radical on C11, which is a standard step in similar nonheme iron enzymes.^35–40^ Due to the lack of structural information and the smallness of the adopted model, it was not possible here to locate the rebound transition state. However, from previous studies it is known that the step is very fast, with a low calculated barrier that depends on the enzyme environment and the computational methodology.^33,37,38^

Indeed, a side product that matches the mass of a hydroxylated species of **11** (**15**) was consistently detected in LC-MS assays of the Pt2OGD-1 catalyzed reaction (*m*/*z* = 275.1747, Δ – 2.51 ppm, C_16_H_23_O_2_N_2_ [M + H]^+^) (Figures S18 and S55). In line with the expected higher polarity, it elutes earlier from a C18 column than **14** (Figure S18). The observed ratio of putative hydroxylated species and ring-opened product **14** was 1:8.7 at 25 °C (298.15 K, Figure 4a) and more hydroxylation product is formed at higher temperatures (Figure S21). From the reaction course, it can be concluded that **15** is not an intermediate to **14** (Figure S18). The current calculations showed that a rebound step at C11 is very exothermic, by as much as 48 kcal/mol (Figure S16), which suggests that the C11-hydroxylated derivative **15** is a thermodynamically favored off-pathway product.

**Figure 4:**
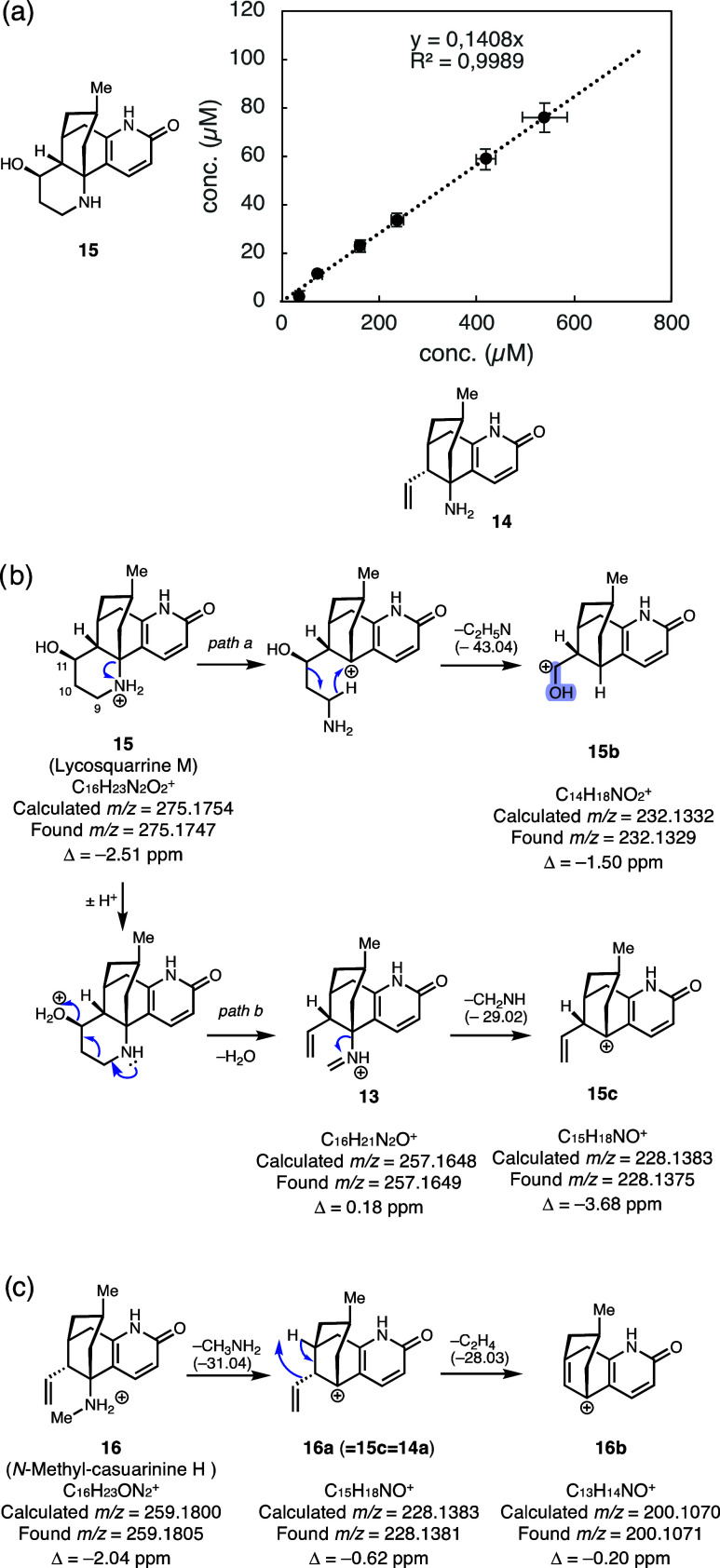
(a) Compounds **15** and **14** are formed in a 1:8.4 ratio at 25 °C, (b) key MS/MS fragmentation events and detected mass fragment ions of hydroxylated off-pathway product **15**, and (c) key MS/MS fragmentation events and detected mass fragment ions of reductively intercepted species **16**.

Detailed analysis of the MS/MS fragmentation pattern and comparison with reported fragmentation patterns of similar hydroxylated alkaloid natural products^41^ supports that hydroxylation occurred on C11 of **11** (Tables S8 and S11). Specifically, detection of a fragment ion matching the molecular formula of **15b** resulting from excision of a C_2_H_5_N unit from ring C suggests that one additional oxygen atom remains in this fragment and hence excludes the possibility of hydroxylation at C9 or C10 (path a in Figure 4b). Another fragmentation sequence that resembles the proposed enzymatic ring cleavage mechanism in Scheme 1c is initiated by the loss of water from **15** to generate a detectable mass ion that matches **13** (or **Int 2**, not shown) (path b in Figure 4b). Instead of imine hydrolysis, which would proceed under aqueous biotransformation conditions, compound **13** cleaves off formimine (CH_2_NH) to yield the observed diagnostic ion with *m*/*z* = 228.138 (**15c**). This matches the fragment ion **14a** (Table S9) in samples containing **14**, suggesting that hydroxylation of **11** enables its transformation to a molecule ion that is in common with ring-cleaved **14** and that is not accessible from nonhydroxylated **11**. Assuming a straightforward fragmentation mechanism without additional hydride shifts, this observation is a direct consequence of hydroxylation occurring at C11.

To characterize the products of the biotransformation, we performed a two-step biotransformation with Pt2OGD-1 and the associated Pt2OGD-3 on preparative scale (100 mg of **10**, see the Supporting Information). Besides the main product casuarinine H (**14**) also a sample of the putative hydroxylated side product **15** was isolated for NMR structure elucidation. Comparison with the analytical data for the known natural product lycosquarrine M isolated from a closely related *Phlegmariurus* plant species^42^ confirmed that hydroxylation has indeed occurred at C11. Particularly, a diagnostic signal corresponding to the α-hydroxy proton on C11 (δH = 3.35 ppm) was detected (Table S12). A ROESY cross-peak for C11–H and C6–H indicates that the C11-hydroxy-group introduced by Pt2OGD-1 is equatorially disposed (*R*-configured) like in the natural product **15** (Figures S42 and S43). Of note, apart from **15**, several other C11-hydroxylated lycodine alkaloids^43–46^ as well as their ring-opened congeners were found in the same plant family, hinting at piperidine-cleaving OGD-1 homologues that have also evolved in other species (Figure S22).

To further corroborate the proposed pathway, we sought to collect experimental evidence of another intermediate along the proposed pathway. As the proposed reaction sequence in Scheme 1c involves the iminium ion species **13**, we hypothesized that this species may be trapped. Running the biotransformation in the presence of sodium cyanoborohydride to intercept this intermediate yielded a new peak at 9.37 min with a molecule ion *m*/*z* = 259.180 that was not present in reactions without reductant (Figure S20). To elucidate whether this peak corresponds to a reductively trapped *N*-methylated congener **16**, the MS/MS fragmentation pattern was analyzed and compared to a reference spectrum of **11**, which lacks the suspected *N*-methyl group (Tables S7, S10, and S11). The major fragment ion **16a** results from the loss of methylamine from the precursor ion and is identical with fragments **14a** and **15c** resulting from the loss of ammonia from **14** and formimine from **13**, respectively (Figure 4c and Table S11). Subsequently, fragmentation of **16a** follows the same pathway as for **14** and yields identical fragment ions in MS/MS spectra (Table S11). The found fragmentation data thus suggests that no other structural alterations took place and supports the presence of a C9-formimine intermediate resulting from the proposed retrocyclization.

## Conclusions

In summary, we collected computational and experimental evidence that the 2-ketoglutarate-dependent dioxygenase Pt2OGD-1 from huperzine alkaloid biosynthesis pathways catalyzes C–C bond cleavage that leads to the opening of a piperidine ring in a single oxidation step. While several examples of oxidative enzymatic C–C bond cleavages^47,48^ and unusual rearrangements involving nitrogen atoms^49,50^ can be observed in nature, the suggested oxidative retro-aza-Prins reaction of a heterocycle is unprecedented for 2ODD-enzymes. Thus, this adds a new reaction to the repertoire of enzymes.

Although aza-Prins reactions have been used for the construction of nitrogen heterocycles in natural product synthesis,^51^ neither a natural nor an artificial retro-aza-Prins reaction has been reported yet. Importantly, this mode of action could inform the design of new oxidative (bio)­catalysts that exert C–C cleavage in heterocycles. Such transformations would also add well to the repertoire of recently developed synthetic methods for precise scaffold diversification of heterocycles in natural products like alkaloids and drug compounds to access derivatives for assessing their pharmacological potentials.^52–54^ Together with the advent of next-generation sequencing technologies, metabolomic studies will reveal ever more enzymes with unusual functions in (plant) biosynthetic pathways such as the one for huperzine A. Our study shows that novel mechanistic pathways can still be found in nature for which no chemical analogue (new-to-chemistry) has been described yet.

## Supplementary Material













## Notes

Pt2OGD-1 ESMFold 4 pose-1 (PDB)
Pt2OGD-1 ESMFold 4 pose-2 (PDB)
Materials; Supporting results; geometry optimized structure; comparison of enzyme models generated by AlphaFold2; computational details; and experimental procedures (PDF)
Pt2OGD-1 ESMFold 11 pose-1 (PDB)
Pt2OGD-1 ESMFold 11 pose-2 (PDB)
Cartesian coordinates of optimized geometries (PDF)
