# Supplementary material for: C–C Bond Cleavage in the Late-Stage Biosynthesis of Huperzine Alkaloids Occurs via Enzymatic Retro-Aza-Prins Reaction
Source: J Am Chem Soc. 2025 May 9;147(24):20265–72. doi: 10.1021/jacs.4c10410 (PMC12186474; doi:10.1021/jacs.4c10410)
Supplement: Supplementary file 3 [file ja4c10410_si_003.pdf]

Supporting Information:

C-C Bond Cleavage in the Late-Stage Biosynthesis of Huperzine Alkaloids Occurs via Enzymatic Retro-Aza-Prins Reaction

Stefan E. Payer,[a,b] Mario Prejanò,[c,d] Philipp Kögl,[a] Tamara Reiter,[a] Eva-Maria Pferschy-Wenzig,[e] Fahmi Himo[c]* and Wolfgang Kroutil[a,f]*

[a]Institute of Chemistry, University of Graz, BioTechMed Graz, Heinrichstrasse 28, A-8010 Graz, Austria.

[b]Enzyan Biocatalysis GmbH, Stiftingtalstraße 14, A-8010 Graz, Austria.

[c]Department of Organic Chemistry, Arrhenius Laboratory, Stockholm University, SE-106 91 Stockholm, Sweden.

[d]Dipartimento di Chimica e Tecnologie Chimiche, Università della Calabria, Via P. Bucci, 87036 Rende, Italy.

[e]Institute of Pharmaceutical Sciences, Pharmacognosy, University of Graz, Beethovenstrasse 8, A-8010 Graz, Austria.

[f]Field of Excellence BioHealth – University of Graz, 8010 Graz, Austria.

Corresponding Authors

Fahmi Himo fahmi.himo@su.se

Wolfgang Kroutil wolfgang.kroutil@uni-graz.at

Table of Contents

1. General Information 4

2. Docking Studies with Computational Models of Pt2OGD-1 5

3. Protein- and Gene Sequences 12

4. Preparation of Biocatalysts 14

4.1. Expression in *E. coli* 14

4.2. Ni-affinity purification 14

4.3. Determination of Pt2OGD-3 activity 15

4.4. Determination of Pt2OGD-1 activity 16

5. Analytical Procederes 17

5.1. Method A: LC-MS analytics 17

5.2. Method B: HPLC-DAD analytics 17

5.3. Method C: UHPLC-HRMS/MS analytics 18

5.4. Method D: Headspace GC-MS 19

5.5. Method E: Purpald® Assay for the colorimetric detection of formaldehyde 21

5.6. Computational Details 22

6. Supplementary Results 25

6.1. Detection of formaldehyde as coupled product in headspace GC-MS assays 25

6.2. Detection of formaldehyde as coupled product with a Purpald® assay. 27

6.3. Time study 28

6.4. 2-Oxoglutarate as limiting component 29

6.5. Reductive interception of reaction intermediates 29

6.6. Temperature study 31

6.7. MS/MS fragmentation analysis of biotransformation products 32

7. Related Alkaloid Natural Products 36

8. Preparative Procedures 37

8.1. Synthesis of *N*-desmethyl-α-obscurine (**10**) 37

8.2. Synthesis of *N*-desmethyl-β-obscurine (11) 37

8.3. Isolation of casuarinine H (14) and lycosquarrine M (15) from a preparative biotransformation .. 38

9. NMR and MS Spectra of Compounds 41

10. References 70

1. General Information

Genes were codon-optimized for expression in *E. coli* and ordered from Twist Biosciences Inc. Disodium ascorbate, α-ketoglutaric acid, formaldehyde (37% w/w solution in water, stabilized with 10-15% MeOH) and pentafluorobenzylhydroxylamine hydrochloride (PFBHA·HCl) were purchased from Sigma Aldrich. (+)-Pulegone was ordered from TCI Chemicals. All other chemicals, reagents and solvents were purchased from commercial suppliers and were used without further purification unless otherwise stated.

Chemical reactions were performed under inert nitrogen atmosphere in oven-dried glassware unless otherwise noted. Room temperature (r.t.) refers to a standard 21 °C. All reactions were stirred with Teflon®-coated magnetic stir bars and were monitored by thin layer chromatography (TLC) performed on silica gel 60 F_{254} coated aluminum plates (Supelco/Sigma Aldrich, Merck KGaA Darmstadt, Germany). Substances were visualized under UV light (λ = 254 nm) and/or staining with basic $KMnO_4$ or *p*-anisaldehyde solutions. Chromatographic purification was performed on silica gel 60 (Millipore/Sigma Aldrich, Merck KGaA Darmstadt, Germany, 0.040–0.063 mm, 230-400 mesh).

^{1}H and ^{13}C-NMR spectra were recorded on a Bruker Ultrashield 300 MHz NMR with a 60-slot BACS auosampler, a BBO-2-channel and 5 mm probe with Z-gradient and ATMA or a Bruker Ascend 700 MHz NMR with a 5 mm triple-resonance cryoprobe. Chemical shifts are reported relative to TMS as parts per million (ppm) and are referenced to the solvent signals [$\delta(^{1}H)$ = 7.26 ppm and $\delta(^{13}C)$ = 77.16 ppm for $CDCl_3$, $\delta(^{1}H)$ = 3.31 ppm and $\delta(^{13}C)$ = 49.00 ppm for MeOH-d4]. Multiplets are reported as follows: s (singlet), d (doublet), t (triplet), q (quartet), dd (doublet of doublet), dt (doublet of triplet), m (multiplet), bs (broad singlet), bd (broad doublet).

2. Docking Studies with Computational Models of Pt2OGD-1

The general workflow followed a published procedure.[1] The ESMFold model was built with the online interface of the Foldseek Search Server[2,3] using the protein sequence of Pt2OGD-1 in FASTA format as input (see section 3).

The AlphaFold2 model was built with the AlphaFold2 plugin in UCSF ChimeraX (V 1.6.1).[4]

A putative iron binding site constituted by His286/His230/Asp232 as well as a potential binding pocket for the substrate was identified in the center of a jelly-roll fold characteristic for many 2OGD enzymes.[5] The confidence score in the region of this putative active site is between 90 and 100 (very good) except for an asparagine (Asn7) that is located on a flexible N-terminal loop and is likely part of a "lid" that covers the active-site entrance (**Figure S4**).

A Foldseek search (3Di/AA mode) with .pdb files of each of the two structure models as query was performed to identify proteins with similar folds. Results were filtered for PDB100 entries with experimentally determined structures (442 hits). With both queries, the highest-ranked protein structure was thebaine 6-*O*-demethylase from *P. somniferum* (E-value of 4.65e–32, 27.3% sequence identity, PDB-ID: 5o9w_A) which was subsequently used as a template for cofactor transplantation.

The "Matchmaker" tool in Chimera X (UCSF Chimera V 1.6.1)[6,7] was used to superimpose the predicted *holo*-Pt2OGD-1 structures (set as reference) and the experimental X-ray crystal structure ("chain pairing mode" with "best aligning pair of chains between reference and match structure"). All atoms of the Fe(II)/2-ketoglutarate complex (in 5o9w_A a Ni- instead of a Fe-atom is present in the crystal structure active site) including water ligands were selected. Unselected atoms were removed. The *holo*-Pt2OGD-1 structure was re-loaded and the two models were combined ("combine" command) and saved as separate .pdb file. The model was loaded in Chimera (UCSF Chimera candidate version 1.15) and the metal geometry was adjusted (Tools > Structure analysis > Metal geometry). His 286.B NE2, AKG 402.A O1, HOH 526.A O, AKG 402.A O5, His 230.B NE2, Asp 232.B OD1 showed the closest distances to the metal ion and were selected as coordinators. Missing bonds were created in the octahedral coordination sphere. The structure editing/building tool in Chimera X (V 1.6.1) was used to change the atom type from Ni to Fe and set the number of bonds to 0. Save structure with transplanted metal ion and co-substrate (Pt2OGD-1_ESMFold_5O9W-Fe2OG.pdb; Pt2OGD-1_AlphaFold2_5O9W-Fe2OG.pdb).

The transplanted structure was relaxed using the YASARA online server for energy minimization (ESMFold structure: start force field energy 735919.9 kJ/mol (score -0.61), final energy: -229637,7 kJ/mol, score 0.13; AlphaFold 2 structure: start force field energy: 68696330920.7 kJ/mol, score -0.90, end: -229238.7 kJ/mol, score –0.05).[8,9] A comparison of the active sites of these transplanted models is shown in **Figure S5**.

Because no plausible binding modes that present either C9 or C11 to the iron center were obtained with this model, other rotamers of amino acid side chains were also considered. While most residues align well with the ones in similarly folded thebaine demethylase (5o9w), Arg211 was modelled as different rotamer. Hence, an additional model with a Arg211 rotamer that is oriented like the homologous Arg219 in 5o9w was manually created in Chimera X (V 1.6.1) by selecting the alternative rotamer (in fact the most prevalent one) from the Dunbrack rotamer library[10] (**Figure S1**c-d). Docking results with this model are shown in **Figure S2**.

A rough geometry for *N*-desmethyl-β-obscurine (**11**) was generated with Avogadro (Version 1.2.0).[11,12] The configuration of stereocenters was adjusted accordingly and the protonation state was adjusted for pH value 7.4. The geometry was optimized using an UFF forcefield with a deepest-descent algorithm (4 steps per update) until the energy difference was ≤0.001 kJ/mol (400.702 kJ/mol). A rough geometry for Huperzine B (**4**) was generated with Avogadro (Version 1.2.0) by selecting the C8–C15 bond in the minimized geometry of **11** and setting the bond-type to double. The protonation state was adjusted for pH value 7.4. The geometry was optimized using an UFF forcefield with a deepest-descent algorithm (4 steps per update) until the energy difference was ≤0.001 kJ/mol (283.397 kJ/mol) (**Figure S1**a).

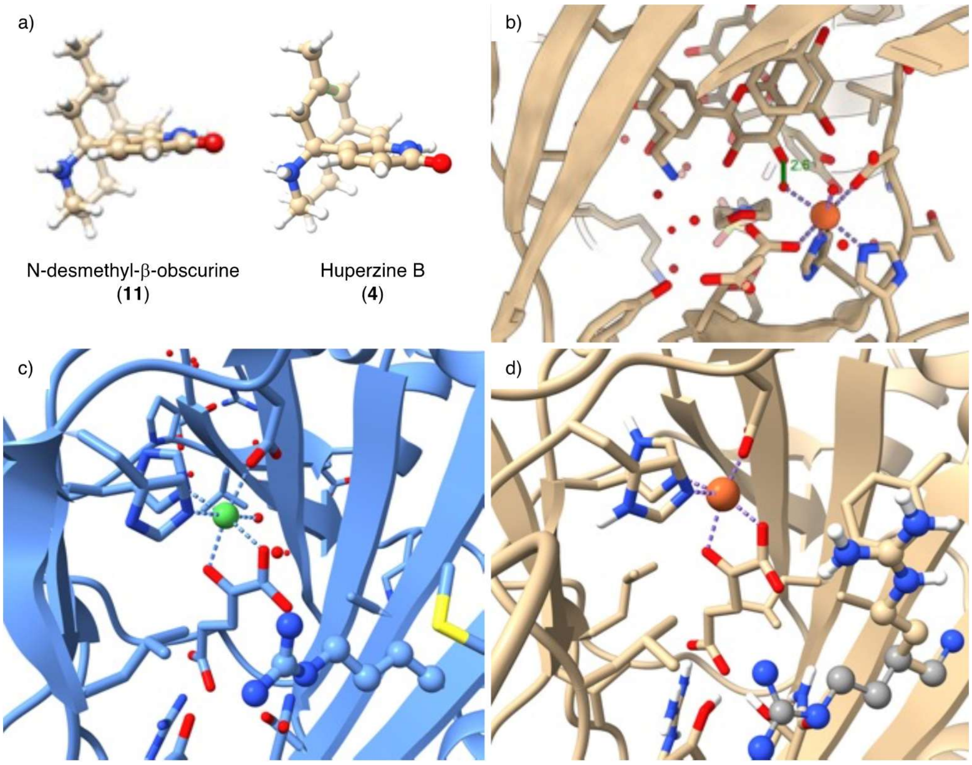

Figure S1. a) geometry optimized structure of **11** and **4** prepared for molecular docking. b) The X-ray crystal structure of anthocyanidin synthase from *Arabidopsis thaliana* complexed with *trans*-dihydroquercetin (PDB-ID: 1GP6) as an example for a typical distance for H-atom abstraction to occur.[13] In the shown X-ray crystal structure, the water ligand located at the previous coordination site of the 2-OG carboxylate oxygen and the carbonyl group in the ferryl species is located 2.6 Å away from the oxidized product (i.e. the position at which the abstracted hydrogen atom was located). c) Active site of Thebaine-O-demethylase (PDB-ID: 5O9W) in blue with Arg219 in ball-and-stick representation. d) Active site of Pt2OGD-1 model (beige) with Arg211 in ball-and-stick representation, the alternative rotamer used for docking is shown in grey.

The receptor protein structure was prepared for docking using the DockPrep plugin in ChimeraX (delete solvents, delete non-complexed ions, standardize certain residue types, replace incomplete side chains, add hydrogens, add charges: +2 for Fe ion, –2 for 2-KG.) and saved as .mol2 file.

Docking was performed in Chimera V 1.15 using the AutoDock Vina plugin.[14] The receptor search volume was centered at the Fe(II) ion (ESMFold-model: 3.742, -2.241, -1.475, AlphaFold2 model: 3.905, -0,857, 2.615), the

search volume size was 20 × 20 × 20 Å. Default settings for receptor and ligand options were kept (all "false"), advanced options: binding modes: 10; exhaustiveness: 8; max. energy difference: 3 kcal/mol). Results are shown in **Figure S2** and **Figure S3**.

a) b) c) d)

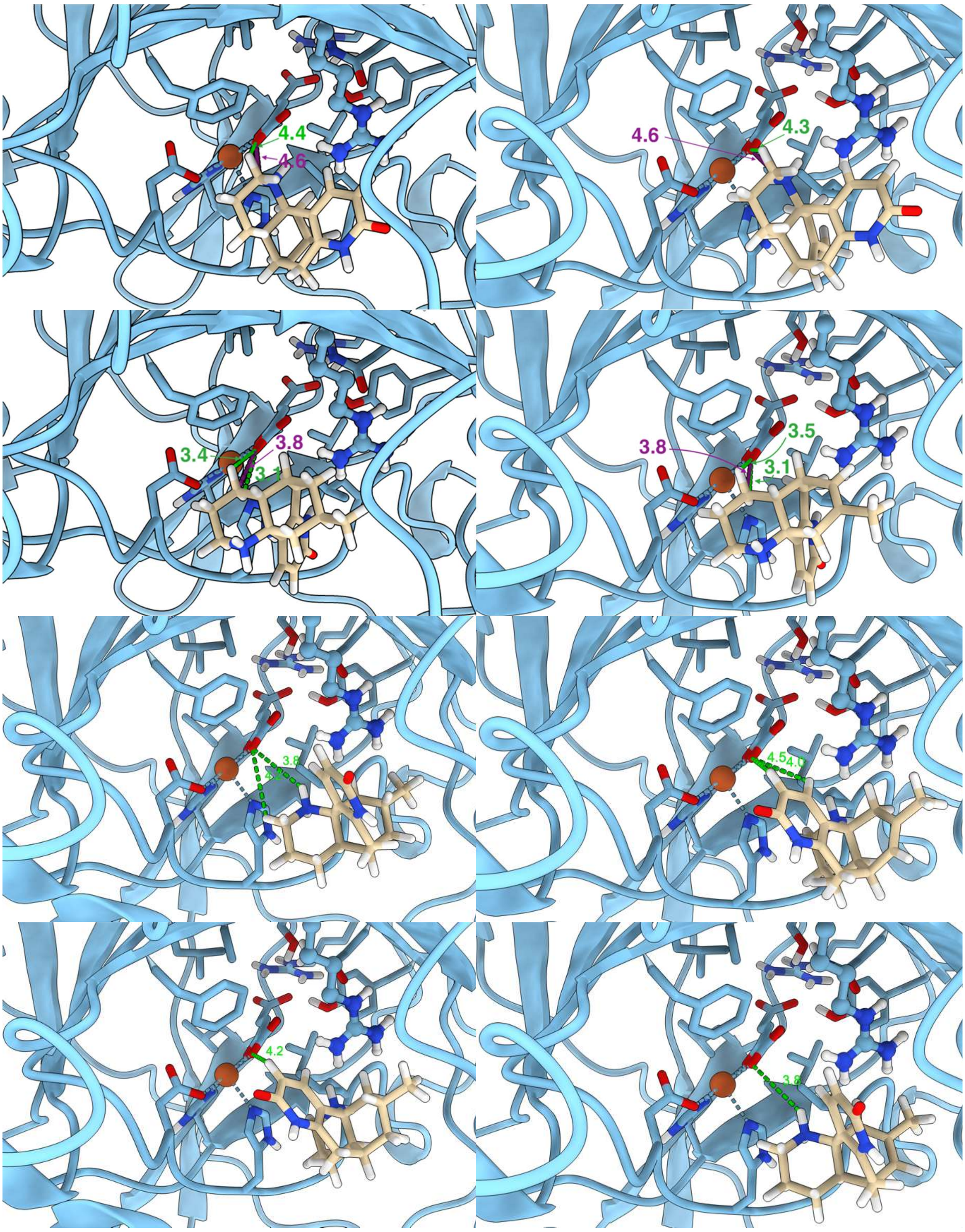

Figure S2. (part 1 of 3)

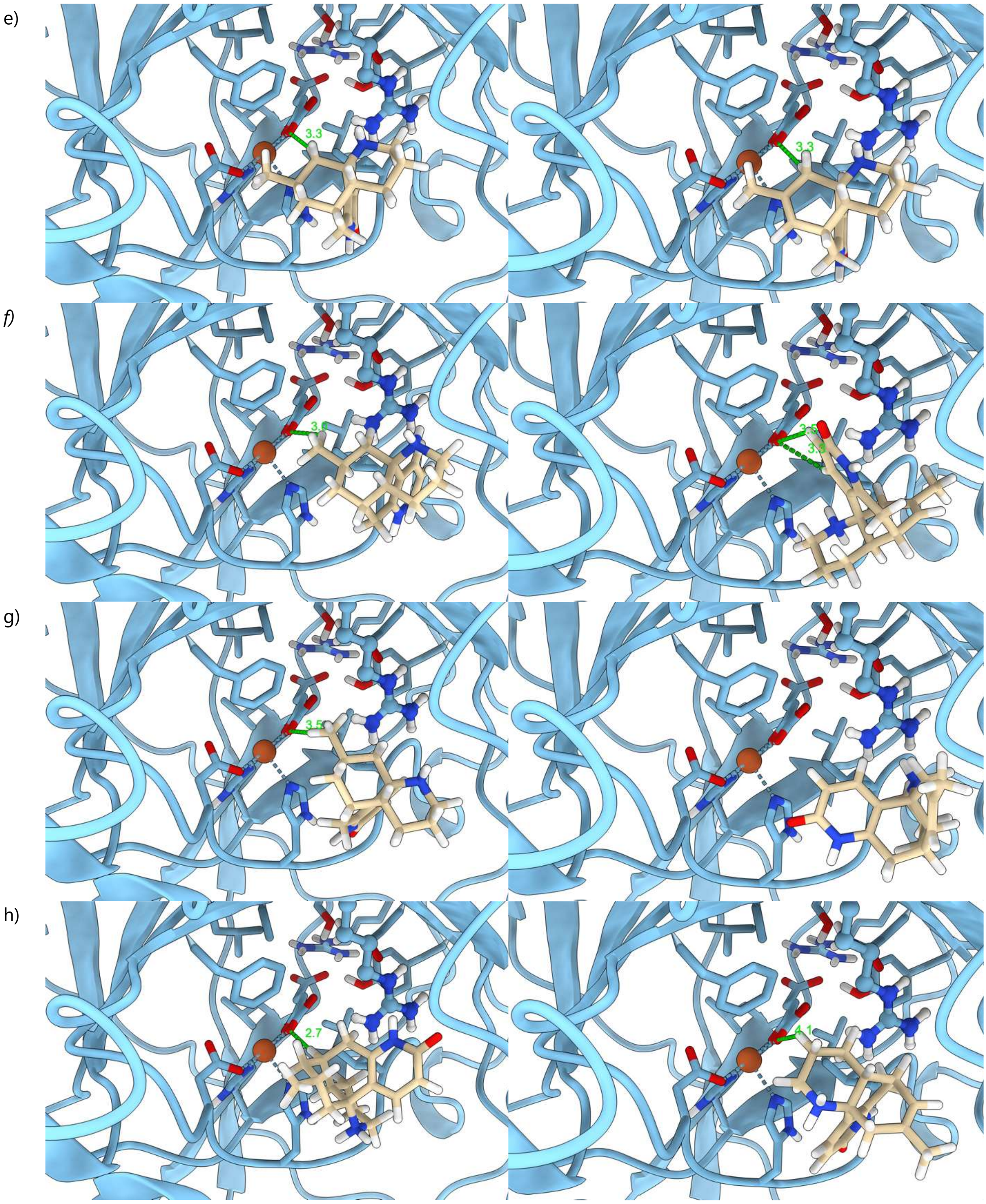

Figure S2. (part 2 of 3)

i)

j)

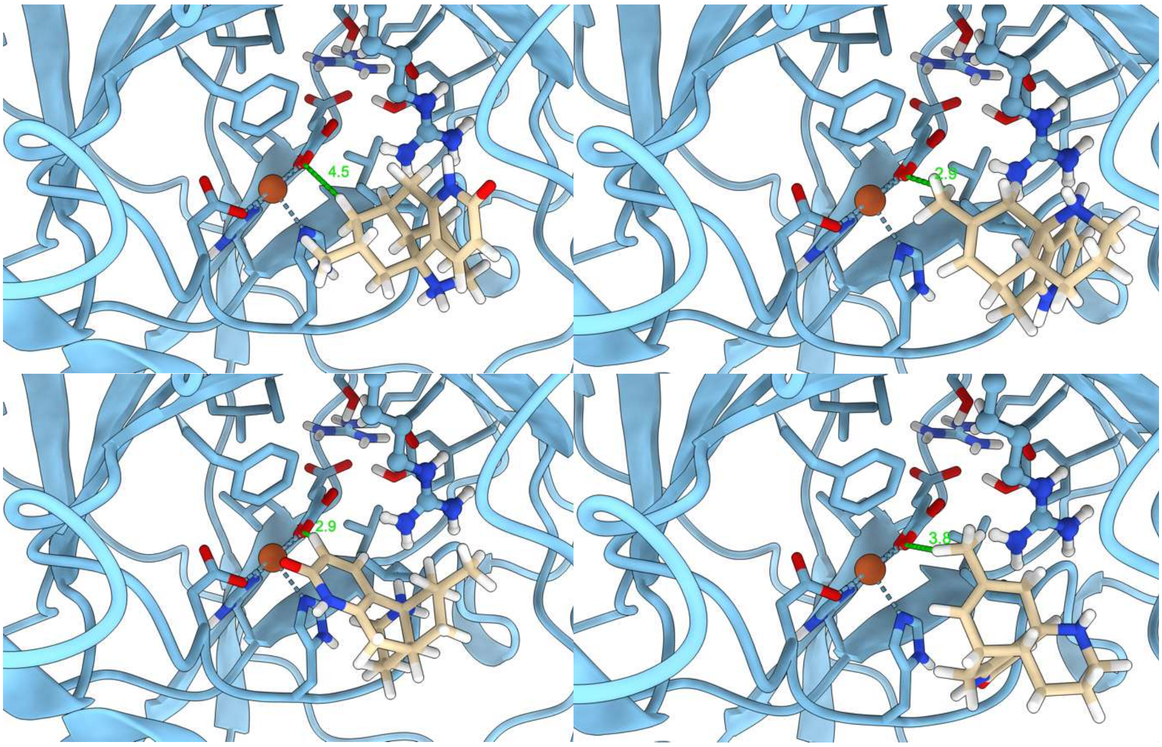

Figure S2. (part 3 of 3) Docking poses of alkaloid ligands (beige) in a Pt2OGD-1 model generated with ESMFold (light blue). Co-substrate 2-ketoglutarate and the Fe(II)-ion (orange) were transplanted from an experimentally determined crystal structure (PDB-ID 5O9W). The alternative rotamer of Arg211 was used and is highlighted in ball-and-stick representation. Left: docking of N-desmethyl-β-obscurine (**11**) ligand, molecular docking scores ΔG (Gibbs free binding energy) a) –8.3, b) –8.0, c) –7.8, d) –7.8, e) –7.5, f) –7.4, g) –7.2, h) –7.1, i) –7.0, j) –7.0. Right: docking of huperzine B (**4**) ligand, molecular docking scores ΔG (Gibbs free binding energy) a) –8.3, b) –8.0, c) –7.8, d) –7.7, e) –7.6, f) –7.4, g) –7.2, h) –7.2, i) –7.2, j) –7.1. Closest distances between *H-atoms* of the ligand and the C1-carboxylate carbonyl oxygen of 2-ketoglutarate that are ≤5 Å are highlighted in green. Closest distances between *C-atoms* of the ligand and the C1-carboxylate carbonyl oxygen of 2-ketoglutarate that are ≤5 Å are highlighted in purple.

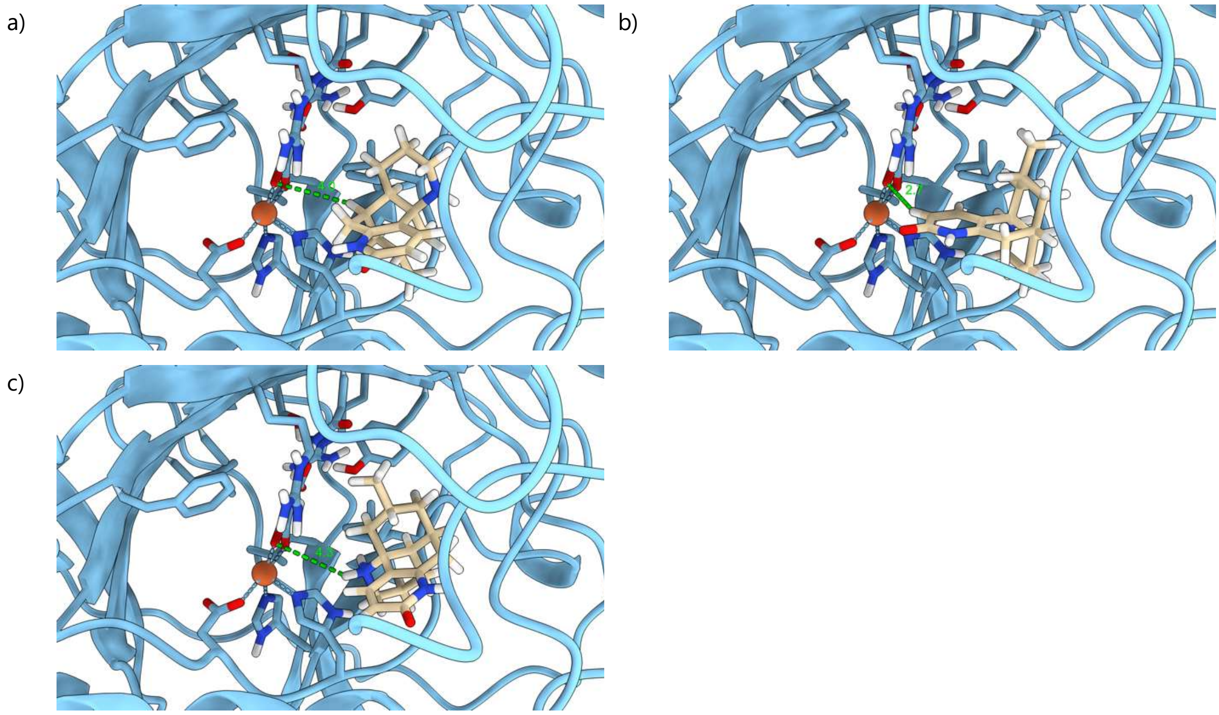

Figure S3. Docking poses of **11** (beige) in a Pt2OGD-1 model generated with AlphaFold2 (light blue). Co-substrate 2-ketoglutarate and the Fe-ion (orange) were transplanted from an experimentally determined crystal structure (PDB-ID 5O9W). Distances between C11–H or C9–H and the C1-carboxylate carbonyl of 2-ketoglutarate that are ≤5 Å are highlighted in green. Molecular docking score ΔG (Gibbs free binding energy) a) 2.8, b) 4.2, c) 4.4.

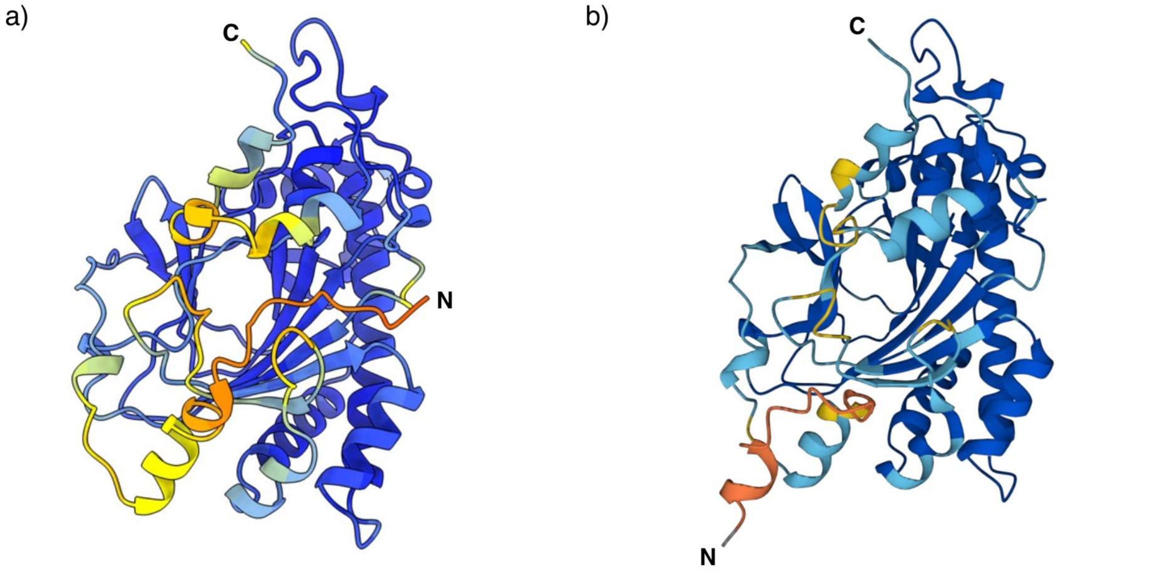

Figure S4. Comparison of enzyme models generated by AlphaFold2 (a) and ESMFold (b). Models are colored according to the pLDDT confidence measure whereas orange indicates low confidence and blue indicates high confidence. The location of the N-terminal loop in the AlphaFold model and the ESMFold model differs significantly.

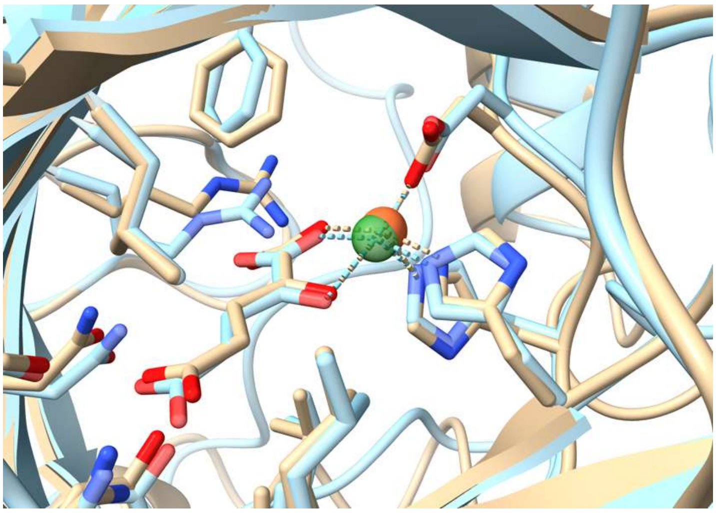

Figure S5. Superimposed models of Pt2OGD-1 after cofactor transplantation and relaxation. The AlphaFold2 model is shown in blue with the Fe(II) ion in green and the ESMFold model is shown in beige with the Fe(II) ion in orange.

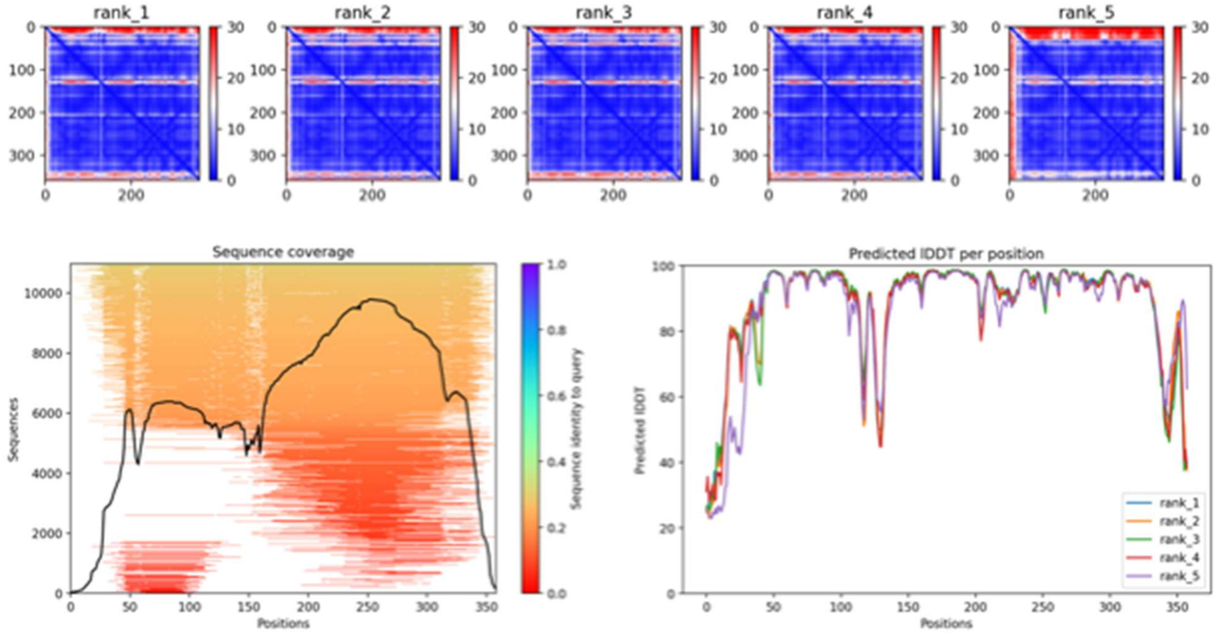

Figure S6. AlphaFold2 prediction performance.

3. Protein- and Gene Sequences

The gene sequences encoding for *Pt*2OGD-1 and *Pt*2OGD-2 were extracted from the NCBI database and further processed within the online gene order interface of the commercial supplier. The sequence with an additional C-terminal stop codon (TAA) was codon-optimized for expression in *E. coli* and inserted into a pET28a vector using the NdeI/XhoI restriction sites to yield a construct encoding for a protein with an N-terminal His_6 tag. The initial Met-encoding codon of the insert was removed because of another ATG codon at the XhoI restriction site that directly precedes the insert.

Below, only the amino-acid sequence of the native protein (i.e. w/o His-tag) is shown. In the gene sequence, lower case letters are part of the vector backbone while the insert is shown in capital letters. Motifs were highlighted as follows: start/stop codon, **His_6 tag**, NdeI restriction site (TA), XhoI restriction site (TCGA).

>Pt2OGD-1 (GenBank: ID: QWQ66225.1, UniProt-ID: A0A8F1NN95)

MGVDVLNARCVGEDNVDTVAVALESGLKLMDDFVWPKEEVEKDNAELSVIDMEKLRKASEADLDELLKDLLRACQEWGFFRIVNH
GVNAELFQKLEEQTLRLFLLPVEAKRNAPCPLNHEIGFYSGADSHIFKHLIWHEGLQLFCDPVCVDEHAQKFWPNGDGEEFSSTIKEY
MKVSQDLGLEILRYLASAVKIDPSKLTKHCTGTNSGLRLNYYPICDRPADTLGLSAHADDDALTILYEDQVGGLQVQRDNKWFAVKPQ
PNTLVVNVGDILQVWSNDLYTSVVHRVVVNNQKRRVSAALFLYPEPESLIEPAAEVIDEDHPCKYKQFPFEEYRVSFYEGDLISKPKSR
FKLIQLPN

>pt2ogd-1::pET28a (NdeI/XhoI; N-term. His_6-tag)
atgggcagcagc**catcatcatcatcatcac**agcagcggcctggtgccgcgcggcagccatATGGGAGTCGACGTTCTTAATGCCCGCTGTGTTGGTGAAGA
CAATGTAGACACCGTGGCCGTGGCCTTAGAGTCTGGATTGAAGTTGATGGACGACTTTGTTTGGCCTAAGGAAGAGGTCGAGAAGG
ACAATGCCGAGCTTAGCGTGATCGATATGGAGAAACTCCGCAAGGCCTCTGAAGCAGACCTTGATGAGTTGTTAAAAGATTTATTGC
GCGCATGCCAAGAATGGGGATTCTTTCGTATTGTGAATCACGGTGTGAACGCAGAGTTATTTCAGAAGCTTGAGGAGCAGACTCTGC
GTCTGTTCTTACTCCCAGTAGAGGCGAAGCGCAACGCCCCGTGTCCATTGAATCACGAGATTGGCTTCTACAGTGGTGCGGACTCAC
ATATCTTCAAGCATCTGATTTGGCACGAGGGACTCCAGTTATTCTGCGACCCAGTTTGCGTCGACGAGCACGCACAGAAGTTTTGGC
CTAATGGGGACGGTGAAGAATTTAGTTCAACCATCAAGGAGTACATGAAGGTCAGTCAAGACCTCGGGTTGGAAATCTTGCGCTAT
CTGGCGTCTGCTGTCAAGATCGATCCCAGCAAACTTACTAAGCATTGCACAGGCACCAATTCTGGGCTGCGTTTGAACTATTATCCG
ATCTGTGACCGCCCCGCGGACACCCTGGGACTTAGTGCCCATGCAGACGACGATGCGCTGACGATTTTATATGAAGACCAGGTGGGC
GGTTTACAGGTGCAACGTGACAATAAGTGGTTCGCAGTGAAGCCACAACCAAACACCTTGGTTGTCAATGTTGGCGACATTTTGCAG
GTGTGGAGTAATGACTTATACACTTCGGTCGTGCATCGCGTGGTCGTCAATAACCAGAAGCGTCGCGTTTCTGCCGCGTTATTCTTG
TACCCTGAGCCGGAGAGCCTGATTGAGCCGGCTGCGGAAGTGATCGACGAGGACCACCCGTGTAAGTATAAGCAGTTCCCATTCGAG
GAGTACCGTGTCTCGTTCTACGAGGGTGACTTAATCTCGAAGCCTAAGTCGCGCTTTAAACTCATCCAACTTCCCAATTAACTcga

>Pt2OGD-3 (GenBank ID: QWQ66227.1, UniProt-ID: A0A8F1SZL1)

MAAVANASASDLPWSVLKMVEMNGSAGGGGRVPNCFIRPVEEAASHSTSLDLRVPVVDMNTFKGSTKTQALQQLASACESWGFFQL
SNHGVPNHLIQQTLQLSRQFFELPFEEKQKYVIDPDCNTDGYGRVNIKSSENDLLDWGDSFVAEYSPASSRNFDKWPMEPAAFRKVV
SAYCDEIEKLSHELFSLFEEALGLPPAYIREVCGEHGLQFGINYYPSCPQPDLVLGLSAHSDNVPIITILIQDEAGLQVRKDGKWVQVDAI
PNHIVVNIADQLEVISNGRFKSVVHRVAVNSLKSRLSFSIFYKPEIGAQIAPAPQLLDELHPALYEERTFSGVSPYAQTRLQEQESLEKIR
LSSRK
>pt2ogd-3::pET28a (NdeI, XhoI, N-term. His_6-tag)

atgggcagcagc**catcatcatcatcatcac**agcagcggcctggtgccgcgcggcagccatATGGCAGCAGTTGCAAACGCATCAGCCAGCGATTTACCTTG
GAGCGTACTTAAGATGGTGGAGATGAACGGTAGTGCCGGTGGTGGCGGGCGTGTGCCTAACTGCTTTATTCGCCCTGTTGAGGAAGC
GGCATCTCATTCTACGAGCCTGGATCTGCGTGTCCCGGTTGTAGACATGAATACCTTTAAAGGCTCAACCAAGACCCAAGCCCTTCA
ACAGCTTGCCAGCGCATGCGAGTCTTGGGGTTTCTTCCAGCTTTCGAATCATGGGGTTCCGAACCACCTCATTCAACAAACCTTGCA
GTTATCACGCCAATTCTTTGAGTTACCATTTGAAGAAAAGCAGAAATATGTGATCGACCCAGACTGTAATACCGACGGATACGGTCG
CGTCAACATTAAAAGCTCAGAGAACGACCTCTTGGACTGGGGTGACTCCTTCGTCGCCGAATACTCACCTGCCAGCAGCCGTAACTT
CGATAAGTGGCCCATGGAACCTGCGGCATTTCGCAAGGTGGTTTCAGCGTATTGCGACGAAATCGAGAAGCTTAGTCACGAGCTGTT
TTCCTTATTCGAGGAAGCCCTTGGCTTACCCCCAGCCTACATCCGCGAGGTGTGCGGGGAACACGGATTGCAATTTGGGATTAATTA
TTACCCGTCGTGCCCGCAACCGGATCTGGTTCTTGGATTGAGCGCCCATAGCGATAATGTTCCGATCATCACGATCCTTATCCAGGAC
GAAGCCGGTCTCCAAGTTCGTAAAGATGGCAAGTGGGTACAAGTGGATGCCATCCCCAACCACATCGTGGTAAACATTGCCGACCAA
CTCGAAGTTATCAGCAATGGTCGTTTCAAGAGCGTGGTGCACCGTGTTGCCGTGAATTCACTGAAGTCTCGCCTTAGTTTCTCTATC

TTTTACAAACCCGAGATTGGGGCTCAAATCGCTCCTGCTCCACAACTGTTGGACGAGCTGCACCCAGCCTTATACGAGGAACGTACT
TTTAGTGGAGTCTCACCGTATGCCCAAACACGTCTTCAAGAACAGGAGTCCTTGGAAAAGATCCGTTTGTCTAGTCGTAAGTAACTc
ga

The expression constructs were stored in the internal plasmid database with identifiers pEG669 (Pt2OGD-1) and pEG670 (Pt2OGD-3).

4. Preparation of Biocatalysts

4.1. Expression in *E. coli*

TB-medium (3 × 330 mL) supplemented with kanamycin (50 µg/mL) in 1.0 L baffled culture flasks were inoculated with 3.0 mL of overnight culture of *E. coli* BL21 (DE3) transformed with the respective 2OGD plasmids. Cells were grown at 37 °C and 120 rpm until an OD600 of ca 0.6 was reached. The flasks were then shaken at 20 °C for 1 h after which IPTG (0.5 mM final conc.) was added, and shaking was continued at 20 °C overnight. The cultures were centrifuged (47,000 × g, 20 min, 4 °C), the pellet was washed with chilled sodium phosphate buffer (50 mM, pH 7.8, 15 mL), resuspended in the same buffer (15 mL) containing 10 mM Fe(II)SO_4, frozen in liquid N_2 and lyophilized for 6 h. The whole-cell preparation was stored at –21 °C until further use. Soluble expression was verified by SDS-PAGE (Genscript, 10% acrylamide, 15 µg protein per well). 5.0 mg of lyophilized cells were carefully suspended in BugBuster reagent (500 µL) and incubated for 15 min at 21 °C Digested cell suspensions were centrifuged (4 °C, 20,000 × g, 15 min). The pellet was separated from the supernatant and resuspended in water (0.5 mL).

4.2. Ni-affinity purification

For purification, the cell pellet was thawed on ice, resuspended in lysis buffer (50 mM KPi, 100 mM NaCl, 50 mM dithiothreitol - DTT, 10 mM imidazole, pH 7.8) and sonicated (Branson Digital Sonifier, 1 sec on, 4 sec off, total time 2:30min, 40% amplitude.). After centrifugation (15 000 rpm at 4°C for 20 min) the supernatant was filtered through a 0.45 µm syringe filter. Purification was done on an ÄKTA Pure system with a HisTrap FF 5ml column (GE Healthcare) and a gradient elution program from lysis to elution buffer (50 mM KPi, 100 mM NaCl, 50 mM DTT, 500 mM imidazole, pH 7.8) (0-100% elution buffer 10 CV, 3 mL/min). Fractions were analyzed by SDS-PAGE and those containing the target enzyme were pooled (**Figure S7**). Buffer exchange to storage buffer was done using 10 kDa MWCO Vivaspin 20 column (Sartorius) by concentrating at and resuspending five times with storage buffer (50 mM KPi, 100 mM NaCl, 50 mM DTT, 10% v/v glycerol, pH 7.8). The concentration of the purified enzymes in storage buffer was measured via absorption at 280 nm and calculated extinction coefficients: 28.4 mg/mL for Pt2OGD-1 (700 µmol/L with a molecular weight of 40725.29 g/mol) and 80.1 mg/mL for Pt2OGD-3 (2.0 mmol/L with a molecular weight of 40014.27 g/mol).

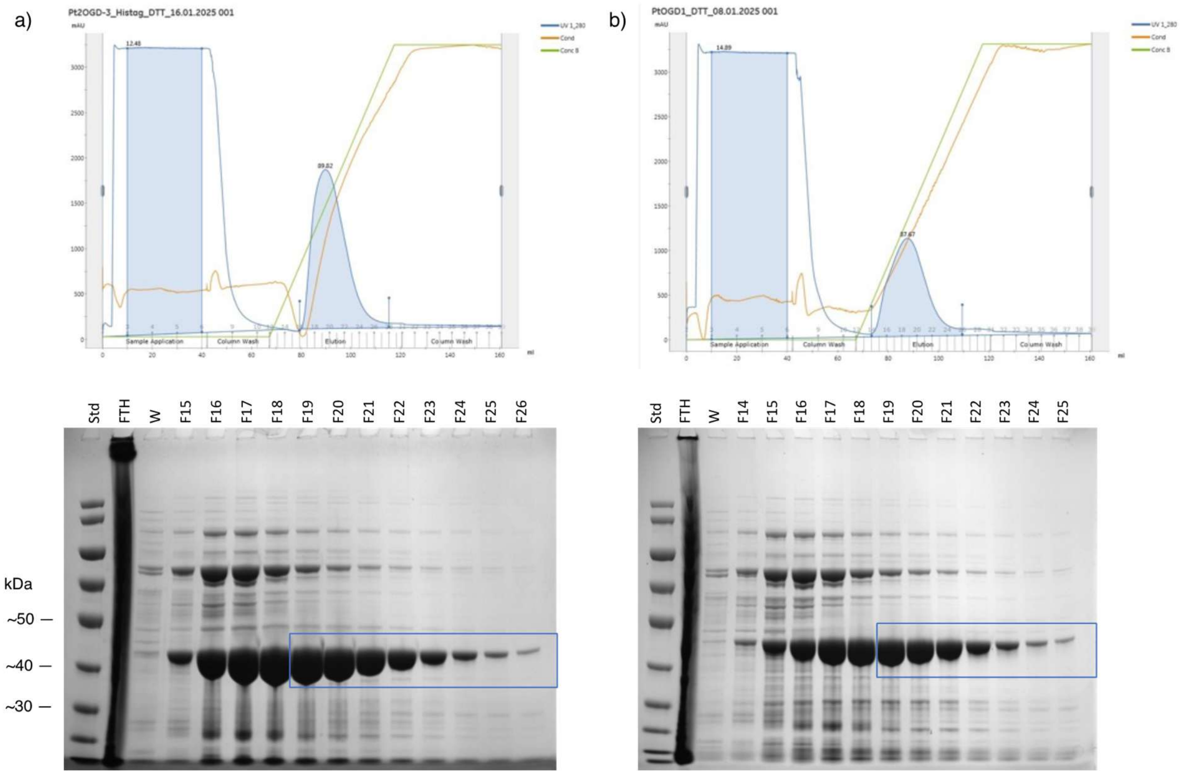

Figure S7. Purification of a) Pt2OGD-3 and b) Pt2OGD-1. Pooled fractions used for further experiments are indicated with a blue box.

4.3. Determination of Pt2OGD-3 activity

Lyophilized *E. coli* cells containing Pt2OGD-3 (5.0 mg) were resuspended in (Na-KPi/NaCl 50/100 mM, pH 7.8; 810 µL) in a 2.0 mL microcentrifuge tube. A combined stock of 2-oxoglutarate and sodium ascorbate (83 mM each, pH adjusted to 7.8 with 1 M NaOH; 180 µL, 15 mM final conc., 15 equiv.) was added, followed by addition of substrate **10** (10 µL of a 100 mM stock in DMSO) (t = 0 min). The reaction vessel was incubated horizontally in a thermoshaker set to 30 °C (450 rpm). Samples (100 µL) were withdrawn after given time intervals (1–40 min) and quenched by mixing with acetonitrile (100 µL, containing 0.1% v/v formic acid). The quenched samples were centrifuged (10 min, 20,000 × g) and the clear supernatant was transferred to plastic HPLC-vials. Conversion of **10** to **11** was measured by HPLC using *Method B*. All time profiles were performed in triplicates. Product **11** was formed at an initial rate of 113.9 µmol L^{-1} min^{-1}. One unit is defined as the formation of 1 µmol substrate **11** per min, which corresponds to a specific activity of 22.8 mU mg^{-1} cells.

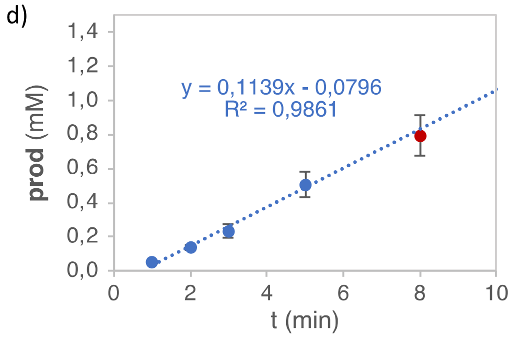

Figure S8. Activity assay of Pt2OGD-3 as whole cell preparation cultivated in TB-medium based on the formation of NDMBO (**11**). Red datapoints were excluded from fit.

4.4. Determination of Pt2OGD-1 activity

In a 2.0 mL microcentrifuge tube, Pt2OGD-3 (5.0 mg lyophilized cell preparation from TB-culture, 112.5 mU) was resuspended in reaction buffer (KPi/NaCl, 50/100 mM, pH 7.8; 840 µL) (30 °C, 700 rpm, horizontally, 5 min). A combined stock of 2-oxoglutarate and ascorbate (83 mM each in reaction buffer with re-adjusted pH 7.8, 150 µL, 12.45 mM final conc., 12.45 equiv.) and substrate **10** (10 µL, 100 mM in hot DMSO) were added (t = –30 min). The closed tube was incubated in a thermoshaker (30 °C, 700 rpm, horizontally) for 30 min. A sample (100 µL) was withdrawn and diluted with acetonitrile containing 0.1 % v/v formic acid (100 µL) (t = 0 min) to confirm complete conversion to **11** before the cell-suspension was transferred to a fresh 2.0 mL microcentrifuge tube containing Pt2OGD-1 (10.0 mg lyophilized cell preparation from TB-culture). Incubation was continued and samples were withdrawn after indicated time intervals (treatment as before). The quenched samples were incubated at room temperature for 30 min and then centrifuged at 20,000 × g) before transfer to a plastic HPLC vial. The samples were analyzed by HPLC using *Method B*. Substrate **11** was consumed at an initial rate of 32.3 µmol L^{-1} min^{-1}. One unit is defined as the conversion of 1 µmol substrate **11** per min, which corresponds to a specific activity of 3.2 mU mg^{-1} cells.

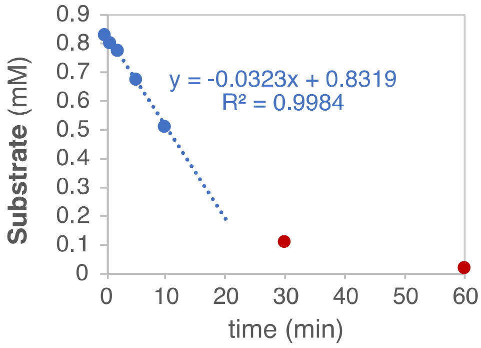

Figure S9. Activity assay of Pt2OGD-1 as whole-cell preparation cultivated in TB-medium based on the consumption of NDMBO (**11**).

5. Analytical Procederes

5.1. Method A: LC-MS analytics

Samples were analyzed on an Agilent Infinity 1260 system equipped with a Phenomenex Luna C18(2) 100A column (250 mm × 4.6 mm × 5 µm). The analytical method consisted of a gradient of 0.1% formic acid in water ("A") and 0.1% formic acid in acetonitrile ("B"): 2 to 50% B (10 min), 50% B (2 min), 50 to 2% B (1 min), 2% B (2 min), 1 mL min^{-1}, 5 µL injection. The analyte flow from the column was split in a 1:1 ratio between a variable wavelength detector (VWD, λ_{det} = 245 nm) and an Agilent 6120 quadrupole mass selective detector (API-ES positive, 3000 V, capillary voltage; scan mode *m/z* = 100–400, fragmentor set to 40). Single ion spectra of *m/z* ratios corresponding to the expected products and intermediates were extracted and peaks were integrated (see **Figure S55** for example chromatograms).

5.2. Method B: HPLC-DAD analytics

Samples were analyzed on a Shimadzu LC20AD HPLC system equipped with a Phenomenex Luna C18(2) 100A column (250 mm´× 4.6 mm´× 5 µm). The analytical method consisted of a 0.7 mL/min isocratic flow of aq. NH_4OAc (80 mM in HPLC-grade water, pH adjusted to 6.0 with acetic acid, glacial, ultra-filtrated, degassed in ultrasonic bath) and methanol (HPLC-grade) in 7:3 ratio at 35 °C. 10 µL sample was injected and the acquisition time was set to 18 min. While **10** could be selectively detected at 250 nm (t_R = 13.5 min), **11** and **14** feature an absorption maximum at 310 nm (t_R = 11.2 and 12.2 min, respectively) (see **Figure S54** for example chromatograms). All compounds can however be detected simultaneously at 230 nm.

Calibration standards (10 µM – 500 µM) with pure synthetic *N*-desmethyl-α-obscurine (**10**), *N*-desmethyl-β-obscurine (**11)** in acetonitrile/water 1:1 (+ 0.1% v/v formic acid) and casuarinine H (**14**) in ammonium acetate buffer/MeOH 7:3, were prepared. Stock-solutions (2 mM) were prepared by dissolving the reference compounds in the respective solvents on an analytical scale (±0.1 mg). From this stock solutions dilutions (0.5 – 0.1 mM) were prepared on the scale and dilution factors were derived from the mass of used stock solution and the total mass after dilution. Low-concentration standards (75 µM – 10 µM) were prepared by diluting the 100 µM stock.

Calibration standards were measured with the analytical method from low to high concentration. Areas of peaks in the respective absorption channels were extracted and plotted against the exact standard concentrations. For biotransformations with 1 mM substrate loading, samples (100 µL) are usually diluted with MeCN+0.1% formic acid (100 µL, 1:1), which adjusts the analyte concentration to the measurable range.

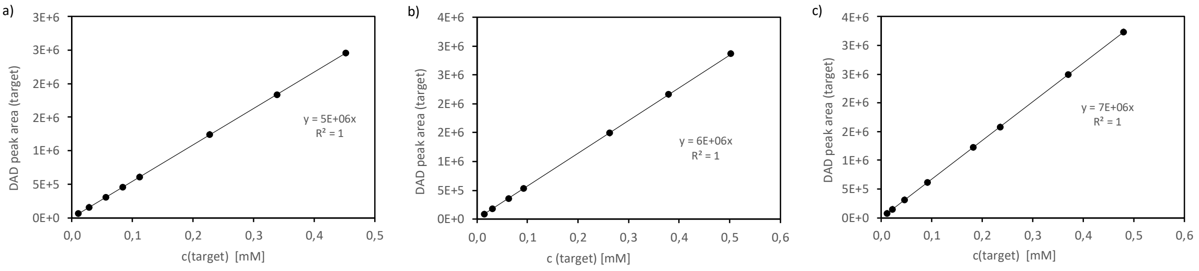

Figure S10. HPLC-DAD calibration curves for a) NMDAO (**10**) at 250 nm, b) NDMBO (**11**) at 310 nm and c) casuarinine H (**14**) at 310 nm. The y-intersection was forced through 0.

The analyte concentration in the biotransformation (conc, 2-fold dilution considered) is thus calculated by the following formula

$$A = (a \cdot conc) \cdot 2$$

with A as the peak area in the corresponding extracted UV trace and a, b derived from the calibration curve (**Table S1**).

Table S1. HPLC-DAD calibration coefficients.

	NDMAO (**10**) (250 nm)	NDMBO (**11**) (310 nm)	Casuarinine H (**14**) (310 nm)
a	5.44×10^6	5.69×10^6	6.73×10^6

5.3. Method C: UHPLC-HRMS/MS analytics

Samples were analyzed on a Thermo QExactive Hybrid Quadrupole Orbitrap mass spectrometer coupled with a Dionex UltiMate 3000 UHPLC system. Separation of alkaloid products was achieved on a Waters Acquity Premier BEH C18 column (1.7 µM, 2.1 x 100 mm) using the following mobile phase composition: solvent A: aqueous ammonium acetate solution (10 mM, pH 6.0 adjusted with acetic acid); solvent B: acetonitrile. Flow gradient: 0–2 min 5–9% B; 2–20 min 9% B; 20–22 min 9–20% B; 22–24 min 20–30% B, 24–26 min 30% B; 26–26.5 min 30–5% B, 26.5–30 min 5% B; flow: 0.2 mL min^{-1}, column temperature: 40 °C, sample injection: 0.5 µL. UVVIS absorption was monitored by DAD (wavelength range 210-450 nm). The heater temperature of the HESI-II source was 250 °C, capillary temperature was 300 °C, spray voltage was 3 kV, sheath gas flow was 35, and auxiliary gas flow 5 arbitrary units, and S-lens RF level was 60 V.

Signals were acquired in full MS / dd-MS^2 positive ionization mode (default charge 1) using the following settings: **Full MS**: resolution 70,000 (FWHM at *m/z* 200); AGC target: 3E6; Maximum inject time: 100 ms; scan range: 100 – 700 *m/z*. **dd-MS^2** : resolution: 17,500 (FWHM at *m/z* 200); up to 5 MS^2 scans per cycle, TOP5 most abundant MS^1 scans selected; AGC target: 1E5; Maximum inject time: 50 ms; Isolation window 2.0 *m/z*; stepped normalized collision energy: 18, 30, 45; dynamic exclusion: 3.0 s. Data were evaluated with XCalibur 4.4 (Thermo), and Freestyle 1.3 was used for molecular formular calculation.

5.4. Method D: Headspace GC-MS

For quantification of derivatized formaldehyde, standard solutions of formaldehyde in Na-K phosphate/NaCl buffer (50/100 mM, pH 7.8) (1.0 mM, 750 µM, 500 µM, 250 µM and 100 µM) were prepared in headspace GC-vials (20 mL, Agilent, Part No. 5182-0837) and immediately sealed with a Teflon®-coated septum and a crimp cap. For derivatization to the corresponding formaldoxime,[15] a solution of pentafluorobenzylhydroxylamine hydrochloride (PFBHA·HCl, 100 mM in dH_2O, 50 µL, 5 mM final conc., 5 equiv.) was added through the septum with a microliter syringe. The vials were then placed in a drying oven set to 60 °C for 60 min before subjecting to headspace GC-MS measurements with an Agilent 7697A headspace sampler unit connected to an Agilent 7890 GC system and an Agilent 5975C inert XL mass-selective detector using the method parameters shown in **Table S2**.Integrals of the analyte peaks in the SIM mode (t_R = 7.59 min; mass ions with m/z 181 and 195) were plotted against the exact concentration of formaldehyde in the used standard-solution (**Figure S11a**). A linear regression (R^2 = 0.999) yielded the following calibration function:

$$c_{formaldehyde}\ [\mu M] = \frac{(Area_{181,195})}{77434}$$

Table S2. Instrument parameters for the GC-MS headspace measurements.

**GC-MS**	
Column	HP-5MS (Agilent) 5% Phenyl Methyl Silox 30 m × 250 µm × 0.25 µm film
Temperature progam	60 °C (2 min) — 7 °C min^{-1} — 150 °C — 15 °C min^{-1} — 220 °C
Carrier gas	He
Inlet Temperature	200 °C
Inlet He pressure/flow	4.4867 psi / 10.7 mL min^{-1}
Split ratio/flow	10:1 / 7 mL min^{-1}
Mass range (Scan)	31–300
Threshold	150
Monitored ions (SIM)	181.00; 195.00
Ionization energy	70 eV
Ion source temperature	230 °C
Quadrupole temperature	150 °C

**Headspace autosampler**	
Loop size	1 mL
Vial oven temperature	60 °C
Loop temperature	120 °C
Transfer line temperature	180
Vial equilibration time	2 min

Injection duration	0.08 min
GC cycle time	22 min
Vial shaking	on (71 shakes min^{-1})
Fill pressure	15 psi
(Loop) Fill mode	default

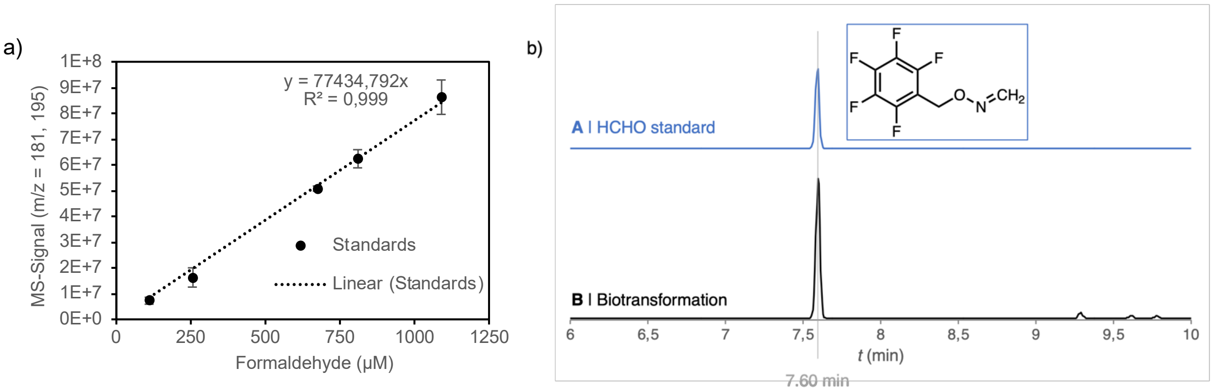

Figure S11. a) Calibration of the headspace GC-MS method. The formaldehyde concentration in the measured samples is within the linear range. Error bars correspond to triplicate samples. b) The formaldoxime species from the biotransformation matches the derivatized formaldehyde standard.

5.5. Method E: Purpald® Assay for the colorimetric detection of formaldehyde

Adapted from literature for the detection of formaldehyde in reactions with purified Pt2OGD-1. A 50 µL aliquot of was withdrawn from the reaction and mixed with purpald® reagent stock (50 µL, 34 mM purpald® reagent in 2 M aq. NaOH, prepared freshly) in a new 1.5 mL microcentrifuge tube. For calibration, formaldehyde standard solutions in reaction buffer (1 mM, 500 µM, 100 µM; 50 µL each) and plain reaction buffer as blank were mixed with equal volumes of purpald® reagent. The samples were incubated without shaking for 1 h at 21 °C during which the purple color developed. After that, a solution of sodium periodate (50 µL of a 33 mM stock in 0.2 M aq. NaOH) and water (50 µL) was added, and the sample was centrifuged for 5 min at 14k × g. 100 µL of the supernatant was transferred to a clear PS-microtiter plate and the absorption at 550 nm was measured on a Molecular Devices SpectraMax plate reader. Path-check was enabled using reaction buffer + 1.5% v/v DMSO as cuvette reference. The extinction coefficient was calculated to ε_{550nm} = 6938.8 $M^{-1}cm^{-1}$.

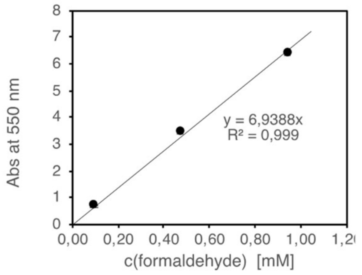

Figure S12. Calibration curve in the concentration range of 0.1 – 1 mM formaldehyde based on the absorption at 550 nm.

5.6. Computational Details

All calculations were performed using the B3LYP-D3(BJ)[16] functional as implemented in Gaussian16 software, revision C.01.[17] Geometries were optimized using the LANL2DZ basis set for Fe and the 6-31G(d,p) basis set for the other atoms. At the same level of theory, frequency calculations were carried out to calculate zero-point energies. The effect of protein surrounding was estimated using single-point calculations with the implicit SMD solvation method (ε=4) with the same basis set combination.[18] The energies were further refined by single-point calculations on the optimized structures using the LANL2DZ basis set for Fe and the 6-311+G(2d,2p) basis set for all other atoms. Final energies were thus obtained considering the larger basis set energies, the solvation effect, and the zero-point correction. The calculations involving iron were carried out in the high-spin state.

The active site model is shown in Figure S13 and consists of the iron(IV)-oxo, two imidazoles and two acetates.

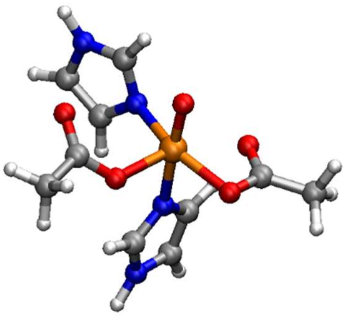

Figure S13. Iron complex used in the calculations as a simple model of the Pt2OGD-1 active site.

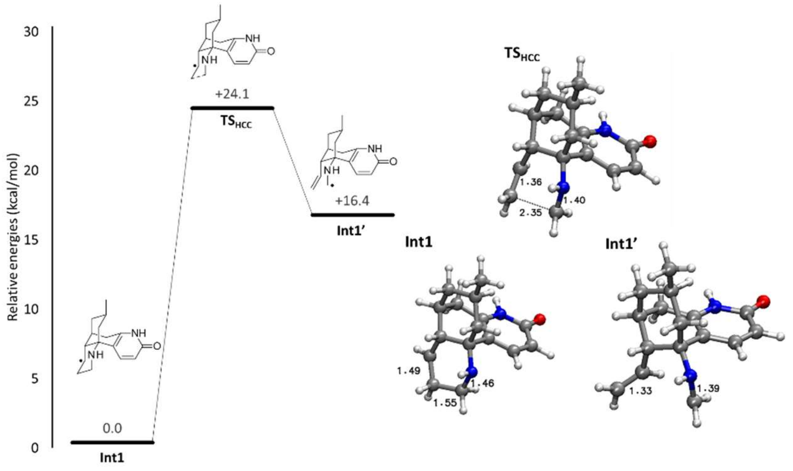

Figure S14. Calculated energy profile and relative optimized structures for the homolytic cleavage of C9-C10 bond.

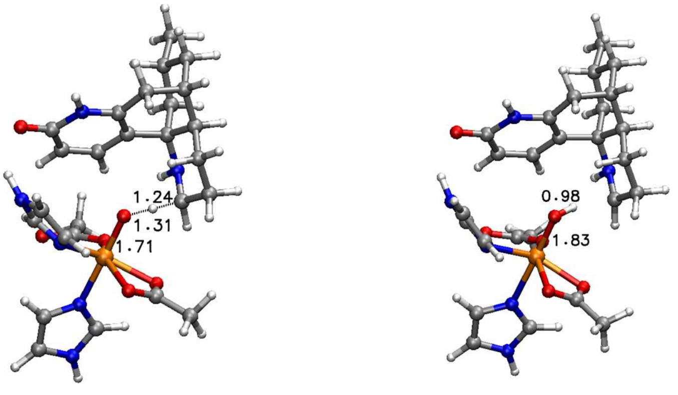

TS-C9$_{HT}$ (9.4) **Fe^{III}-OH-Int1-C9 (-7.0)**

Figure S15. Optimized geometries of transition state and intermediate of C9 hydrogen abstraction. Energy (in kcal/mol) is given relative to **Fe^{IV}=O-11**.

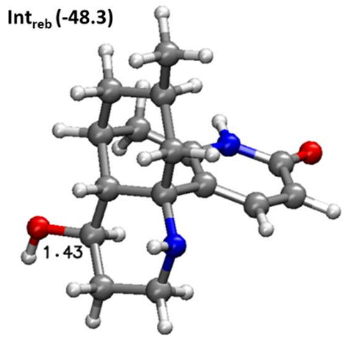

Figure S16. Optimized geometry of rebound intermediate. Energy (in kcal/mol) is given relative to **Int1** and **Fe^{III}-OH**.

Table S3. Calculated absolute energies (in a.u.). "BS1"= LANL2DZ for Fe and 6-31G(d,p) for other atoms. "BS2"= LANL2DZ for Fe and 6-311+G(2d,2p) for other atoms. E_{tot} = E_{BS2} + ZPE_{BS1} + ($E_{solv,BS1}$ - E_{BS1}).

Species	E_{BS1}	E_{BS2}	ZPE_{BS1}	$E_{solv,BS1}$	E_{tot}
**11**	-807.837318	-808.047192	0.363305	-807.846224	-807.692792
**Fe^{IV}=O**	-1108.210196	-1108.531528	0.253936	-1108.229092	-1108.296488
**Fe^{IV}=O-11**	-1916.079260	-1916.619869	0.619536	-1916.096616	-1916.017689
**TS_{HT}**	-1916.050538	-1916.594165	0.610783	-1916.070013	-1916.002858
**Fe^{III}-OH-Int1**	-1916.062260	-1916.607492	0.613518	-1916.083887	-1916.015601
**TS-$C9_{HT}$**	-1916.048873	-1916.589971	0.6123658	-1916.074026	-1916.002758
**Fe^{III}-OH-Int1-C9**	-1916.069745	-1916.616128	0.6140189	-1916.096487	-1916.028850
**Fe^{III}-OH**	-1108.866107	-1109.194561	0.262189	-1108.888595	-1108.954860
**Fe^{II}-OH**	-1108.957261	-1109.296266	0.261090	-1109.028513	-1109.106427
**Int2**	-806.963598	-807.167327	0.350697	-807.030492	-806.883524
**TS_{retro}**	-806.942841	-807.146028	0.349205	-807.012600	-806.866581
**Int3**	-806.950767	-807.156790	0.348832	-807.019933	-806.877124
**Int1**	-807.169331	-807.380241	0.348204	-807.178280	-807.040986
**TS_{HCC}**	-807.126232	-807.341282	0.345264	-807.132837	-807.002623
**Int1′**	-807.137708	-807.353959	0.344994	-807.143563	-807.014819
**Int1**	-807.169331	-807.380241	0.348204	-807.178280	-807.040986
**Int_{reb}**	-883.054704	-883.295898	0.367606	-883.068232	-882.941820
**FeII**	-1033.062177	-1033.356059	0.248828	-1033.085942	-1033.130996

6. Supplementary Results

6.1. Detection of formaldehyde as coupled product in headspace GC-MS assays

In a headspace GC-vial (20 mL, Agilent) lyophilized *E. coli* cells containing Pt2OGD-1 (10.0 mg, 32 mU) and Pt2OGD-3 (5.0 mg, 46 mU) were suspended in Na-K phosphate/NaCl buffer (50/100 mM, pH 7.5; 840 µL). A combined stock of 2-oxoglutaric acid and ascorbic acid (100 mM each, pH adjusted to 7.5 with 10 M aq. NaOH; 150 µL, 15 mM final conc., 15 equiv.) was added to the cell suspension. For the biotransformation, a stock of substrate **10** in DMSO (100 mM, 10 µL, 1.0 mM final conc., 1.0 equiv.) was added. The vial was immediately sealed with a Teflon®-coated septum and a crimp cap and incubated for 2 h on top of an Eppendorf Thermomixer that was set to 30 °C and 450 rpm mixing speed. Afterwards, the vial content was derivatized and analyzed by headspace GC-MS as described in section 5.4.

In parallel, a second biotransformation was performed under identical conditions. From this duplicate reaction, a sample (100 µL) was withdrawn after 2 h, diluted with MeCN (100 µL, containing 0.1% v/v formic acid), incubated at 21 °C for 5 min and centrifuged (3 min, 20,000 × g) before transfer to a plastic HPLC vial. The reaction composition was then analyzed with HPLC-DAD using *Method B* (see section 5.2).

In addition to the biotransformation ("BT"), control samples (i) without substrate (DMSO was used instead) ("SB"), (ii) without biocatalyst and substrate ("SCB") and (iii) spiked with formaldehyde (540 µM) ("SP") were incubated (30 °C, 2 h), derivatized (60 °C, 1 h) and analyzed in the same way as the biotransformation sample. All experiments were performed in triplicate. Concentrations were calculated based on the calibration in section 5.4.

Background- and recovery correction was performed as follows:

$$[FA]_{corr\_b\_r} = \frac{[FA]_{bt\_meas} - [FA]_{bl}}{r}$$

(Formula 1)

whereas

$$r = \frac{\left([FA]_{sp\_meas} - [FA]_{bl}\right)}{[FA]_{sp}}$$

(Formula 2)

$[FA]_{corr\_b\_r}$ .... Corrected formaldehyde concentration in biotransformation sample in µmol/L.

$[FA]_{bt\_meas}$ .... Measured formaldehyde concentration in biotransformation sample in µmol/L (corresponds to sample "BT" above).

$[FA]_{bl}$ .... Measured formaldehyde concentration in substrate-blank in µmol/L (corresponds to sample "SB" above).

r .... Recovery.

$[FA]_{sp\_meas}$ .... Measured formaldehyde concentration in spiked sample in µmol/L (corresponds to sample "SP" above).

$[FA]_{sp}$ .... Actual concentration of formaldehyde contained in spiked sample in µmol/L.

Table S4. Results of the headspace GC-MS formaldehyde measurements and comparison to HPLC-conversion data. Indicated errors correspond to reactions performed in triplicates.

**Parameter**	**Value**
$[FA]_{sp\_meas}$	377±3 µM
$[FA]_{bl}$	177±11 µM
$[FA]_{sp}$	540 µM
r	37%
$[FA]_{bt\_meas}$	375±38 µM
$[FA]_{corr\_b\_r}$	534±38 µM
[**14**]$_{HPLC}$[a]	280±9 µM

[a]Measured concentration with HPLC-DAD according to *Method B*.

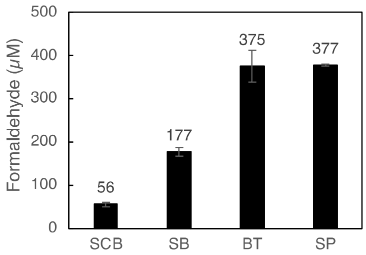

Figure S17. Detection of formaldehyde in the headspace of Pt2OGD-1 biotransformations. SCB: control reaction ("blank") without substrate and cell preparation, SB: control reaction with cell preparation but without substrate, BT: biotransformation containing both substrate and cell preparation, SP: biotransformation without substrate spiked with 540 µM formaldehyde. Error bars correspond to triplicate experiments.

6.2. Detection of formaldehyde as coupled product with a Purpald® assay.

Nine portions (50 µL) of purified Pt2OGD-1 (1.42 mg) in storage buffer (50 mM Na/K-Pi, 100 mM NaCl, 10% w/w glycerol, 50 mM dithiothreitol) were thawed on ice. All reagents were mixed according to the pipetting scheme in **Table S5** and performed in triplicates. The positive control contained 1.42 mg Pt2OGD-1, 450 µM substrate **11** and 1.5 mM 2-oxoglutarate.

Table S5. Pipetting scheme for detecting formaldehyde in biotransformations with a purpald assay.

Order Of Addition	Component	Positive Control	Neg. Control 1 (No Enzyme)	Neg. Control 2 (No Substrate)	Neg. Control 3 (No Co-Substrate)
1	Reaction buffer (Na/K-Pi 50 mM, NaCl 100 mM, pH 7.8)	46.4 µL	46.4 µL	46.4 µL	47.9 µL
2	Substrate **11** (21 mM stock in DMSO)	2.14 µL	2.14 µL	–	2.14 µL
3	DMSO	–	–	2.14 µL	–
4	Storage buffer	–	50 µL	–	–
5	Enzyme solution in storage buffer (28.4 µg/µL)	50 µL	–	50 µL	50 µL
6	Co-substrate stock (100 mM 2-oxoglutarate, 100 mM ascorbate in reaction buffer pH 7.8)	1.5 µL	1.5 µL	1.5 µL	–

The reaction was then incubated for 1 h at 22 °C in an Eppendorf Thermomixer. Then samples were withdrawn and processed as indicated in section 5.5 above for formaldehyde detection. Results are shown in **Figure S12**. Significantly higher levels of formaldehyde were detected in the positive control sample compared to the control reactions.

Immediately after withdrawing the sample aliquot for the purpald® assay, the remaining sample (50 µL) was mixed with MeOH (50 µL) and incubated at 21 °C for 5 min, followed by centrifugation (14k × g) for 5 min. The supernatant was transferred to a PP-HPLC-vial and conversion of **11** to **14** is determined with HPLC-DAD according to section 5.2. Results are shown in **Figure S12c**. After background correction (subtracting the signal from negative control 3), almost equimolar amounts of formaldehyde and **14** were detected in the positive control (448 vs. 562 µM).

6.3. Time study

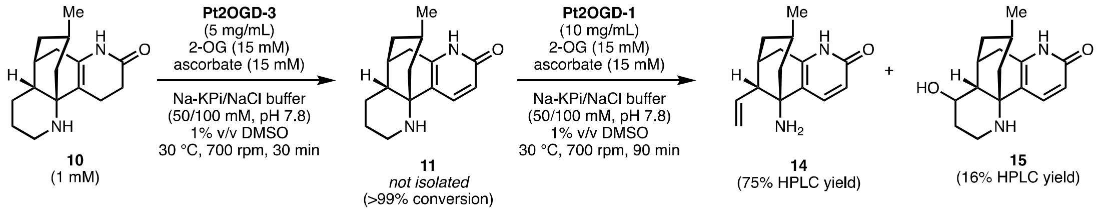

Scheme S1. One-pot, two-step cascade with Pt2OGD-3 and Pt2OGD-1 to convert **10** into **14** and **15** via **11**.

In four 2.0 mL microcentrifuge tubes, lyophilized *E. coli* whole cells containing Pt2OGD-3 (5.0 mg each, 114 mU) were provided and rehydrated in reaction buffer (sodium-potassium phosphate/NaCl, 50/100 mM, pH 7.8, 840 µL) for 5 min in a thermoshaker (30 °C, 700 rpm). A combined stock of 2-oxoglutaric acid and ascorbic acid (100 mM each in reaction buffer, pH adjusted to 7.8 with 10 M aq. NaOH; 150 µL, 15 mM final conc., 15 equiv.) was added to the cell suspension. The reaction was started by addition of substrate **10** (10 µL of a 100 mM stock in hot DMSO). All four vials were closed and incubated in a thermoshaker (30 °C, 700 rpm, horizontally).

Samples (100 µL) were withdrawn simultaneously from two of the four vials after 1, 5, 15, 25 and 30 min. One of the samples was quenched with MeOH (100 µL) and incubated at 21 °C for 30 min, followed by centrifugation (20,000 × g, 5 min) and transfer to PP-HPLC vials for HPLC analysis.

In two separate 2.0 mL microcentrifuge tubes, lyophilized *E. coli* whole cells containing Pt2OGD-1 (10.0 mg each, 18.6 mU) were provided. After 30 min, the Pt2OGD-1 biocatalyst preparation was added to the two running reactions which were not sampled so far. Again, samples were withdrawn simultaneously from these two vials 5, 10, 20, 30, 60 and 90 min after addition of Pt2OGD-1. Samples were processed for analysis as described above.

Samples for HPLC were analyzed using *Method B* described in section 5.2 to determine the reaction composition at individual time points.

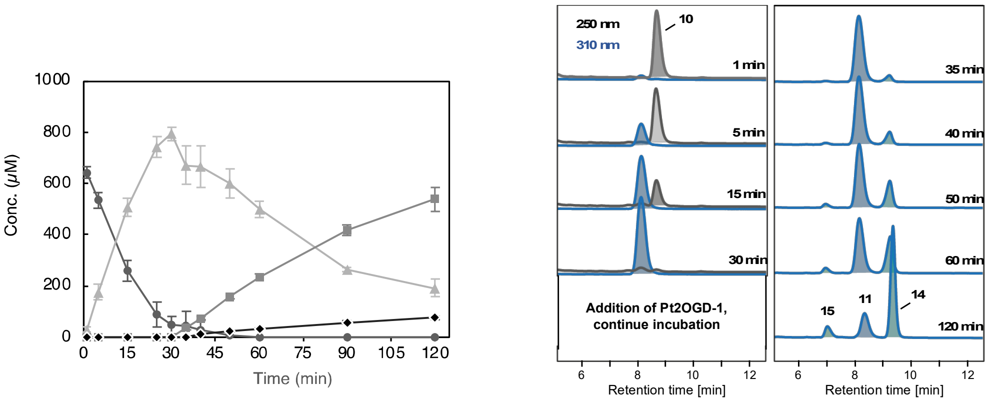

Figure S18. Time profile of a two-step, one-pot cascade with Pt2OGD-3 and Pt2OGD-1 transforming **10** into **14** via **11**. Legend: ● **10** (N-desmethyl-α-obscurine); ▲ **11** (N-desmethyl-β-obscurine); ■ **14** (casuarinine H); ◆ **15** (lycosquarrine M). Error bars refer to triplicate experiments.

6.4. 2-Oxoglutarate as limiting component

In a 1.5 mL microcentrifuge tube Ni-affinity purified Pt2OGD-1 (33 µL of a 28.4 µg/µL solution in storage buffer, 937 µg) was mixed with **11** (100 µL of a 1.0 mM stock solution in Na-KPi buffer 50 mM, pH 7.8, 1.0 nmol, 1.0 equiv.). A combined stock of 2-oxoglutarate and ascorbate (10 µL, 10 mM each in reaction buffer, pH re-adjusted to 7.8 with 10 M aq. KOH, 1 nmol., 1.0 equiv.) was added to start the reaction. The final mixture thus contained purified Pt2OGD-1 (162 µM, 6.6 µg/µL), 700 µM **11**, 700 µM 2-oxoglutarate and 700 µM ascorbate in 143 µL reaction volume. The vial was incubated horizontally at 21 °C for 5 h. After that, an aliquot (100 µL) was withdrawn and mixed with MeOH (100 µL). The experiment was carried out in triplicates. A conversion of 97±1% of **11** to **14** and **15** was detected, indicating that only one equiv. of 2-OG is needed for complete conversion.

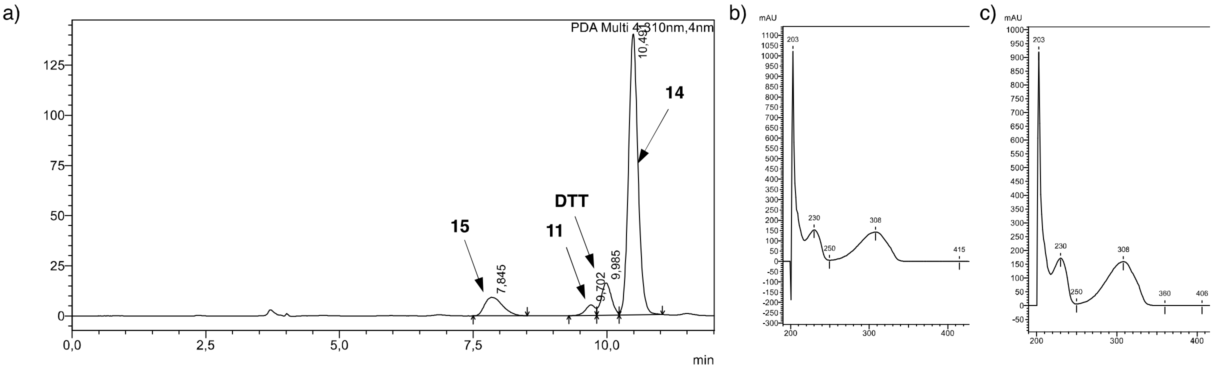

Figure S19. Chromatogram of the Pt2OGD-1 biotransformation with one equivalent 2-oxoglutarate reaching 97% of conversion (a). The UV-Vis spectrum of the peak corresponding to **14** in the biotransformation **(b)** and a reference spectrum of a standard of **14** (c) are identical. Quantification is based on calibration curves (Figure S10).

6.5. Reductive interception of reaction intermediates

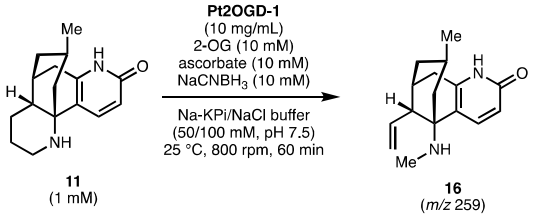

Scheme S2. Reductive trapping of the formiminium intermediate to yield N-methyl-casuarinine H.

Lyophilized *E. coli* cells containing recombinant Pt2OGD-1 (2 × 10.0 mg, 18.6 mU) were provided in 2.0 mL microcentrifuge tubes. *N*-desmethyl-β-obscurine (**11**, 1.1 mg) was completely dissolved in reaction buffer (50 mM Na-KPi, 100 mM NaCl, pH 7.5; 4.0 mL; 1.1 mM substrate conc.) in an ultrasonic bath. The substrate solution was added to the cell preparations (450 µL each) followed by a freshly prepared, combined stock solution of 2-ketoglutarate and ascorbate in reaction buffer (50 µL of a 100 mM stock, pH was re-adjusted to 7.5 with 10 M NaOH before addition; 10 mM final conc; 10 equiv. each). To one tube (!) a solution of sodium cyanoborohydride (50 µL of 100 mM stock in reaction buffer) was added immediately after the co-substrate. Both tubes were incubated in a thermoshaker (25 °C, 800 rpm, tube oriented horizontally) for 60 min. After this time, a sample aliquot (100 µL) was withdrawn and quenched by mixing with MeCN (100 µL). The samples were centrifuged (5 min at 21k × g) and filtered through a 0.22 µM syringe filter before HPLC-MS measurements using *Methods A and C* (see section 5.1). For the identification of **16** by its fragmentation pattern see **Table S9**.

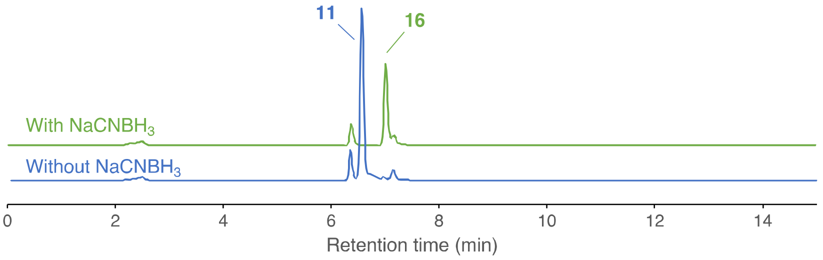

Figure S20. Extracted HPLC-MS traces (*m/z* 259) for the biotransformation of **11** catalyzed by Pt2OGD-1 in the presence of sodium cyanoborohydride (green) and without reducing agent (blue).

6.6. Temperature study

The reaction catalyzed by Pt2OGD-1 (conversion of **11** to **14** and **15**) was performed at different temperatures between 10 and 40 °C (conditions as in 6.4, without reducing agent). The highest conversion (78%) is observed at 20 °C with a steep drop of activity beyond 25 °C. This activity drop could be due to thermal deactivation of Pt2OGD-1. The relative amount of **15** (*m/z* 275, MH^+) and casuarinine H (**14**, *m/z* 245, MH^+) increases with higher temperatures, which translates to a smaller energy difference between their transition states.

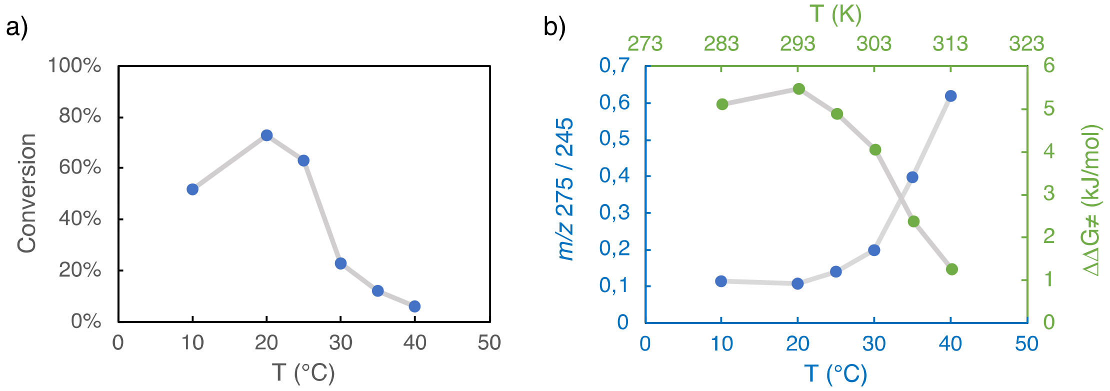

Figure S21. a) Temperature dependence of the conversion of **11** to **14**/**15** catalyzed by Pt2OGD-1. b) Temperature dependence of the ratio of **14** and **15** (blue), translated into energy barrier differences (green).

6.7. MS/MS fragmentation analysis of biotransformation products

For chromatograms and mass spectra see section 9.

Table S6. Proposed fragmentation mechanism of **11** (t_R 5.03 min, *m/z* 259.1799).

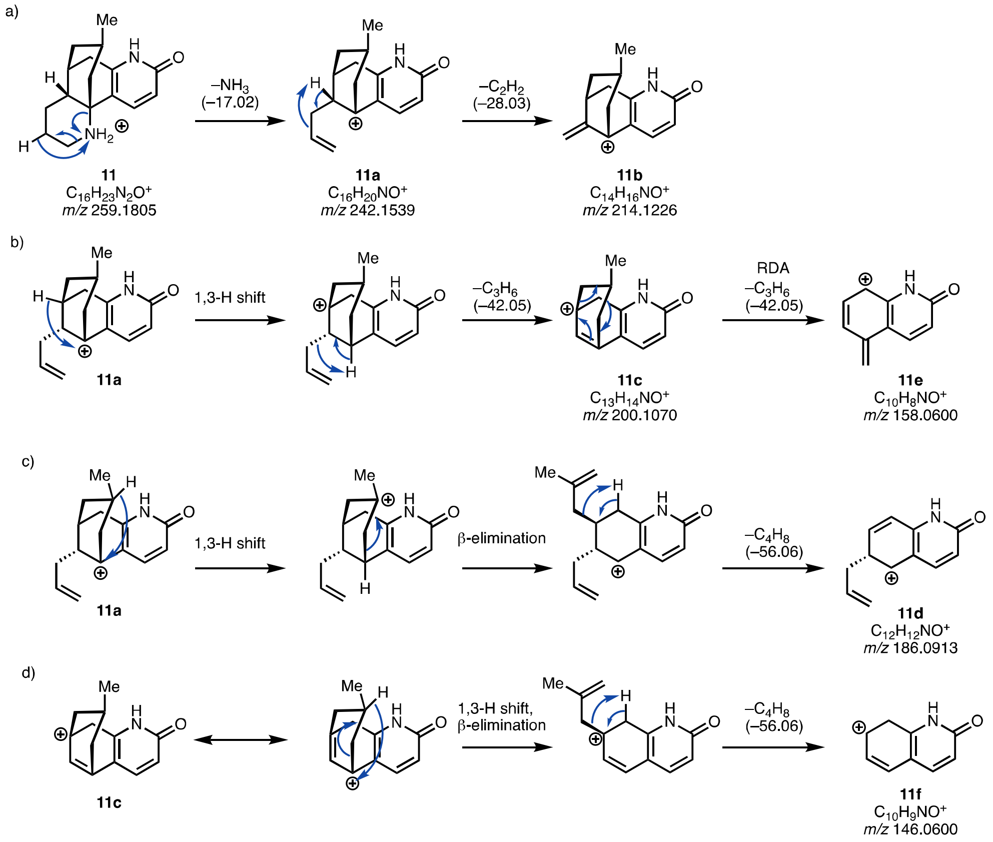

Experimental MS/MS (*m/z*)	Relative abundance (%)	Calculated *m/z*	Elemental composition (M+H⁺)	Error (ppm)	Identification
259.1803	100	259.1805	$C_{16}H_{23}N_2O^+$	-0.62	NDMBO (**11**)
242.1537	49	242.1539	$C_{16}H_{20}NO^+$	-0.80	**11a**
214.1225	4	214.1226	$C_{14}H_{16}NO^+$	-0.67	**11b**
200.1069	7	200.1070	$C_{13}H_{14}NO^+$	-0.51	**11c**
186.0913	7	186.0913	$C_{12}H_{12}NO^+$	-0.17	**11d**
158.0599	2	158.0600	$C_{10}H_8NO^+$	-1.14	**11e**
146.0599	7	146.0600	$C_{10}H_9NO^+$	-0.82	**11f**

Table S7. Proposed fragmentation mechanism of **15** (t_R 4.04 min, *m/z* 275.1747)

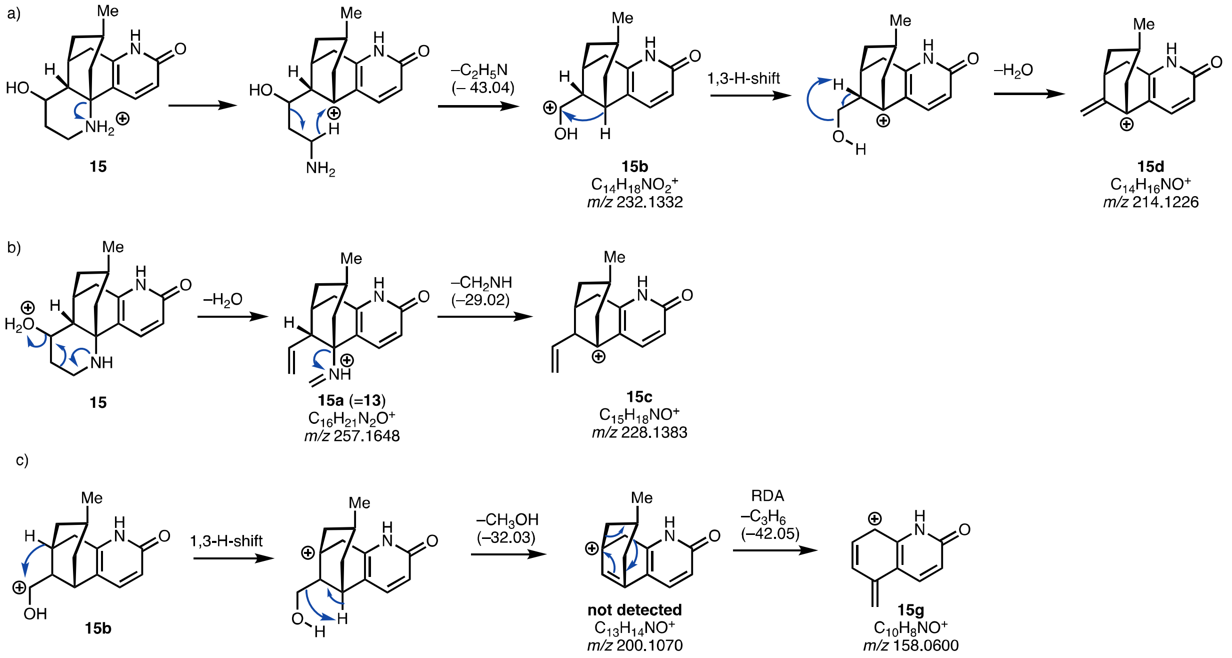

Experimental MS/MS (*m/z*)	Relative abundance (%)	Calculated *m/z*	Elemental composition	Error (ppm)[a]	Identification
275.1752	100	275.1754	$C_{16}H_{23}O_2N_2^+$	-0.85	Lycosquarrine M (**15**)
257.1649	4	257.1648	$C_{16}H_{21}N_2O^+$	0.18	**15a**
232.1329	68	232.1332	$C_{14}H_{18}NO_2^+$	-1.50	**15b**
228.1375	5	228.1383	$C_{15}H_{18}NO^+$	-3.68	**15c**
214.1223	11	214.1226	$C_{14}H_{16}NO^+$	-1.41	**15d**
202.1232	6	202.1226	$C_{13}H_{16}NO^+$	-2.82	Not assigned
188.1075	3	188.1070	$C_{12}H_{14}NO^+$	2.71	Not assigned
158.0599	7	158.0600	$C_{10}H_8NO^+$	-1.14	**15g**
146.0599	2	146.0600	$C_{10}H_9NO^+$	-0.83	**15h** (acc. to **11f**)

Table S8. Proposed fragmentation mechanism of **14**.

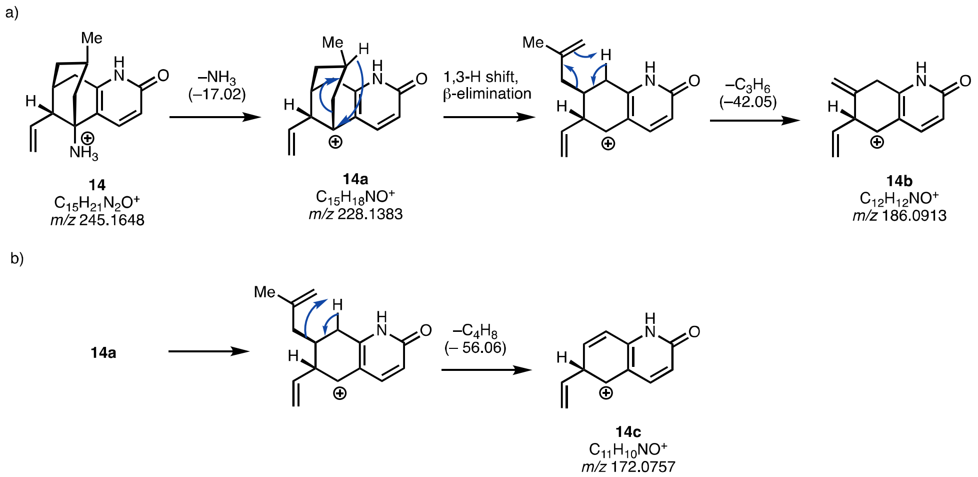

Experimental MS/MS (m/z)	Relative abundance (%)	Calculated m/z	Elemental composition	Error (ppm)[a]	Identification
245.1646	100	245.1648	$C_{15}H_{21}N_2O^+$	-0.93	Casuarinine H (**14**)
228.1382	52	228.1383	$C_{15}H_{18}NO^+$	-0.27	**14a**
186.0912	7	186.0913	$C_{12}H_{12}NO^+$	-0.75	**14b**
172.0755	6	172.0757	$C_{11}H_{10}NO^+$	-0.04	**14c**
158.0598	5	158.0600	$C_{10}H_8NO^+$	-1.45	**14d** (acc. to **11e**)
146.0600	5	146.0600	$C_{10}H_9NO^+$	-0.29	**14e** (acc. to **11f**)

Table S9. Proposed fragmentation mechanism of **16**.

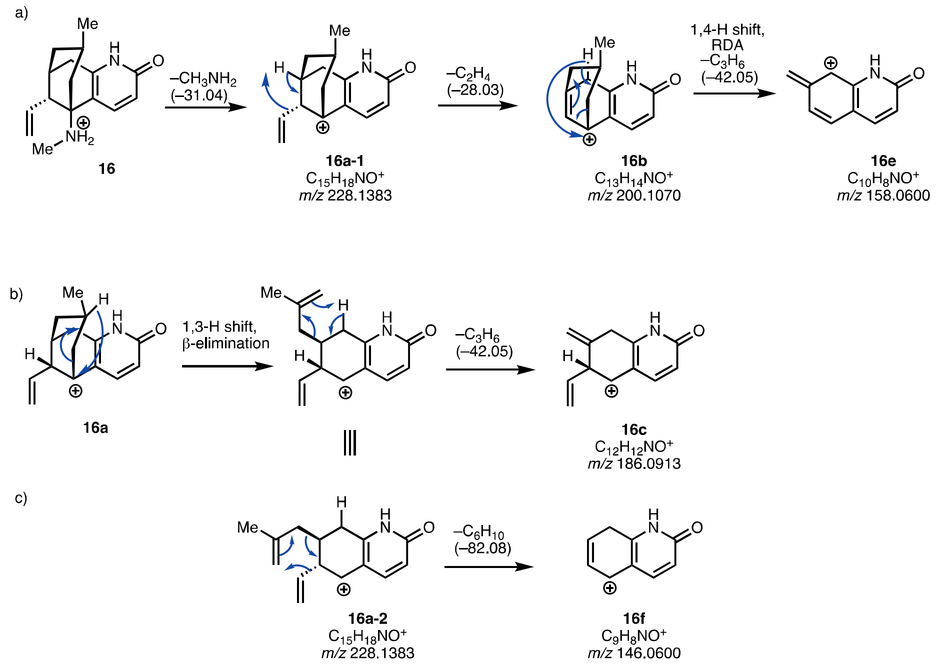

Experimental MS/MS (*m/z*)	Relative abundance (%)	Calculated *m/z*	Elemental composition	Error (ppm)[a]	Identification
259.1805	15	259.1805	$C_{16}H_{23}ON_2^+$	-0.03	N-Methyl-casuarinine H (**16**)
228.1381	100	228.1383	$C_{15}H_{18}NO^+$	-0.62	**16a-1, 16a-2**
200.1071	6	200.1070	$C_{13}H_{14}NO^+$	0.20	**16b**
186.0911	8	186.0913	$C_{12}H_{12}NO^+$	-1.35	**16c**
172.0756	13	172.0757	$C_{11}H_{10}NO^+$	-1.45	**16d** (acc. to **14c**)
158.0598	8	158.0600	$C_{10}H_8NO^+$	-0.45	**16e**
146.0602	10	146.0600	$C_9H_8NO^+$	-0.34	**16f**

Table S10. Comparison of detectable mass ion fragments.

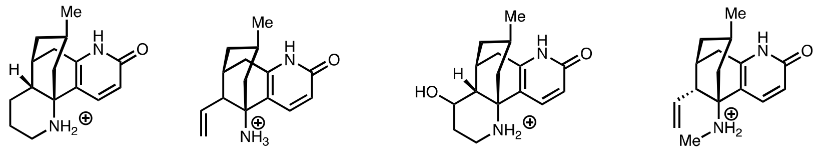

Fragment	**11**	**14**	**15**	**16**	Elemental composition
1	–	–	275.1752 (**15**)	–	$C_{16}H_{23}N_2O_2^+$
2	259.1803 (**11**)	–	–	259.1805 (**16**)	$C_{16}H_{23}N_2O^+$
3	–	–	257.1649 (**15a**)	–	$C_{16}H_{21}N_2O^+$
4	–	245.1646 (**14**)	–	–	$C_{15}H_{21}N_2O^+$
5	242.1537 (**11a**)	–	–	–	$C_{16}H_{20}NO^+$
6	–	–	232.1329 (**15b**)	–	$C_{14}H_{18}NO_2^+$
7	–	228.1382 (**14a**)	228.1375 (**15c**)	228.1381 (**16a-1,-2**)	$C_{15}H_{18}NO^+$
8	214.1225 (**11b**)	–	214.1223 (**15d)**	–	$C_{14}H_{16}NO^+$
9	–	–	202.1232 (not assigned**)**	–	$C_{13}H_{16}NO^+$
10	200.1069 (**11c**)	–	–	200.1071 (**16b**)	$C_{13}H_{14}NO^+$
11	–	–	188.1075 (not assigned)	–	$C_{12}H_{14}NO^+$
12	186.0913 (**11d**)	186.0912 (**14b**)	–	186.0911 (**16c**)	$C_{12}H_{12}NO^+$
13	–	172.0755 (**14c**)	–	172.0756 (**16d**)	$C_{11}H_{10}NO^+$
14	158.0599 (**11e**)	158.0598 (**14d**)	158.0599 (**15g**)	158.0598 (**16e**)	$C_{10}H_8NO^+$
15	146.0599 (**11f)**	146.0600 (**14e**)	146.0599 (**15h**)	146.0602 (**16f**)	$C_{10}H_9NO^+$

7. Related Alkaloid Natural Products

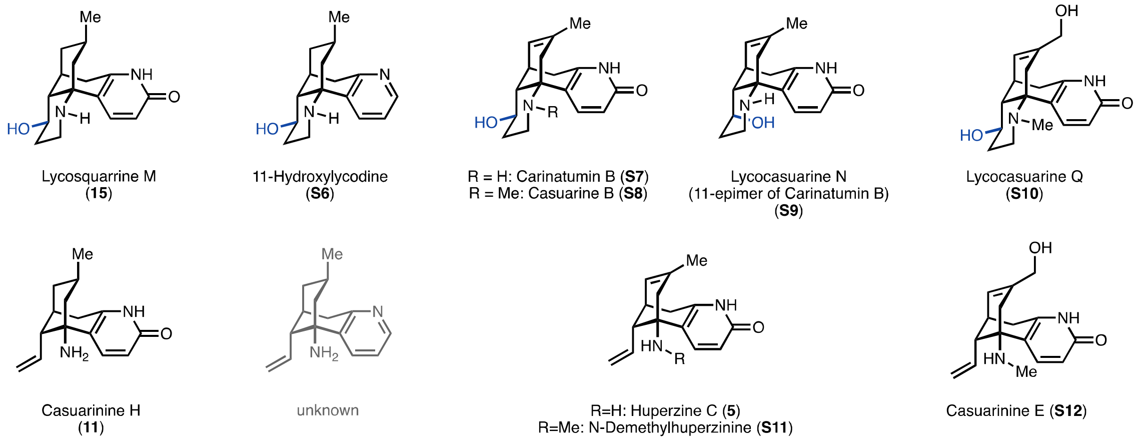

Figure S22. Alkaloids from *Lycopodiaceae* plants featuring a hydroxylation at C11 and their ring-cleaved counterparts.

8. Preparative Procedures

8.1. Synthesis of *N*-desmethyl-α-obscurine (10)

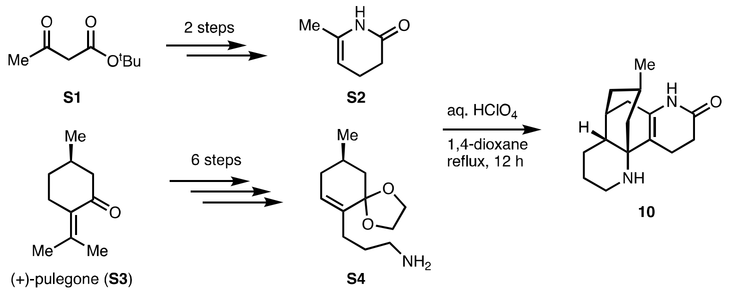

Scheme S3. Convergent route for the preparation of *N*-desmethyl-α-obscurine (**10**) according to literature procedures.

The synthesis of **10** was carried out according to literature procedures.[19][20] The obtained brown crude material was ultimately purified by washing with ice-cold acetonitrile to yield **10** as yellowish solid (103 mg, 37% from 228 mg of **S4**). **^{1}H NMR** (300 MHz, $CDCl_3$) δ_H [ppm] 7.78 (bs, 1H), 2.80 (d, *J* = 12.6 Hz, 1H), 2.51–2.10 (m, 6H), 1.88 (bs, 1H), 1.73–1.56 (m, 5H), 1.52–1.34 (m, 5H), 1.28–1.13 (m, 2H), 0.84 (d, *J* = 6.2 Hz, 3H). **LC-MS** (API-ES+) *m/z* (%) = 261.1 [$(M+H)^+$](100), 262.1 (18.1), 263.1 (1.7).
Analytical data in agreement with literature.[19,20,21]

8.2. Synthesis of *N*-desmethyl-β-obscurine (11)

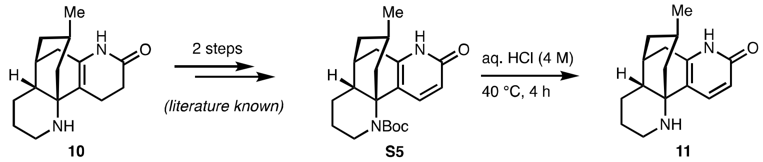

Scheme S4. Preparation of **11** from Boc-protected **10**.

N-Boc protected β-obscurine (**S5**, 25.1 mg, 70.0 μmol, 1.0 equiv.; prepared from crude **11** over two steps according to literature protocols[19]) was reconstituted in HCl aq. (4 mol/L, 500 μL) in a 4.0 mL glass vial with stir bar. The vial was heated to 40 °C (water bath) and the content was stirred for 2 h. A second portion of HCl aq. (100 μL) was added, and stirring was continued until a clear yellowish solution was obtained (ca. 2 h). The acidic reaction mixture was washed with CH_2Cl_2 (2 × 500 μL; phase separation by centrifugation at 14,000 × g for 1 min). The vial was then placed in ice-water and aq. NaOH solution (4 mol/L, 700 μL) was added dropwise to the stirred solution. The cloudy, basic emulsion (pH > 12) was extracted with CH_2Cl_2 (4 × 500 μL), the combined extracts were dried over Mg_2SO_4, and the solvent was removed in a stream of air to yield the title compound **3** as beige crystalline solid (12.4 mg, 68%).
^{1}H NMR (300 MHz, $CDCl_3$) δ_H [ppm] 7.59 (d, *J* = 9.3 Hz, 1H), 6.45 (d, *J* = 9.3 Hz, 1H), 2.95 (dd, *J* = 18.7, 7.0 Hz, 1H), 2.76 (bd, *J* = 13.1 Hz, 1H), 2.45 (d, *J* = 18.6 Hz, 1H), 2.50–2.35 (m, 1H), 2.01 (m, 1H), 1.72 (bd, *J* = 13.8 Hz, 1H), 1.62–1.35 (m, 6H), 1.33–1.19 (m, 2H), 1.03 (dd, $J_1 = J_2$ = 11.6 Hz, 1H), 0.80 (d, *J* = 6.0 Hz, 3H). **^{13}C NMR** (75 MHz,

CDCl3) δ_C [ppm] 165.2, 145.0, 140.1, 118.2, 117.4, 54.6, 49.8, 44.8, 43.3, 41.6, 33.4, 30.0, 28.0, 26.0 (2C), 22.0. **LC-MS** (API-ES+) *m/z* = 259 [(M+H)+] (100), 260.2 (18). **HR-MS** found *m/z* 259.1799, calculated *m/z* 259.1805: $C_{16}H_{23}N_2O$ [M+H]+, Δ 0.62 ppm.
Analytical data in agreement with literature.[21]

8.3. Isolation of casuarinine H (14) and lycosquarrine M (15) from a preparative biotransformation

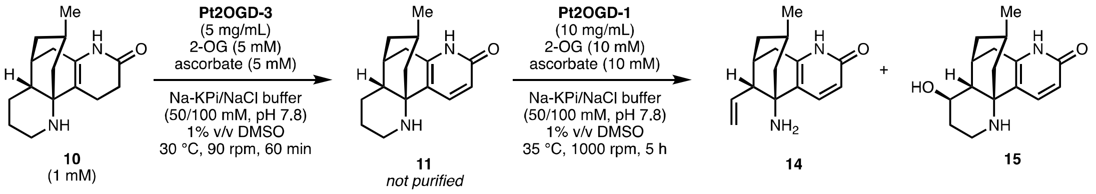

Scheme S5. Preparation of **14** and **15** via a two-step preparative scale biotransformation.

N-desmethyl-α-obscurine (**10**, 100.0 mg, 0.38 mmol, 1.0 eq, 1.0 mM final conc.) was dissolved in reaction buffer (360 mL, Na-KPi 50 mM, NaCl 100 mM, pH 7.8) in a 500 mL glass bottle by ultrasonication (5 min, 23 °C). Lyophilized cell preparation containing Pt2OGD-3 (1.90 g, 5 mg/mL, 35.15 U) was added in small portions to the solution and the suspension was shaken gently by hand at room temperature for 5 min to homogenize. A freshly prepared combined stock solution of 2-oxoglutarate and ascorbic acid (100 mM in reaction buffer, pH re-adjusted to 7.8 with 10 M aq. KOH; 19 mL, 1.9 mmol, 5.0 eq., 5 mM final conc.) was added in one portion to the biotransformation. The closed bottle was incubated horizontally in an Infors cultivation shaker at 30 °C and 90 rpm shaking speed.

A sample for HPLC was withdrawn after 60 min and analyzed according to Method B (section 5.2). Complete conversion of **10** to **11** was detected and the mixture was basified with aq. sat. Na_2CO_3 (4 mL). The basic cell suspension was shock frozen with liquid nitrogen and lyophilized for 24 h. The lyophilized mixture was then taken up in MeOH (3 × 30 mL per flask), sonicated, mixed and centrifuged at 2,576 × g for 5 min. The yellow supernatant was collected in a 500 mL flask and concentrated in vacuo at 40 °C to ca. 5 mL. The slurry containing precipitated salts was cooled on ice before passing through a pad of celite. The flask and the filter were washed with MeOH (4 × 10 mL). The yellow filtrate was concentrated again (40 °C, 5 mbar) and the process was repeated three times to remove most of the salts.

The crude product was then taken up in reaction buffer (360 mL, Na-KPi 50 mM, NaCl 100 mM, pH 7.8) in a 500 mL glass bottle and stirred until everything was dissolved. The pH of the brownish, opaque mixture was adjusted back to 7.8 with aq. HCl. Lyophilized cell preparation containing Pt2OGD-1 (3.80 g, 10 mg/mL, 14.06 U) was added in small portions to the solution and the suspension was shaken gently by hand at room temperature for 5-10 min to homogenize. The homogeneous reaction mixture was split in 1.0 mL aliquots and distributed among four 96-deepwell plates (Ritter). This step was crucial, as the biotransformation stalled out at 10-20% conversion in larger volumes. A freshly prepared combined stock solution of 2-oxoglutarate and ascorbic acid (100 mM in reaction buffer, pH readjusted to 7.8 with 10 M aq. KOH; 100 µL per well, 3.8 mmol, 10.0 eq., 10 mM final conc.) was added to each of the wells to start the reaction. The plates were then mounted

on Eppendorf shakers set to 35°C and 1000 rpm mixing speed. Samples for HPLC analysis were withdrawn after 1, 4 and 5 h from the same well and analyzed according to Method B (section 5.2). After 4 h, 77% of **14** has formed (85% conversion of **11** BRSM), and conversion did not increase between 4 and 5 h anymore. After 5 h 45 min the aliquots were recombined in 4 × 250 mL round-bottom flasks. The content of the flasks was basified by adding sat. aq. Na_2CO_3 (1 mL to each flask), shock frozen with liquid nitrogen and lyophilized for 24 h.
The dry solid was then taken up in MeOH (3 × 30 mL per flask), homogenized by sonication and centrifuged (2,576 × g, 5 min). The clear, yellow supernatant was collected in a 500 mL round-bottom flask and concentrated under reduced pressure (40 °C). Precipitating salts were removed by cooling the suspension on ice and filtration through a pad of celite, which was washed with MeOH (10 mL) after each filtration step. The concentration/filtration process was repeated three times to remove most of the salt load.
The resulting orange slurry was then taken up in ddH_2O (40 mL, pH after dissolution ca. 10) and extracted with CH_2Cl_2 (3 × 20 mL) with intermittent phase separation by centrifugation (2,576 × g, 5 min) to recover unreacted **11** and **14** in the organic phase. The aqueous phase was again extracted with 2-butanol (4 × 20 mL) to recover **15**. The extracts were concentrated under reduced pressure (40 °C) and then high vacuum to yield the two crude product fractions as orange-brown sticky oils. The crude material was dissolved in a mixture of HPLC-grade MeCN and H_2O (each containing 1%v/v diethylamine), filtered through a 0.22 μM syringe filter and subjected to purification with preparative HPLC on a Phenomenex Luna C18 (2) column (250 mm × 21.2 mm × 5 μM).
Crude **15** was purified using an isocratic flow of H_2O+1% DEA and MeCN+1%DEA in 93:3 ratio. Fractions containing pure products were combined and evaporated in a Genevac Personal Evaporator to afford **15** as colorless film (5.4 mg, 5.2% from **10**).
^{1}H NMR (700 MHz, MeOH-d4) δ_H [ppm] 7.61 (d, J = 9.3 Hz, 1H), 6.41 (d, J = 9.4 Hz, 1H), 3.35 (m, 1H), 2.96 (dd, J = 18.8, 7.0 Hz, 1H), 2.90 (br d, J = 13.2 Hz, 1H), 2.68–2.63 (m, 1H), 2.52 (t, J = 13.2 Hz, 1H), 2.39 (d, J = 19.0 Hz, 1 H), 1.90 (br d, J = 11.4 Hz, 1H), 1.83 (br d, J = 13.8 Hz, 1H), 1.60–1.52 (m, 2H), 1.49 (d, J = 10.5 Hz, 1H), 1.40–1.32 (m, 1H), 1.28–1.22 (m, 1H), 0.88 (d, J = 6.4 Hz, 3H). **^{13}C NMR** (125 MHz, MeOH-d4) δ_C [ppm] 165.2, 147.0, 140.5, 118.5 (2C), 67.3, 57.6, 51.7, 49.5, 43.5, 40.9, 36.4, 30.2, 28.6, 26.9, 22.1. **LC-MS** (API-ES+) *m/z* =275.2 [(M+H)$^+$](100) 276.2 (14.8), 277.3 (1.44). **HR-MS** found *m/z* 275.1747, calculated *m/z* 245.1754: $C_{16}H_{23}N_2O_2$ [M+H]$^+$, Δ –2.54 ppm.
Analytical data in agreement with literature.[22]

Crude **14** was purified using an isocratic flow of H_2O+1% DEA and MeCN+1%DEA in 62:38 ratio. Fractions containing pure products were combined and evaporated in a Genevac Personal Evaporator to afford **14** as beige solid (48 mg, 51% from **10**).
^{1}H NMR (300 MHz, $CDCl_3$) δ_H [ppm] 7.72 (d, J = 9.2 Hz, 1H), 6.40 (d, J = 9.5 Hz, 1H), 5.63 (ddd, J = 17.0, 10.1, 10.1 Hz, 1H), 5.23 (dd, J = 17.0, 2.2 Hz, 1H), 5.13 (dd, J = 10.2, 2.2 Hz, 1H), 3.06 (dd, J = 18.9, 7.2 Hz, 1H), 2.51 (d, J = 18.9 Hz, 1H), 2.32–2.26 (m, 1H), 2.14 (dd, J = 9.4, 3.1 Hz, 1H), 1.75 (d, J = 12.5 Hz, 1H), 1.64 (bd, J = 12.4 Hz, 1H), 1.52 – 1.22 (m, 3H), 1.22 – 1.03 (m, 2H), 0.85 (d, J = 6.2 Hz, 3H). **^{13}C NMR** (75 MHz, $CDCl_3$) δ_C [ppm] 165.0, 143.9,

140.5, 137.5, 120.6, 119.1, 117.3, 54.9, 52.3, 50.0, 42.6, 30.0, 26.5, 21.9. **LC-MS** (API-ES+) *m/z* = 245.2 [$(M+H)^+$] (100), 228.1 (24), 246.2 (17), 229.2 (4). **HR-MS** found *m/z* 245.1642, calculated *m/z* 245.1648: $C_{15}H_{21}N_2O$ $[M+H]^+$, Δ –2.61 ppm.

Analytical data in agreement with literature.[19]

9. NMR and MS Spectra of Compounds

- ^{1}H NMR spectrum of *N-desmethyl*-α-obscurine (**10**) (CDCl3, 300 MHz).
- Mass spectrum [LC/API-ES(+)] of *N-desmethyl*-α-obscurine (**10**).
- ^{1}H NMR spectrum of *N-desmethyl*-β-obscurine (**11**) (CDCl3, 300 MHz).
- ^{13}C NMR spectrum of *N-desmethyl*-β-obscurine (**11**) (CDCl3, 75 MHz).
- Mass spectrum [LC/API-ES(+)] of *N-desmethyl*-β-obscurine (**11**).
- Chromatogram of reference sample containing *N*-desmethyl-β-obscurine (**11**)
- Full HR-MS spectrum (*m/z* 100-700) of *N*-desmethyl-β-obscurine (**11**).
- MS/MS spectrum (stepped NCE 18, 30, 45) of **11** (*m/z* 259.1977).
- ^{1}H NMR spectrum of casuarinine H (**14**) (CDCl3, 300 MHz).
- ^{13}C NMR spectrum of casuarinine H (**14**) (CDCl3, 75 MHz).
- Mass spectrum [LC/API-ES(+)] of casuarinine H (**14**).
- Chromatogram of reference sample containing casuarinine H (**14**) (500 µM, 0.5 µL)
- Full HR-MS spectrum (*m/z* 100-700) of casuarinine H (**14**)
- MS/MS spectrum (stepped NCE 18, 30, 45) of **14** (*m/z* 245.1642).
- ^{1}H NMR spectrum of lycosquarrine M (**15**) (MeOH-d4, 700 MHz).
- ^{13}C-NMR of 11-epi-lycosquarrine M (**15**) (MeOH-d4, 175 MHz).
- ^{1}H-^{1}H COSY of lycosquarrine M (**15**) (MeOH-d4, 700 MHz).
- ^{1}H-^{13}C HSQC of lycosquarrine M (**15**) (MeOH-d4).
- ^{1}H-^{13}C HMBC of lycosquarrine M (**15**) (MeOH-d4).
- ROESY of lycosquarrine M (**15**) (MeOH-d4).
- Zoom into the ROESY spectrum of lycosquarrine M (**15**) (MeOH-d4). Correlations to the proton on C11 are annotated.
- Observed NMR correlations in lycosquarrine M (**15**).
- Comparison of ^{1}H- and ^{13}C NMR spectroscopic data for lycosquarrine M (**15**) with literature data.
- Mass spectrum [LC/API-ES(+)] of lycosquarrine M (**15**).
- Full HR-MS spectrum (*m/z* 100-700) of lycosquarrine M (**15**).
- MS/MS spectrum (stepped NCE 18, 30, 45) of **15** (*m/z* 275.1747).
- Chromatogram of Pt2OGD-1 biotransformation sample containing lycosquarrine M (**15**) and casuarinine H (**14**).
- Chromatogram of Pt2OGD-1 biotransformation sample in the presence of $NaCNBH_3$ containing *N*-methyl-casuarinine H (**16**) and casuarinine H (**14**).
- Full HR-MS spectrum (*m/z* 100–700) of *N*-methyl-casuarinine H (**16**).
- MS/MS spectrum (stepped NCE 18, 30, 45) of **16** *m/z* 259.1800.
- Headspace chromatograms and extracted mass spectrum of a derivatized formaldehyde standard.
- Headspace chromatograms and extracted mass spectrum of a derivatized biotransformation sample.

- Example chromatograms for the chromatographic separation and detection of **10, 11** and **14** (standard samples) using *Method B.*
- Example chromatograms for the chromatographic separation and mass-selective detection of **10, 11, 14** and **15** in a typical biotransformation sample using *Method A.*

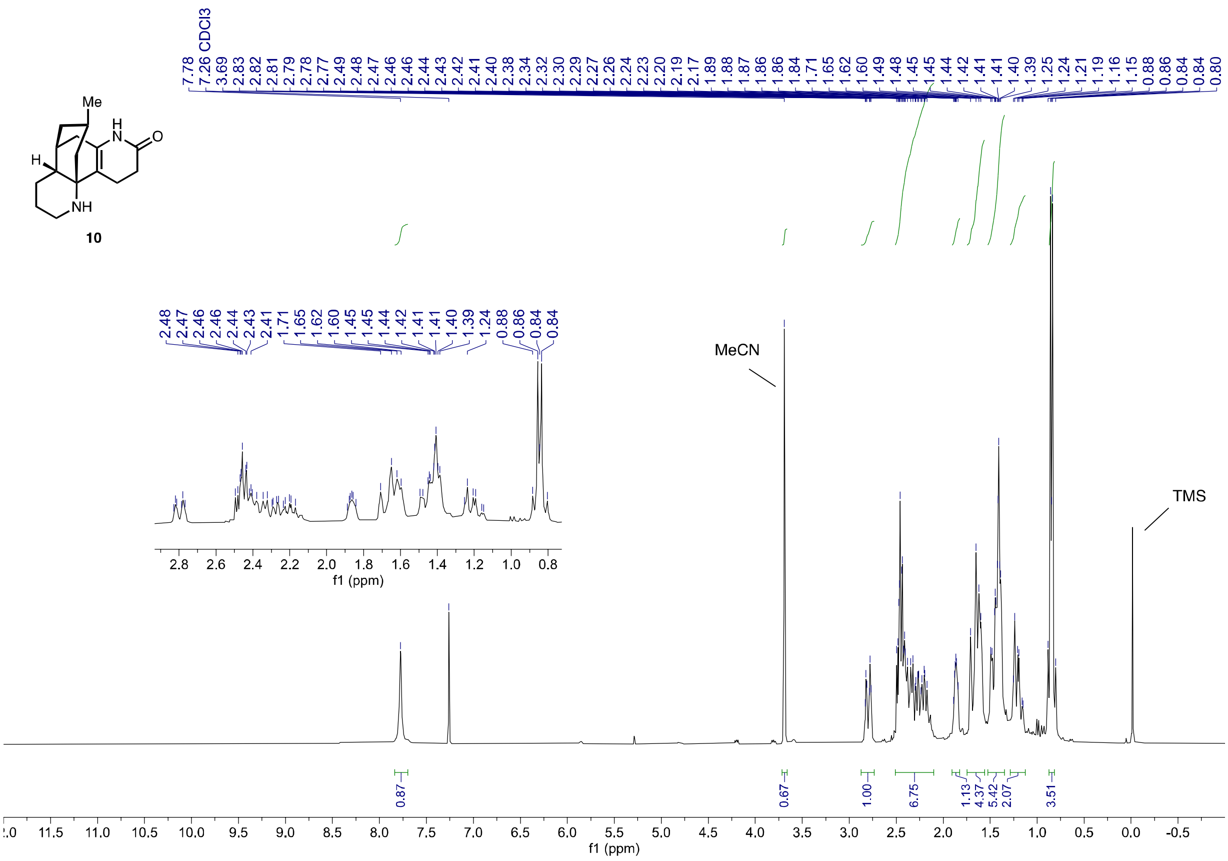

Figure S23. ^{1}H NMR spectrum of *N*-desmethyl-α-obscurine (**10**) ($CDCl_3$, 300 MHz).

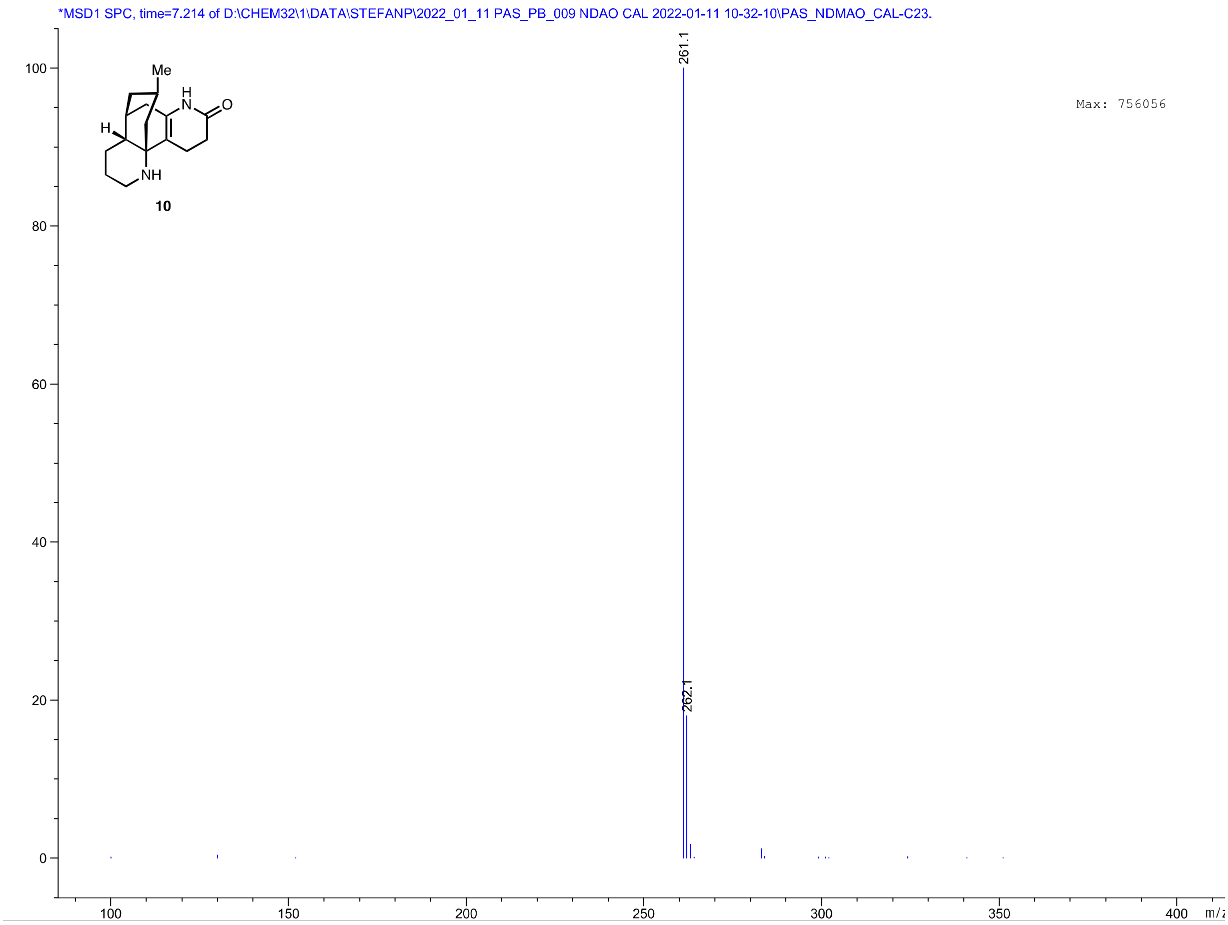

Figure S24. Mass spectrum [LC/API-ES(+)] of *N*-desmethyl-α-obscurine (**10**).

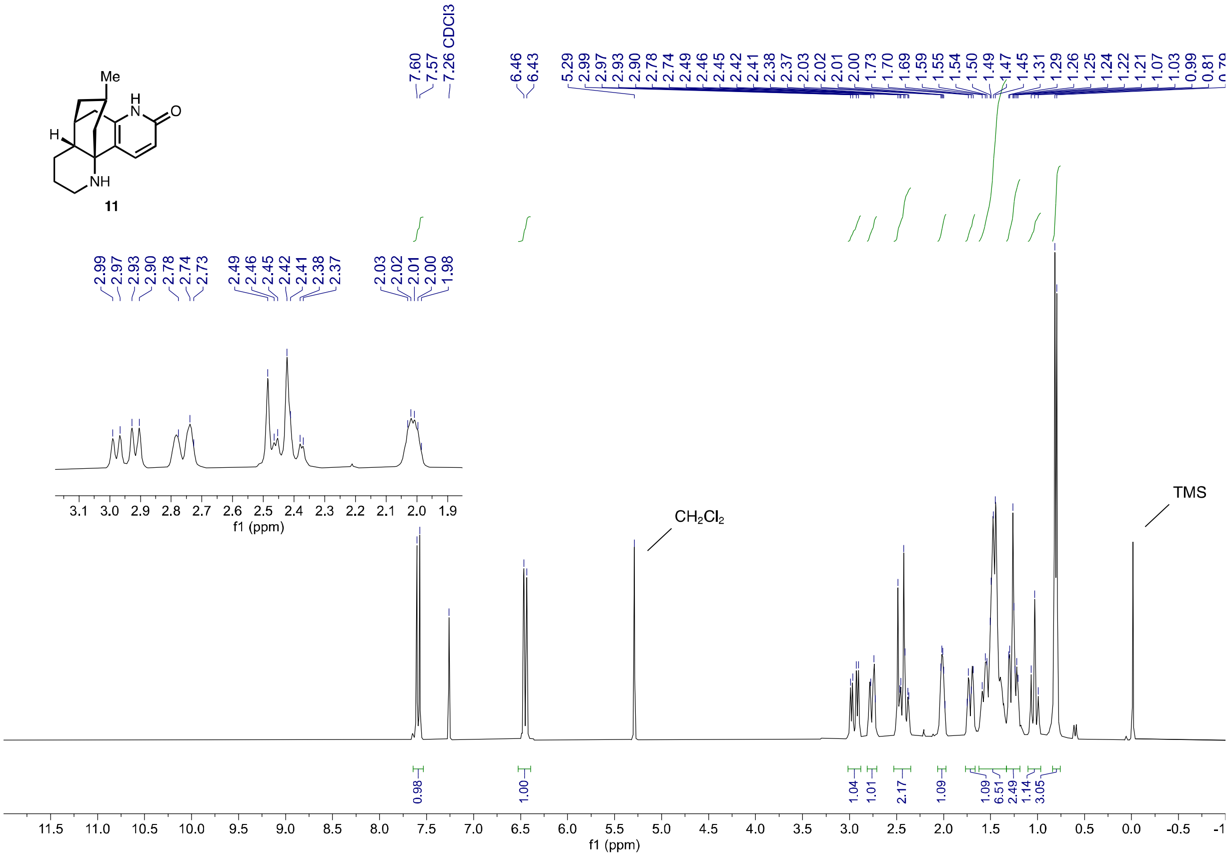

Figure S25. ^{1}H NMR spectrum of *N*-desmethyl-β-obscurine (**11**) ($CDCl_3$, 300 MHz).

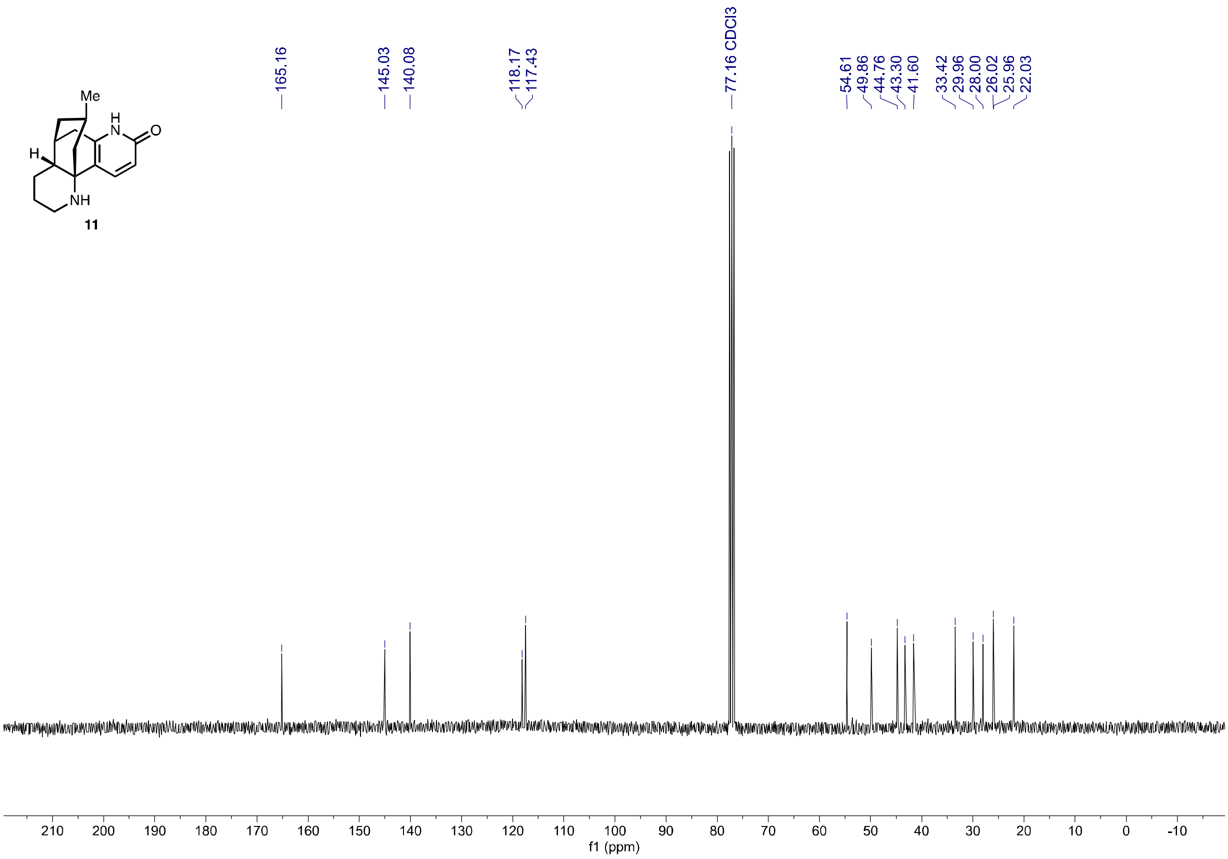

Figure S26. ^{13}C NMR spectrum of *N*-desmethyl-β-obscurine (**11**) ($CDCl_3$, 75 MHz).

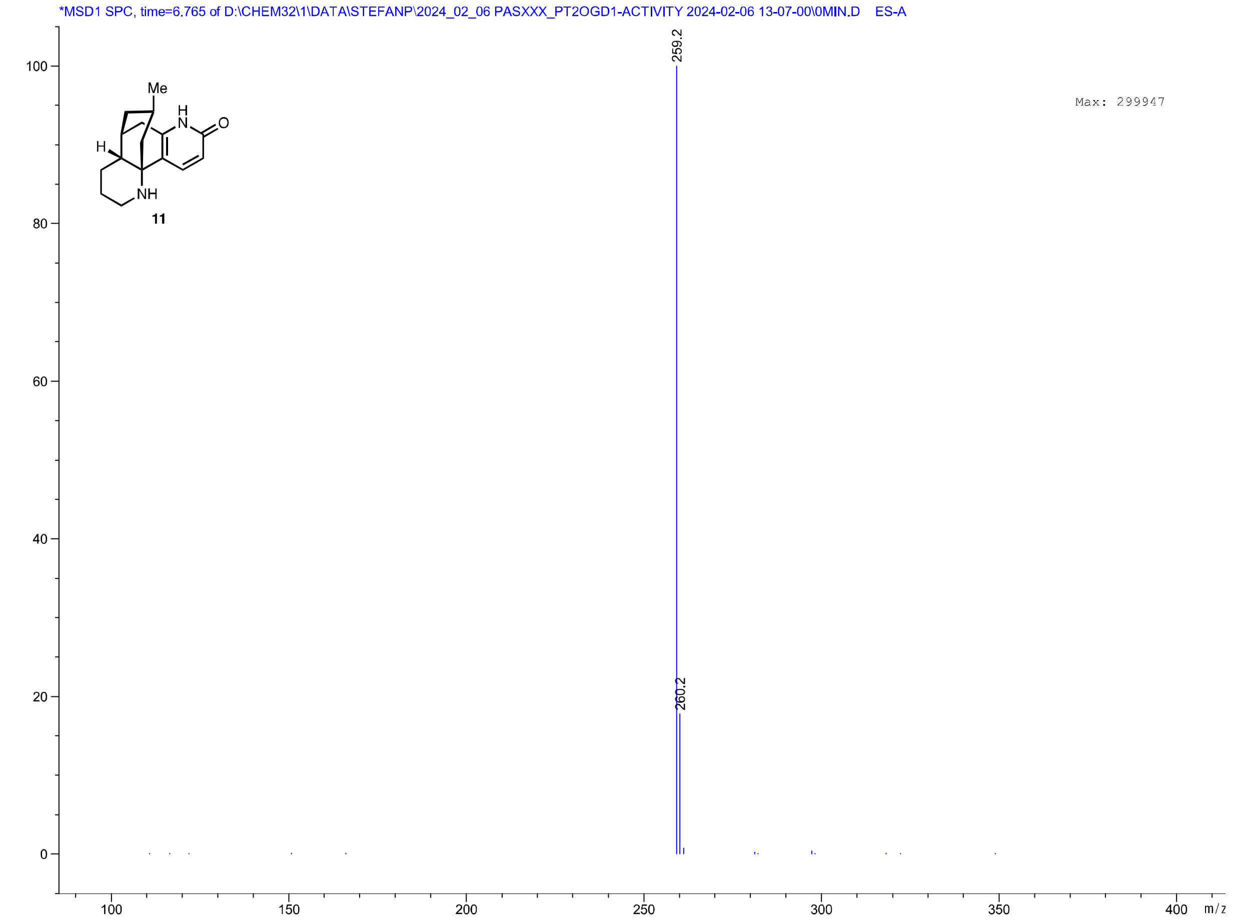

Figure S27. Mass spectrum [LC/API-ES(+)] of *N*-desmethyl-β-obscurine (**11**).

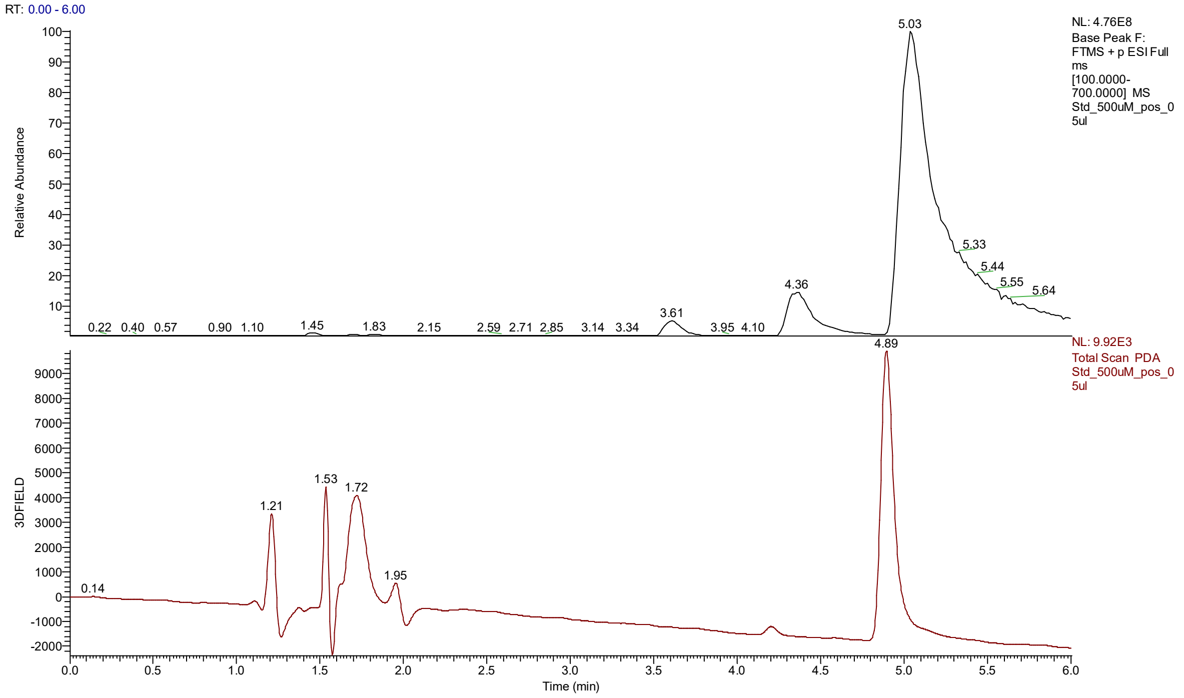

Figure S28. Chromatogram of reference sample containing *N*-desmethyl-β-obscurine (**11**) (500 µM, 0.5 µL); upper trace, HESI full MS, *m/z* 100-700; lower trace, DAD total scan, 210-450 nm).

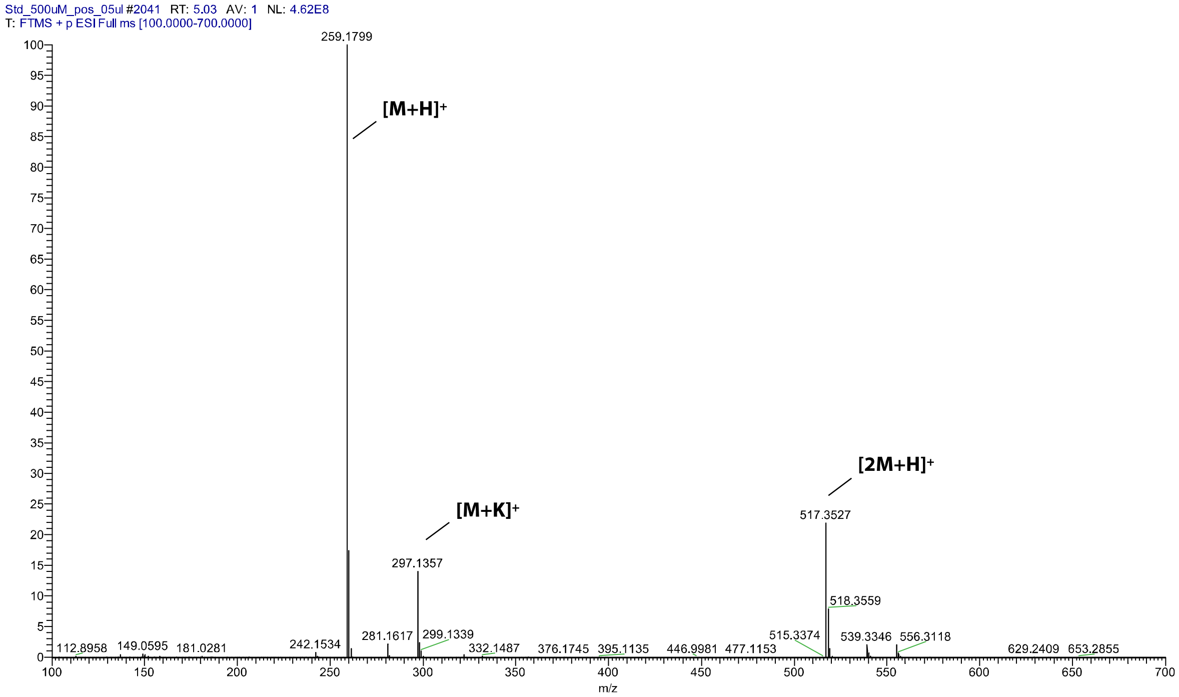

Figure S29. Full MS spectrum (*m/z* 100-700) of *N*-desmethyl-β-obscurine (**11**) (t_R = 5.03 min, *m/z* 259.1799). $C_{16}H_{23}N_2O$ $[M+H]^+$, Δ 0.62 ppm

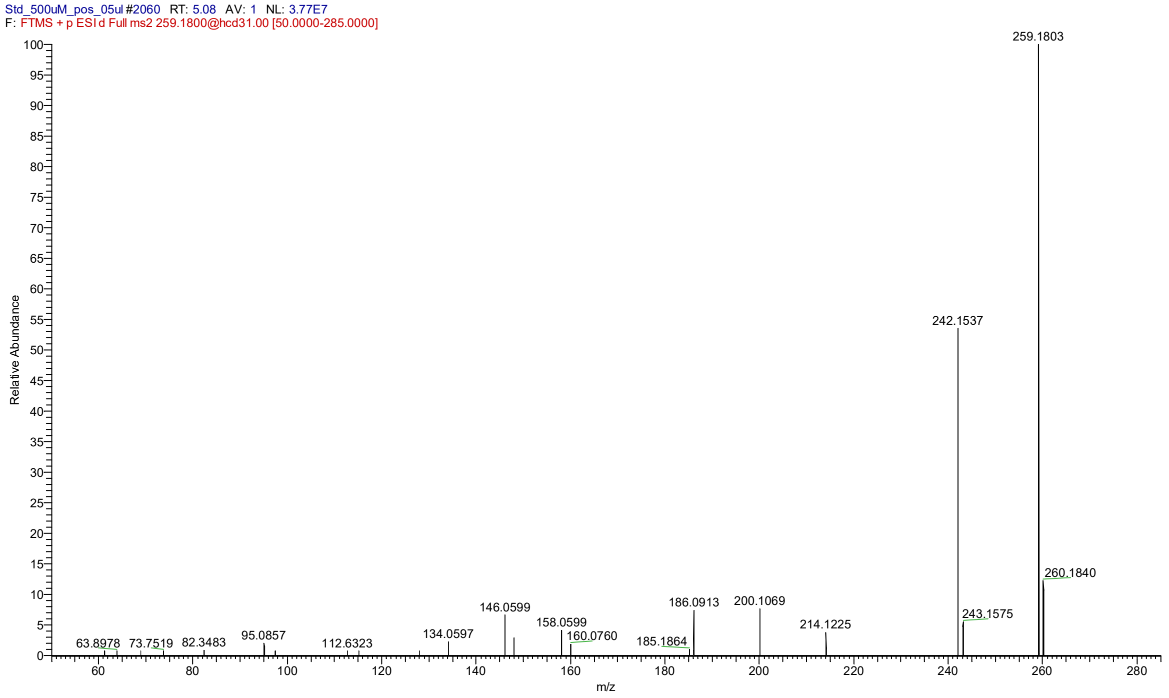

Figure S30. MS/MS spectrum (stepped NCE 18, 30, 45) of **11** (*m/z* 259.1977).

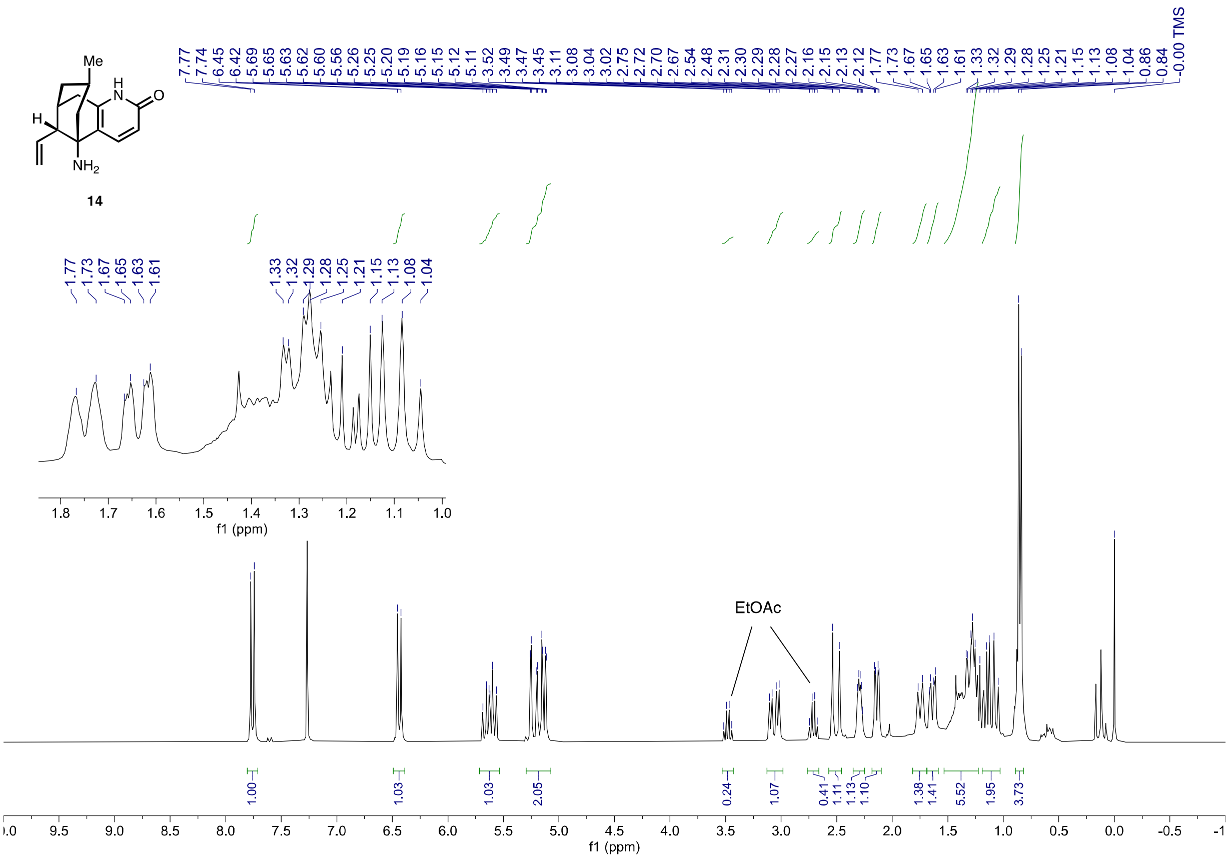

Figure S31. ^{1}H NMR spectrum of casuarinine H (**14**) ($CDCl_3$, 300 MHz).

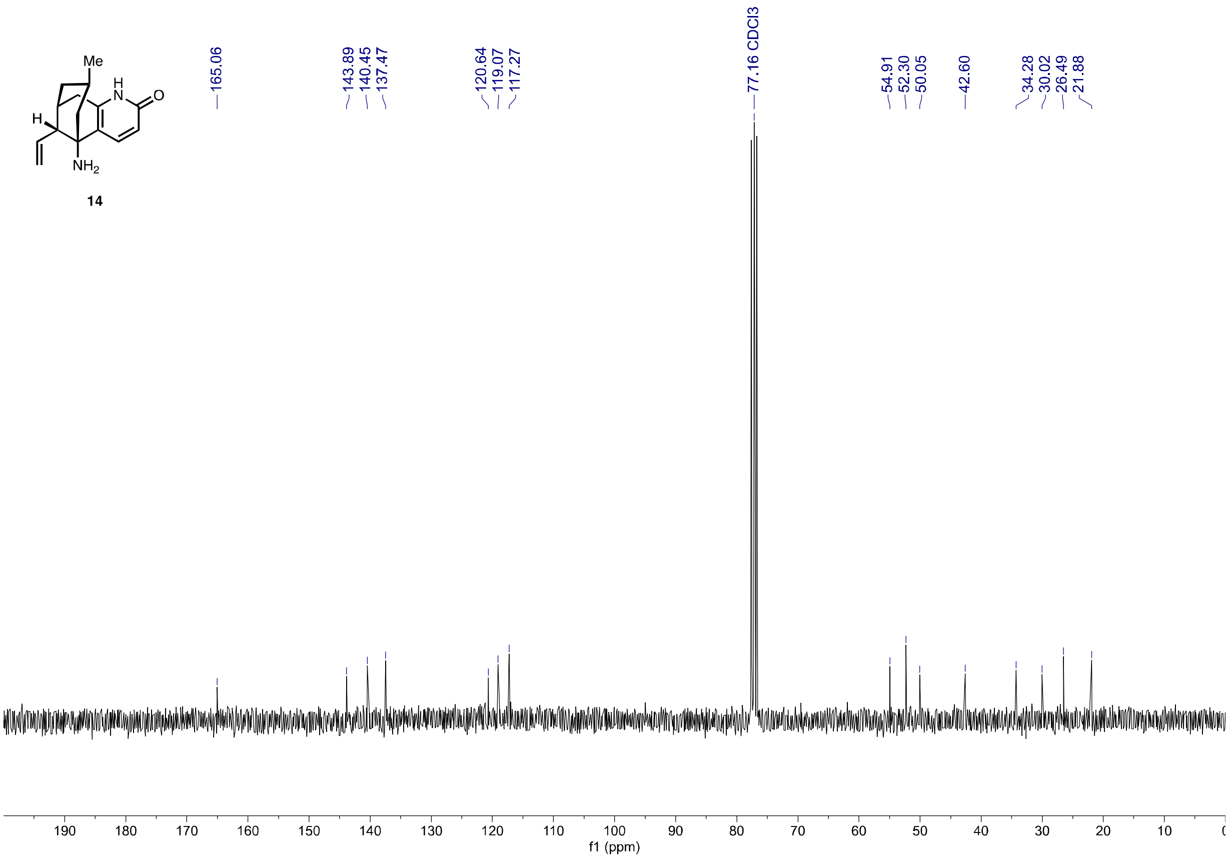

Figure S32. ^{13}C NMR spectrum of casuarinine H (**14**) ($CDCl_3$, 75 MHz).

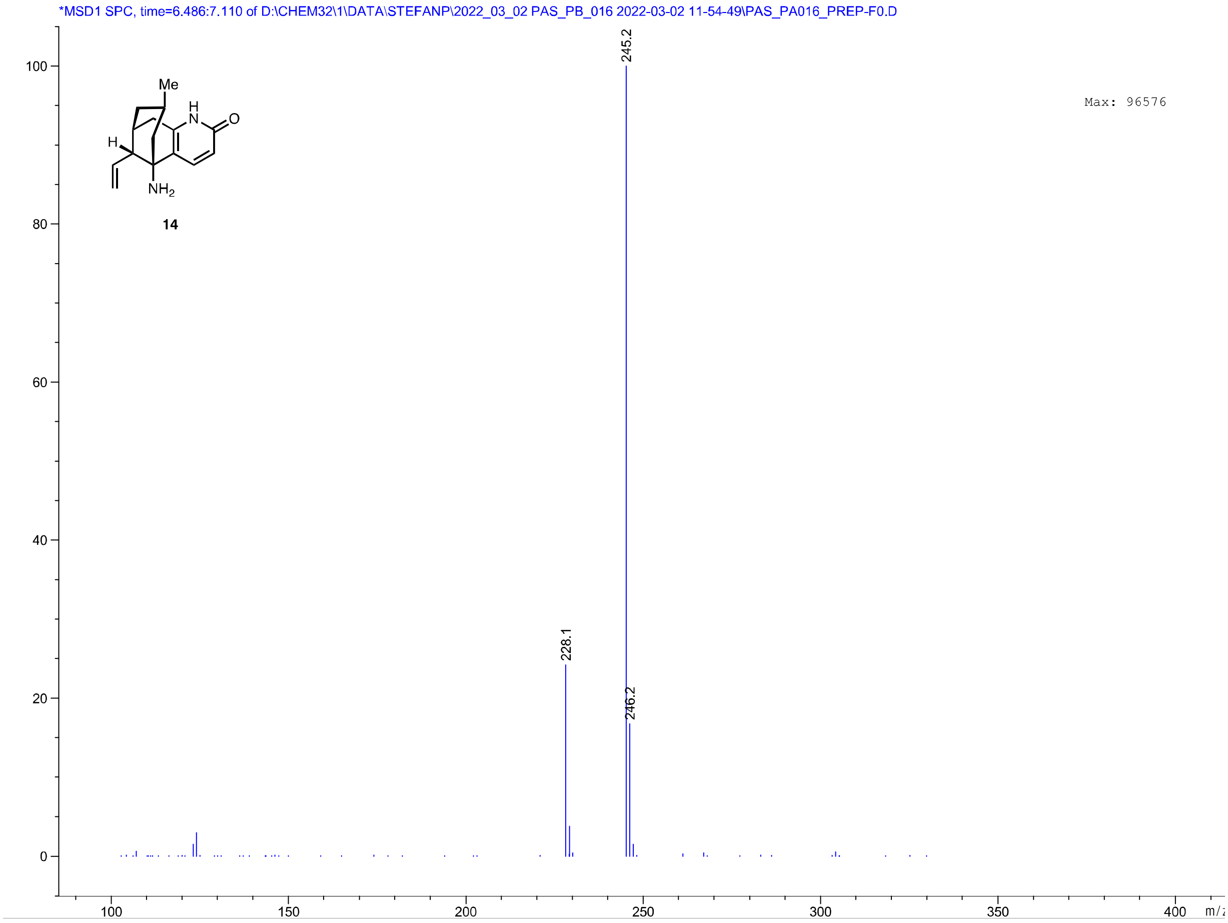

Figure S33. Mass spectrum [LC/API-ES(+)] of casuarinine H (**14**).

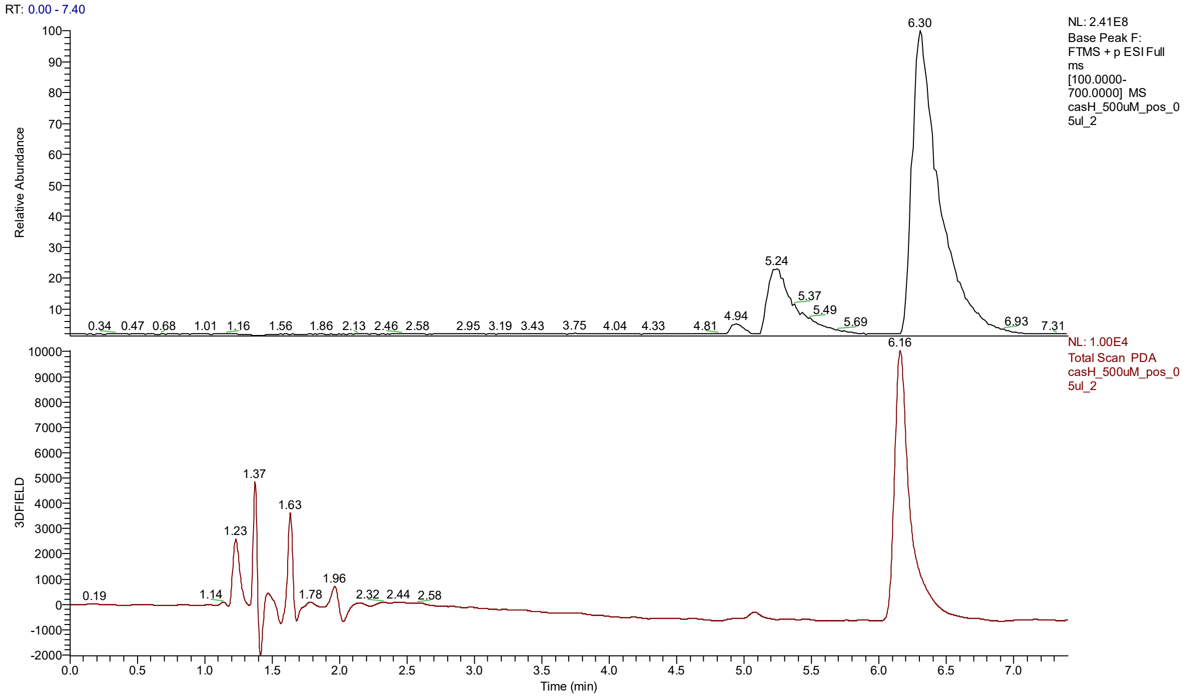

Figure S34. Chromatogram of reference sample containing casuarinine H (**14**) (500 µM, 0.5 µL); upper trace, HESI full MS, *m/z* 100-700; lower trace, DAD total scan, 210-450 nm).

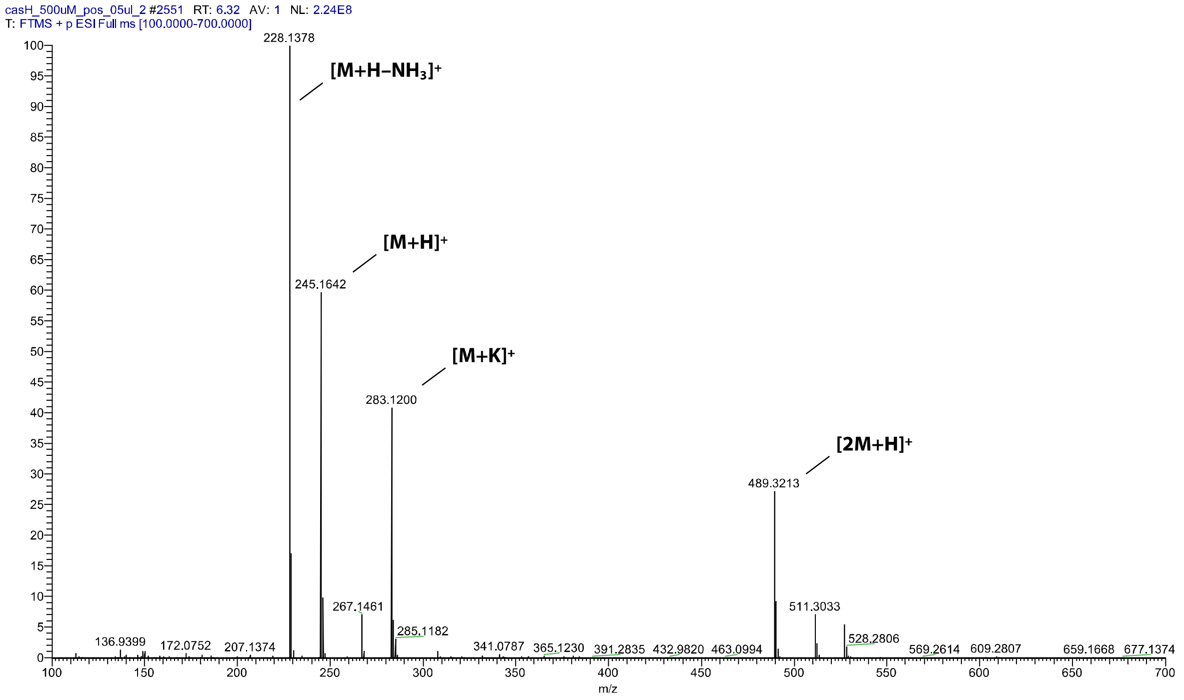

Figure S35. Full MS spectrum (*m/z* 100-700) of casuarinine H (**14**) t_R = 6.32 min, *m/z* 245.1642: $C_{15}H_{21}N_2O$ $[M+H]^+$, Δ –2.61 ppm.

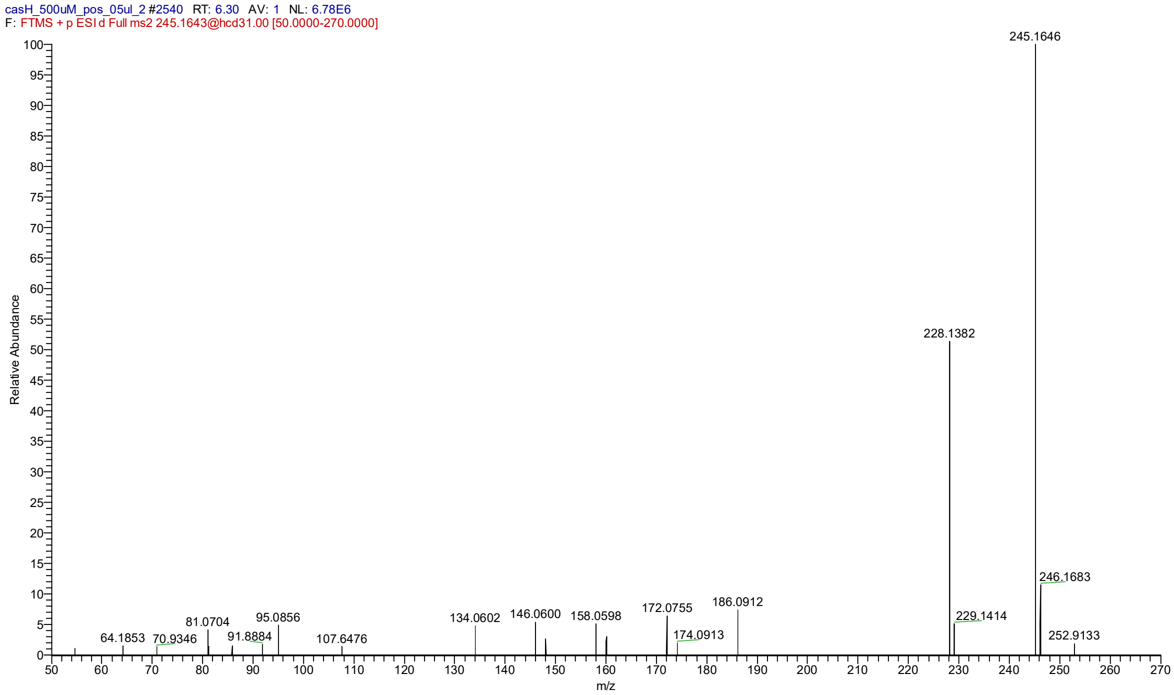

Figure S36. MS/MS spectrum (stepped NCE 18, 30, 45) of **14** (*m/z* 245.1642).

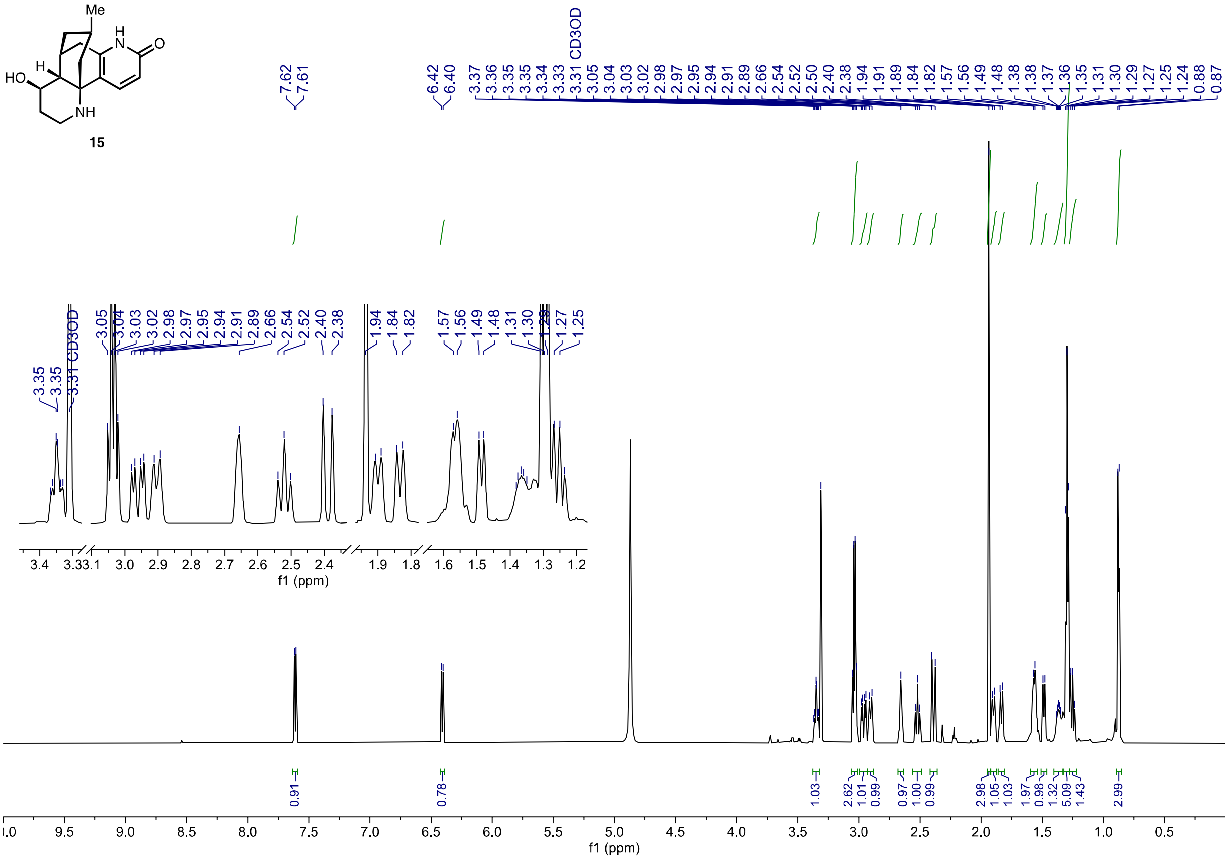

Figure S37. ^{1}H NMR spectrum of lycosquarrine M (**15**) (MeOH-d4, 700 MHz). *N*,*N*-diethylamine acetate as impurity [3.04 (q, *J* = 7.3 Hz), 1.94 (s), 1.30 (t, *J* = 7.3 Hz)].

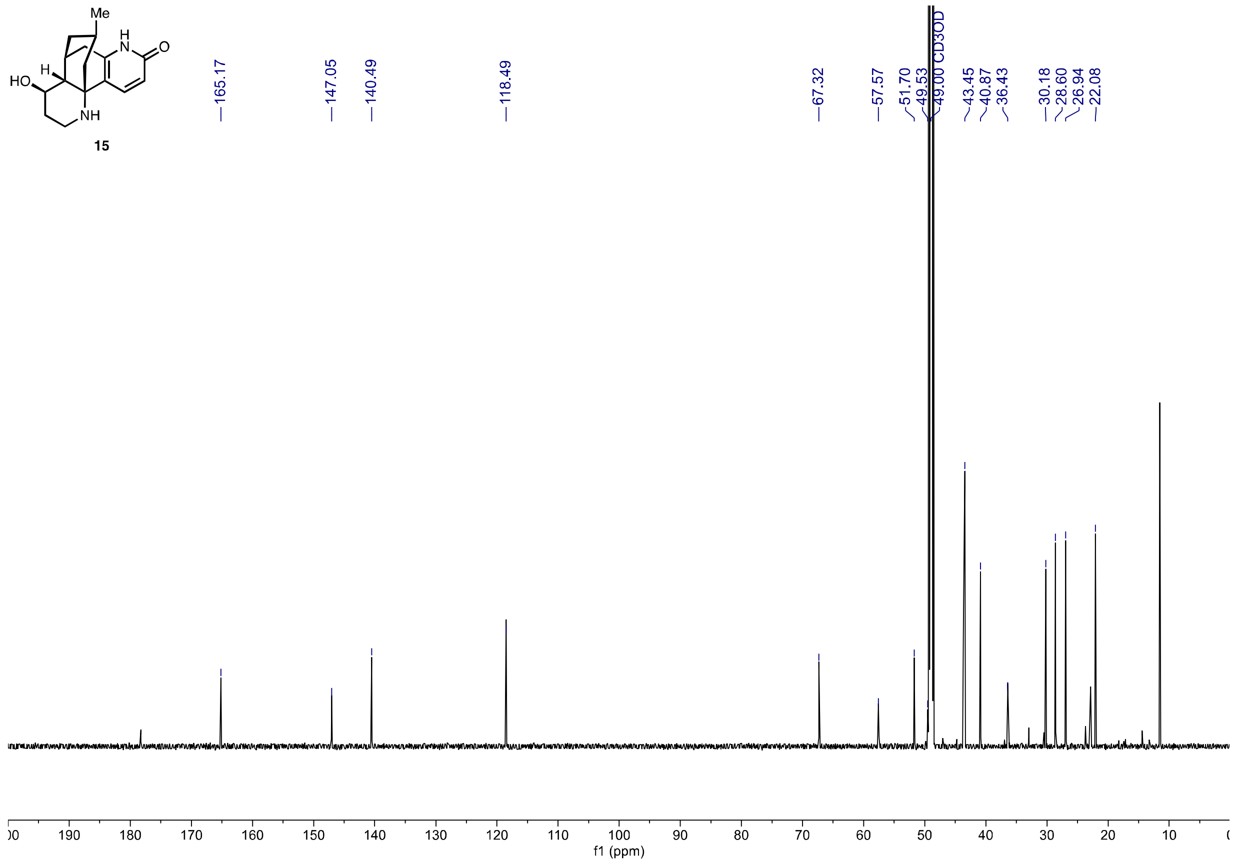

Figure S38. ^{13}C-NMR of 11-epi-lycosquarrine M (**15**) (MeOH-d4, 175 MHz). N,N-diethylamine acetate as impurity (178.3, 43.7, 11.55 ppm).

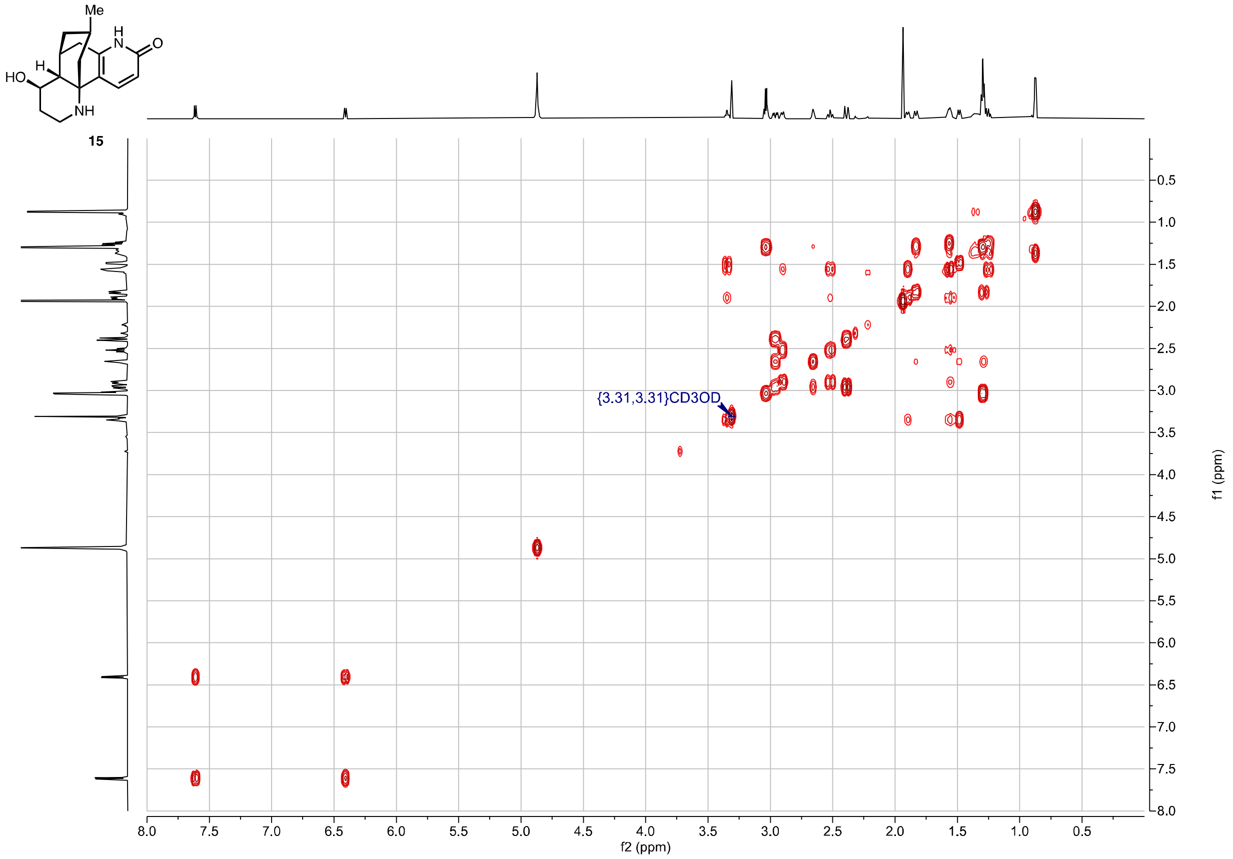

Figure S39. ^{1}H-^{1}H COSY of lycosquarrine M (**15**) (MeOH-d4, 700 MHz).

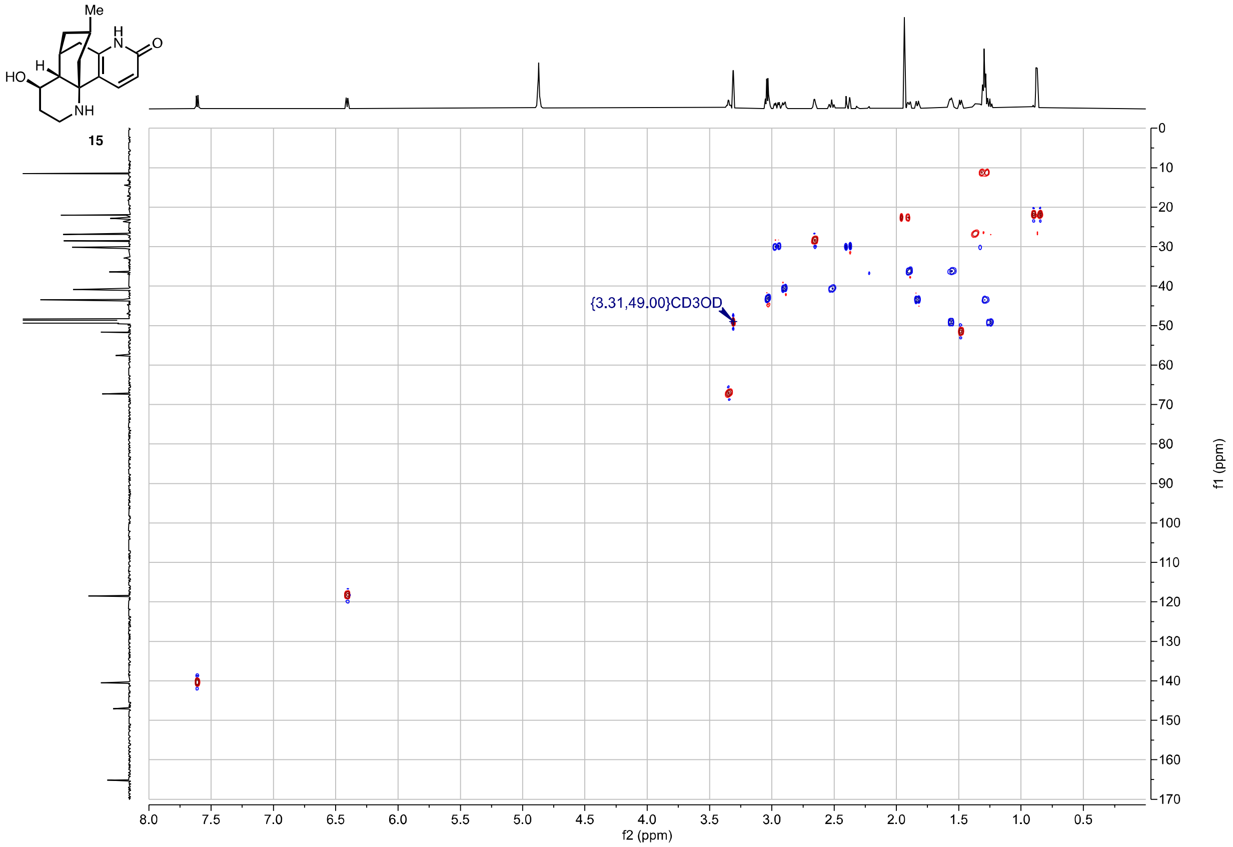

Figure S40. ^{1}H-^{13}C HSQC of lycosquarrine M (**15**) (MeOH-d4).

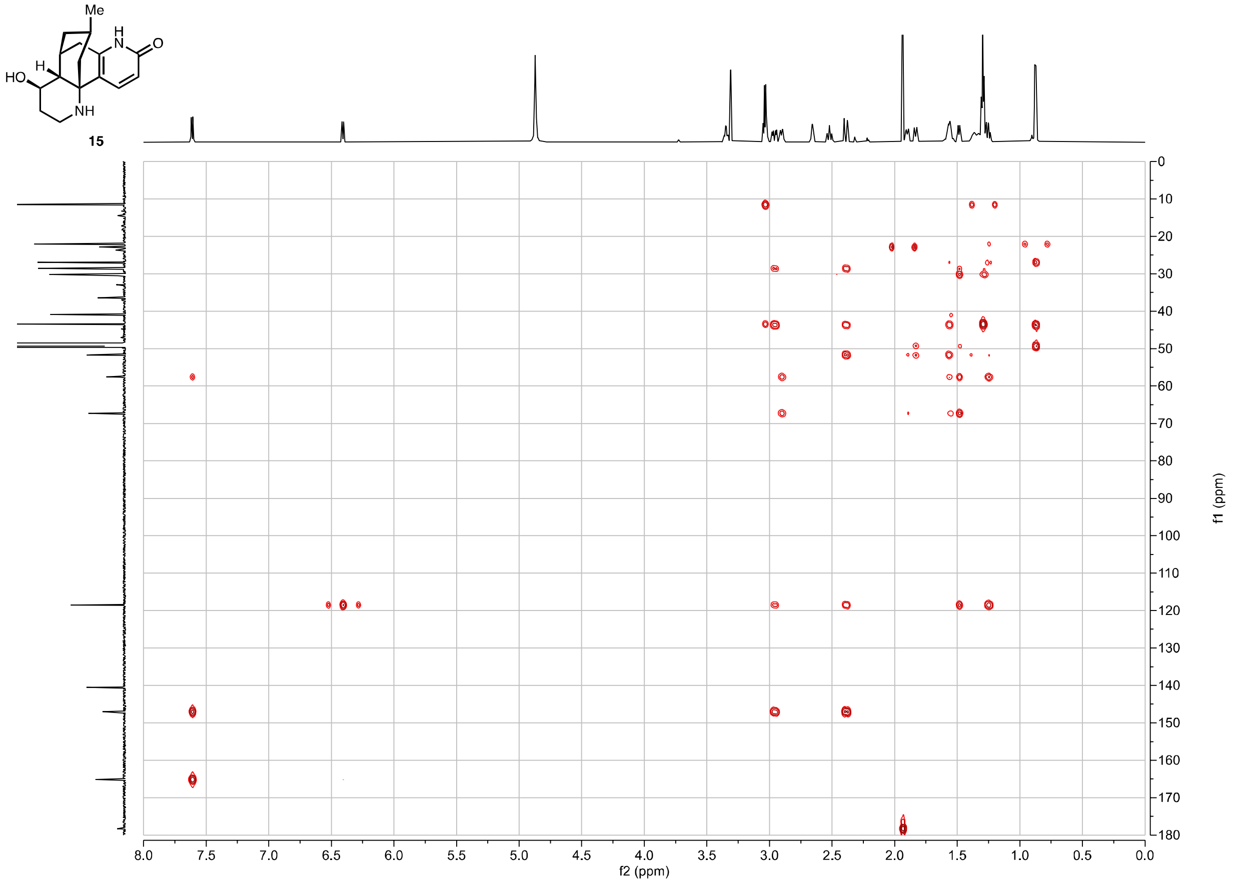

Figure S41. ^{1}H-^{13}C HMBC of lycosquarrine M (**15**) (MeOH-d4).

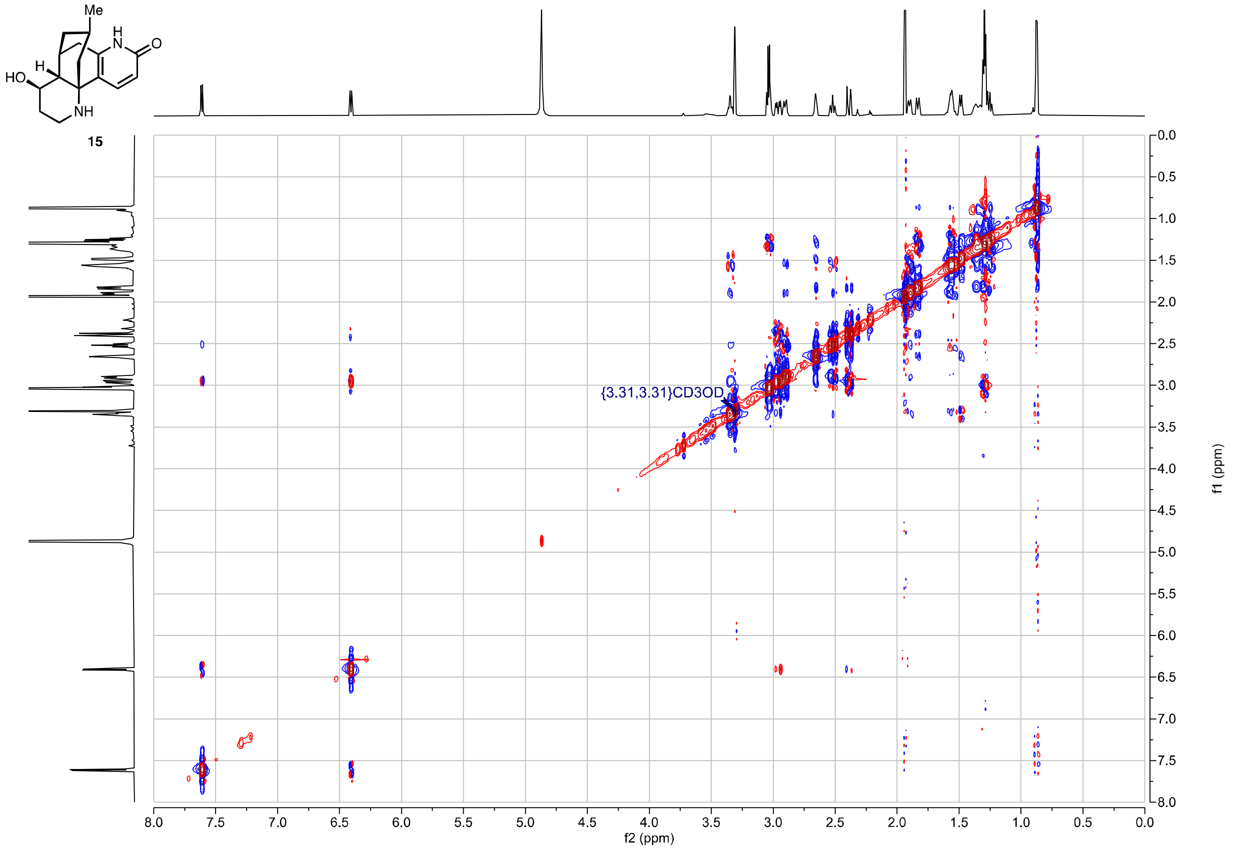

Figure S42. ROESY of lycosquarrine M (**15**) (MeOH-d4).

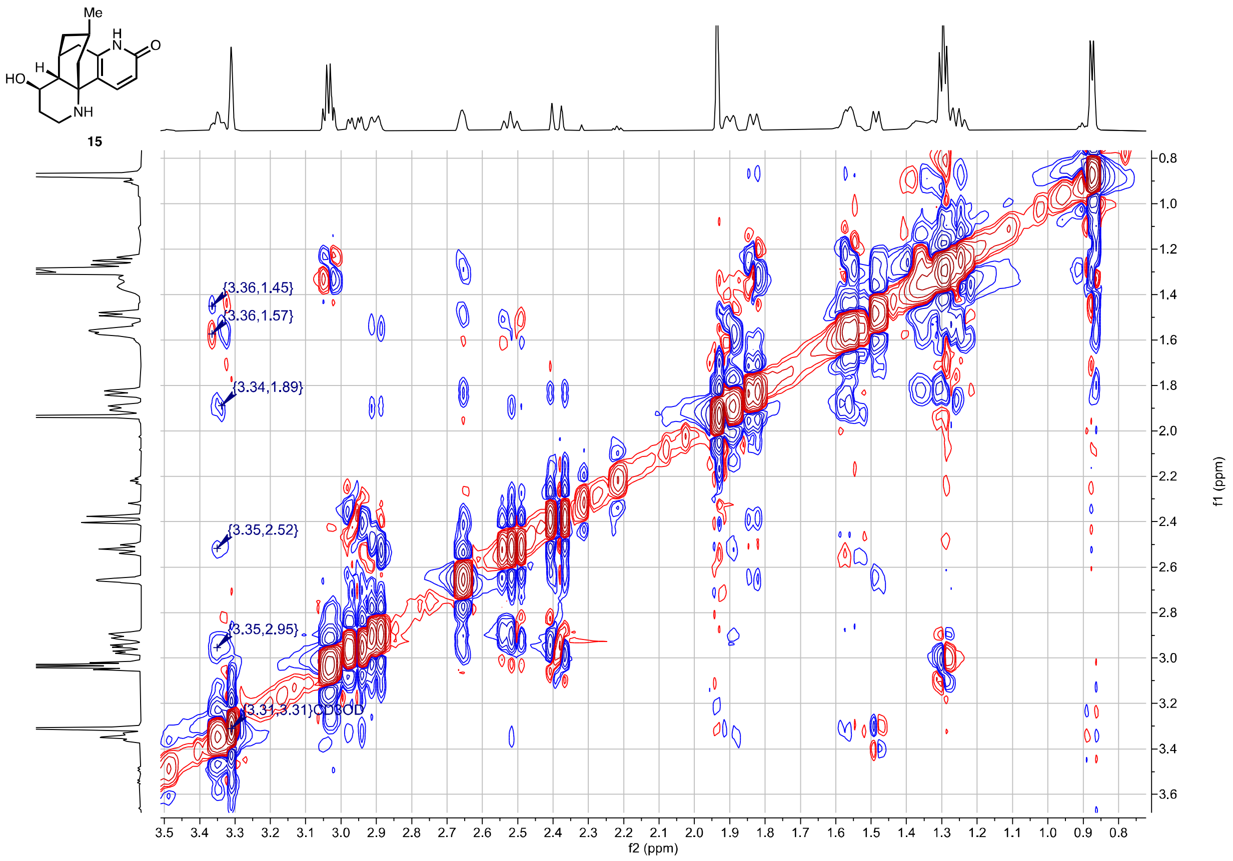

Figure S43. Zoom into the ROESY spectrum of lycosquarrine M (**15**) (MeOH-d4). Correlations to the proton on C11 are annotated.

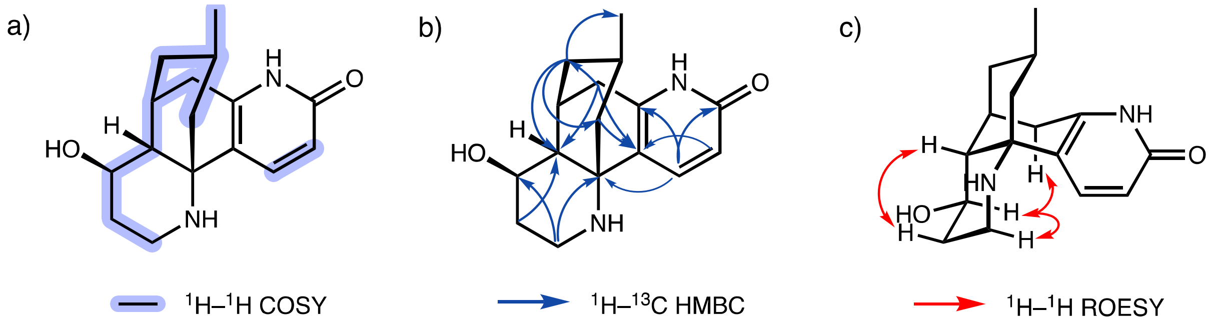

Figure S44. Observed NMR correlations in lycosquarrine M (**15**).

Table S11. Comparison of ^{1}H- and ^{13}C NMR spectroscopic data for lycosquarrine M (**15**) with literature data.

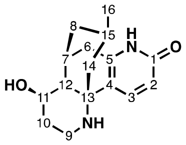

15

	δ_H (ppm)		δ_C (ppm)	
Position	Lit.[a]	Found[b]	Lit.[a]	Found[b]
1	–	–	165.2	165.2
2	6.39, d (9.4)	6.41, d (9.4)	118.3	118.5[c]
3	7.62, d (9.4)	7.61, d (9.3)	140.9	140.5
4	–	–	119.6	118.5[c]
5	–	–	146.8	147.0
6	α 2.95, dd (18.9, 7.1)	α 2.96, dd (18.8, 7.0)	30.3	30.2
	β 2.37, d (18.9)	β 2.39, d (19.0)		
7	2.64, m	2.66, m	28.7	28.6
8	ax 1.28, ddd (12.7, 12.4, 4.0)	ax 1.25, m	43.8	43.5
	eq 1.82, br d (12.7)	eq 1.83, br d (13.8)		
9	α 2.45, td (13.0, 2.7)	α 2.52, t (13.2)	40.9	40.9
	β 2.82, ddd (13.0, 4.6, 2.3)	β 2.90, d (13.2)		
10	a 1.85, m	a 1.90, br d (11.4)	37.0	36.4
	b 1.51, m[c]	b 1.56, m[c]		
11	3.32[c]	3.35, m	67.7	67.3
12	1.43, dd (10.5, 2.9)	1.56, m[c]	52.0	51.7
13	–	–	56.9	57.6
14	ax 1.20, t (11.8)	ax 1.37, m[c]	49.8	49.5
	eq 1.51[c]	eq 1.49, d (10.5)		
15	1.34, m	1.37, m[c]	27.0	26.9
16	0.86, 3H, d (6.2)	0.88, 3H, d (6.4)	22.1	22.1

Coupling constants in parentheses (*J*) are given in Hz. [a]Isolated natural product measured in MeOH-d4 at 500 MHz (^{1}H) and 125 MHz (^{13}C).[22] [b]Sample isolated from Pt2OGD-1 biotransformation measured in MeOH-d4 at 700 MHz (^{1}H) and 175 MHz (^{13}C). [c]Overlapping signals.

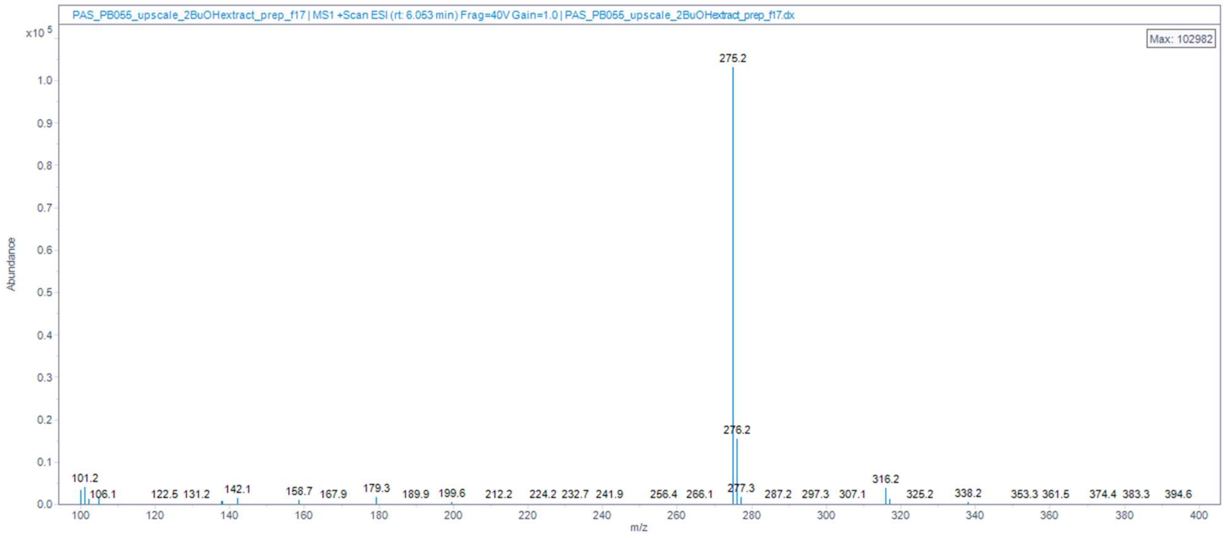

Figure S45. Mass spectrum [LC/API-ES(+)] of lycosquarrine M (**15**).

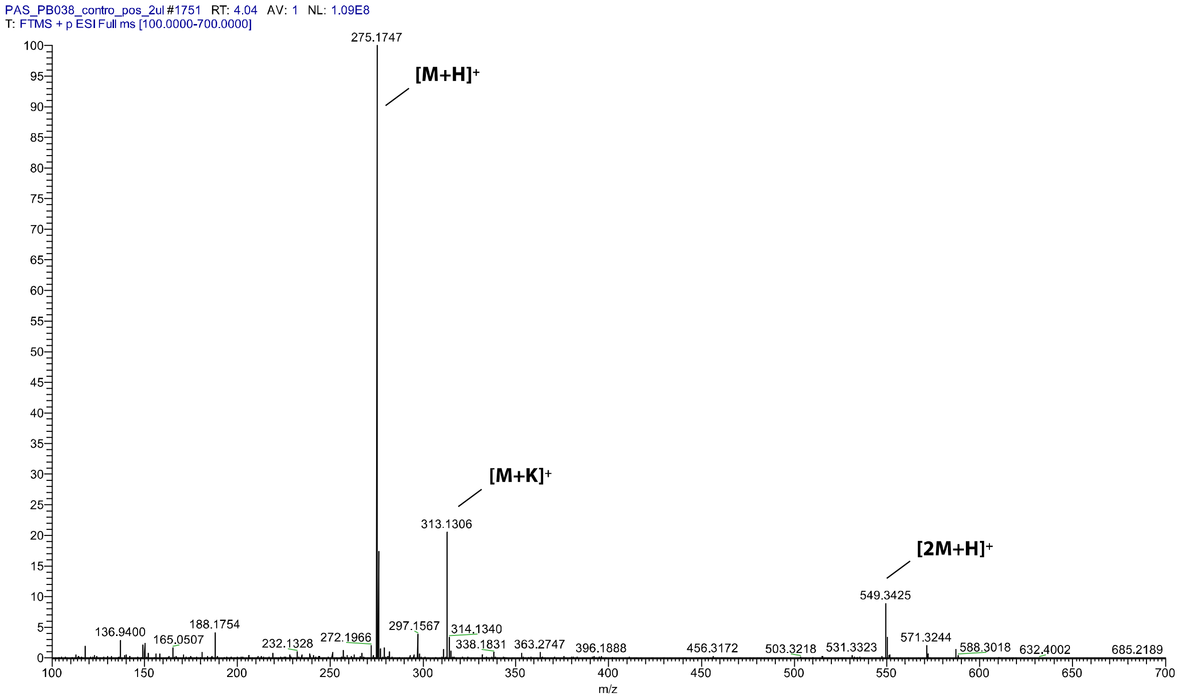

Figure S46. MS spectrum (*m/z* 100-700) of lycosquarrine M (**15**). t_R = 4.04 min, *m/z* 275.1747.: $C_{16}H_{23}N_2O_2$ $[M+H]^+$, Δ – 2.51 ppm.

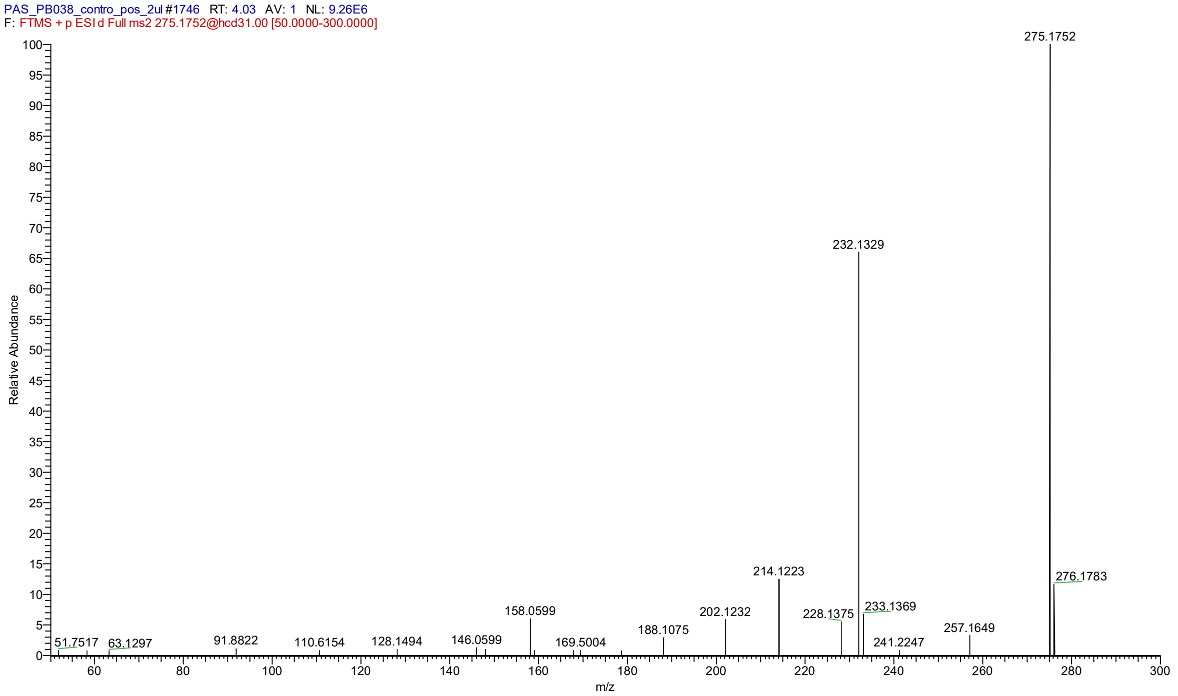

Figure S47. MS/MS spectrum (stepped NCE 18, 30, 45) of **15** (*m/z* 275.1747).

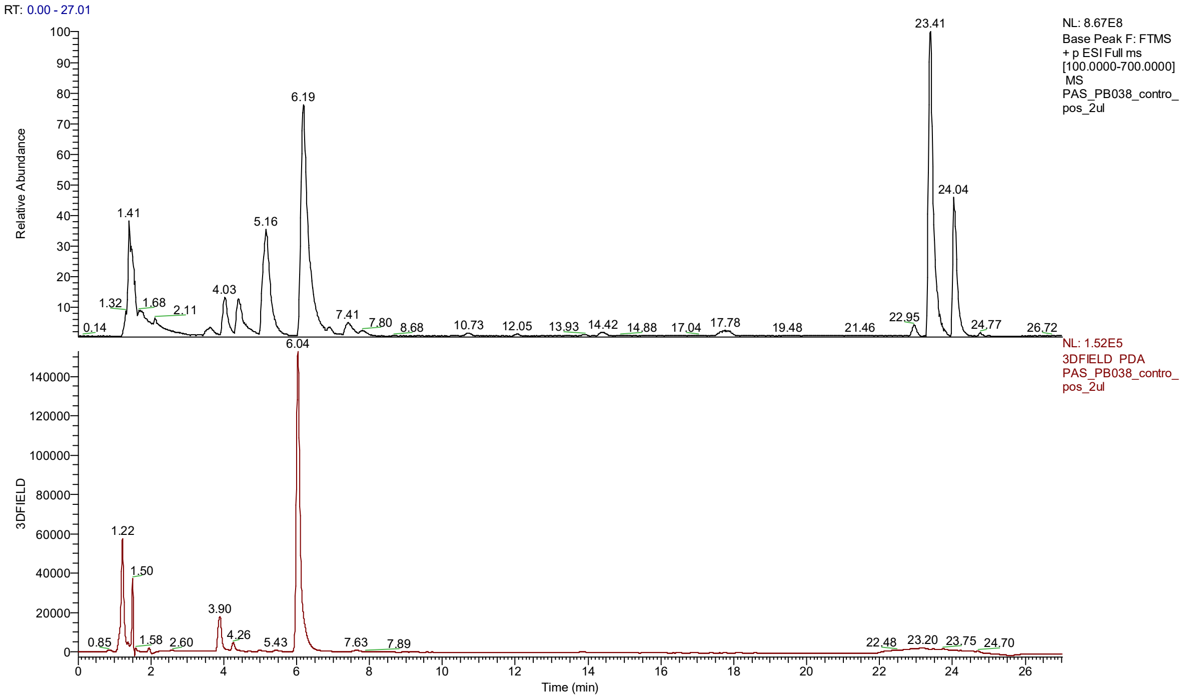

Figure S48. Chromatogram of Pt2OGD-1 biotransformation sample containing lycosquarrine M (**15**) and casuarinine H (**14**) (500 µM total conc., 2 µL); upper trace: HESI full MS, *m/z* 100-700, lower trace: DAD total scan, 210-450 nm).

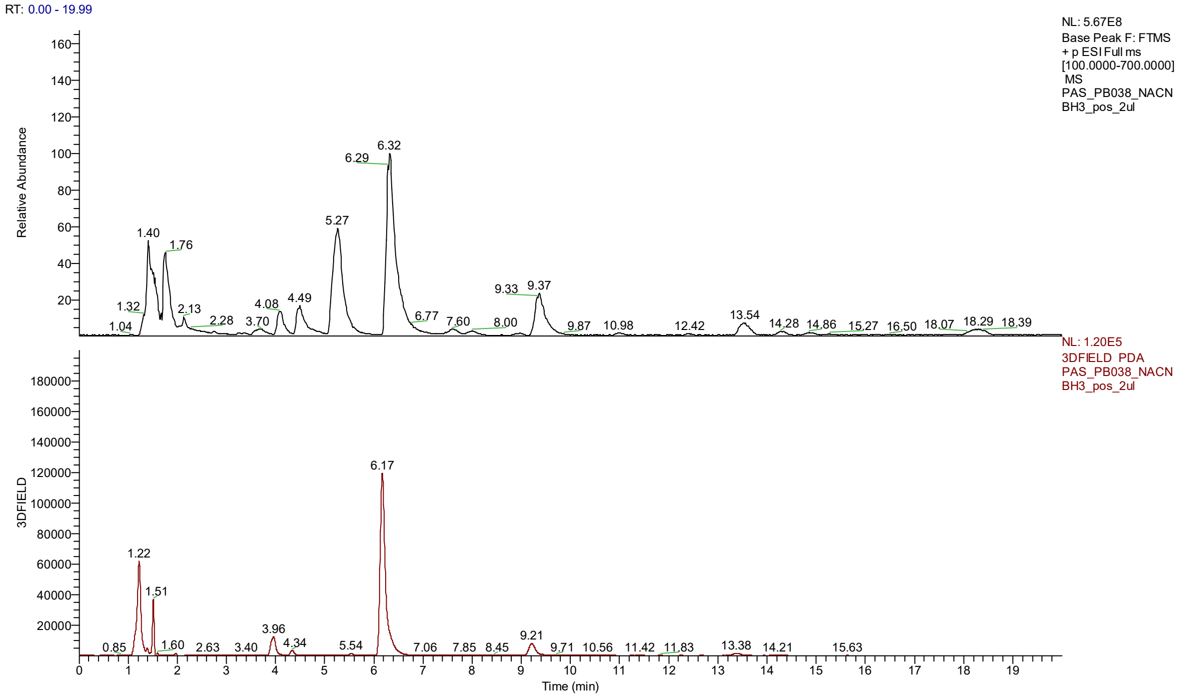

Figure S49. Chromatogram of Pt2OGD-1 biotransformation sample in the presence of $NaCNBH_3$ containing *N*-methyl-casuarinine H (**16**) and casuarinine H (**14**), (500 µM total conc., 2 µL injection); upper trace, HESI full MS, *m/z* 100-700; lower trace, DAD total scan, 210-450 nm).

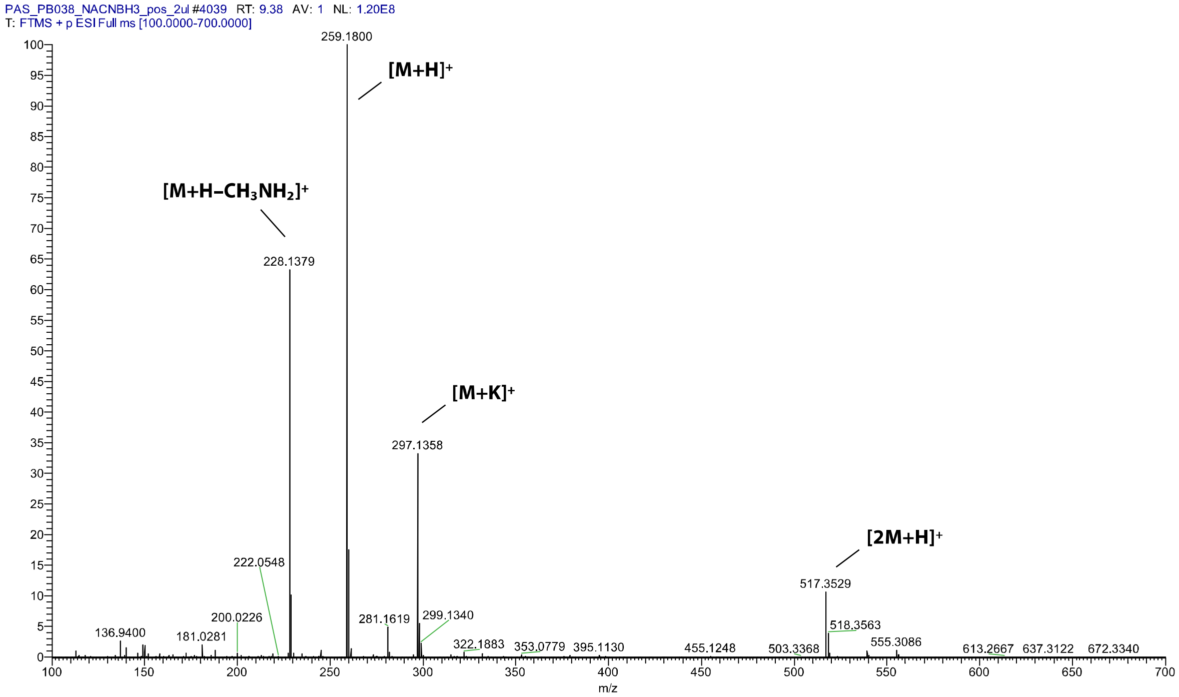

Figure S50. MS spectrum (*m/z* 100-700) of *N*-methyl-casuarinine H (**16**). t_R = 8.38 min, *m/z* 259.1800: $C_{16}H_{23}N_2O$ $[M+H]^+$, Δ −2.04 ppm.

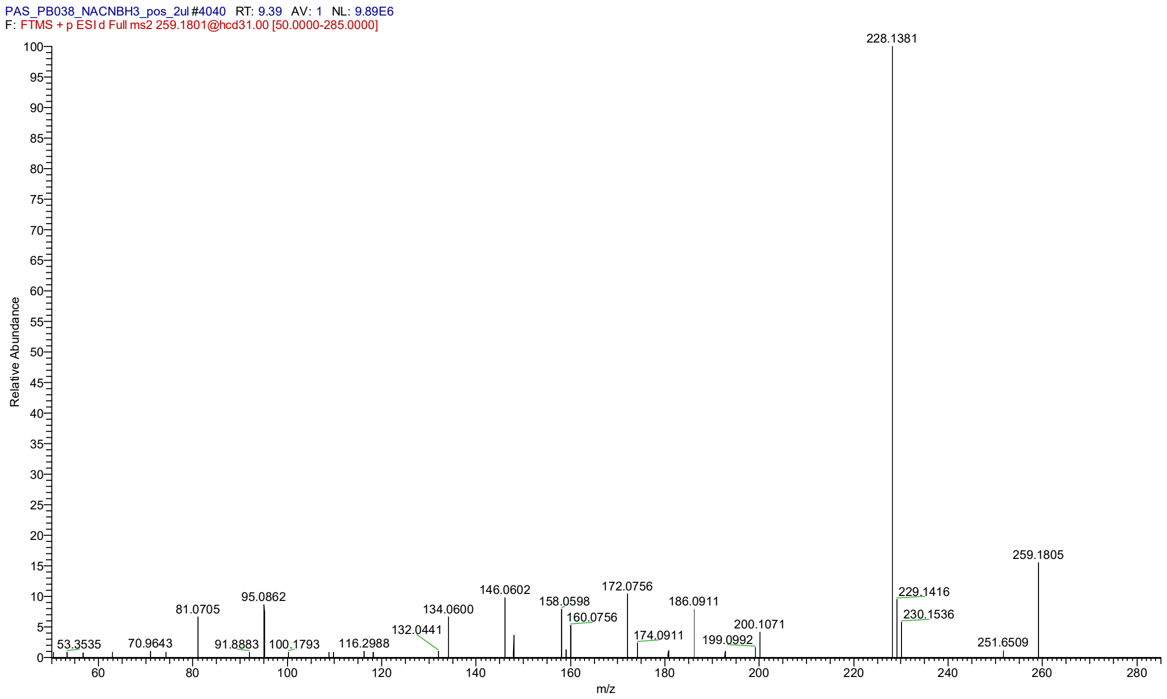

Figure S51. MS/MS spectrum (stepped NCE 18, 30, 45) of **16** *m/z* 259.1800.

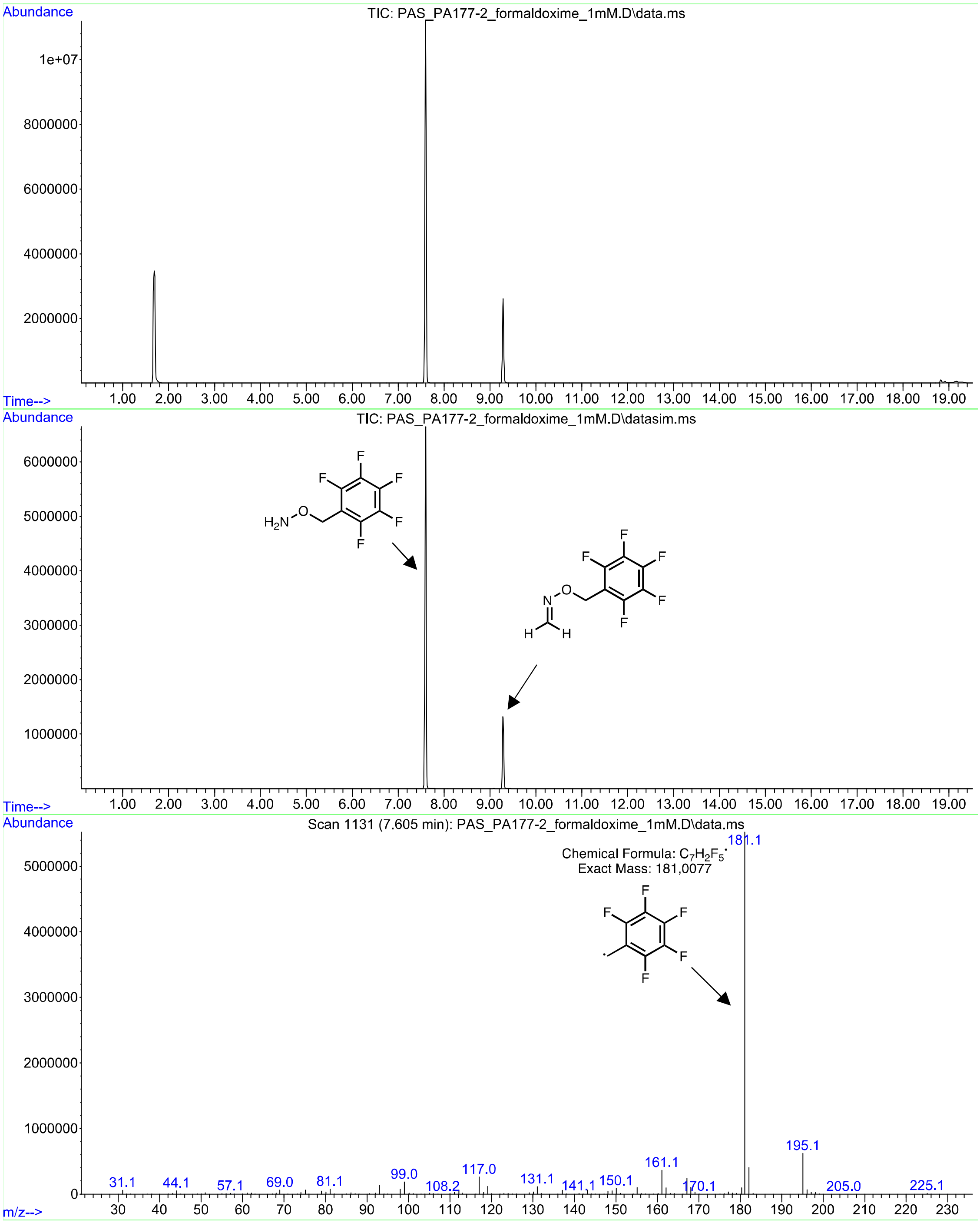

Figure S52.Headspace chromatograms and extracted mass spectrum of a derivatized formaldehyde standard. Upper panel: Chromatogram in full-scan mode (mass range 30–230). Middle panel: Chromatogram in SIM mode (*m/z* 181, 195). Lower panel: Full spectrum of the derivatized oxime analyte (t_R 7.60 min).

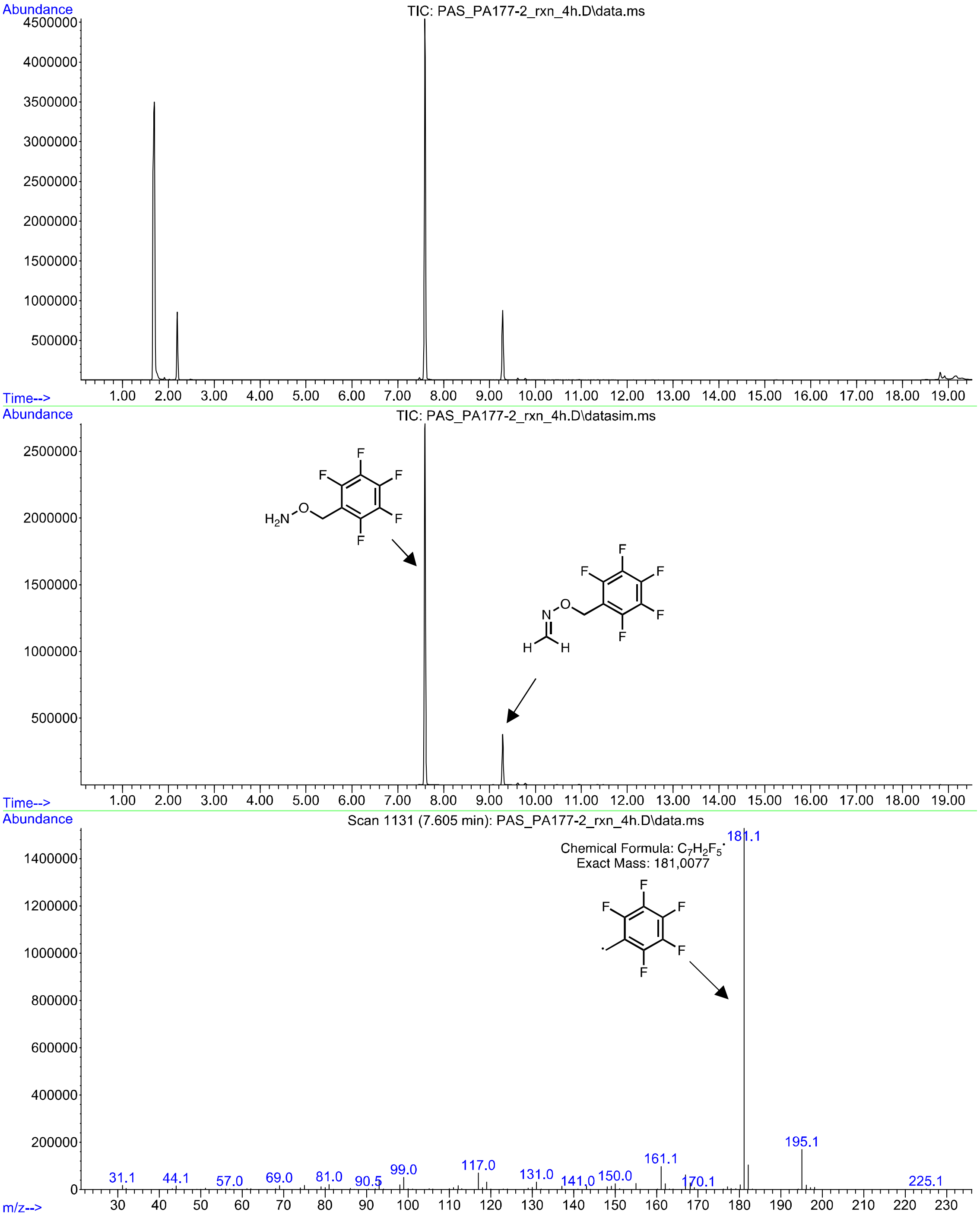

Figure S53 Headspace chromatograms and extracted mass spectrum of a derivatized biotransformation sample. Upper panel: Chromatogram in full-scan mode (mass range 30–230). Middle panel: Chromatogram in SIM mode (*m/z* 181, 195). Lower panel: Full spectrum of the derivatized oxime analyte (t_R 7.60 min). The found analyte peak matches the formaldehyde standard.

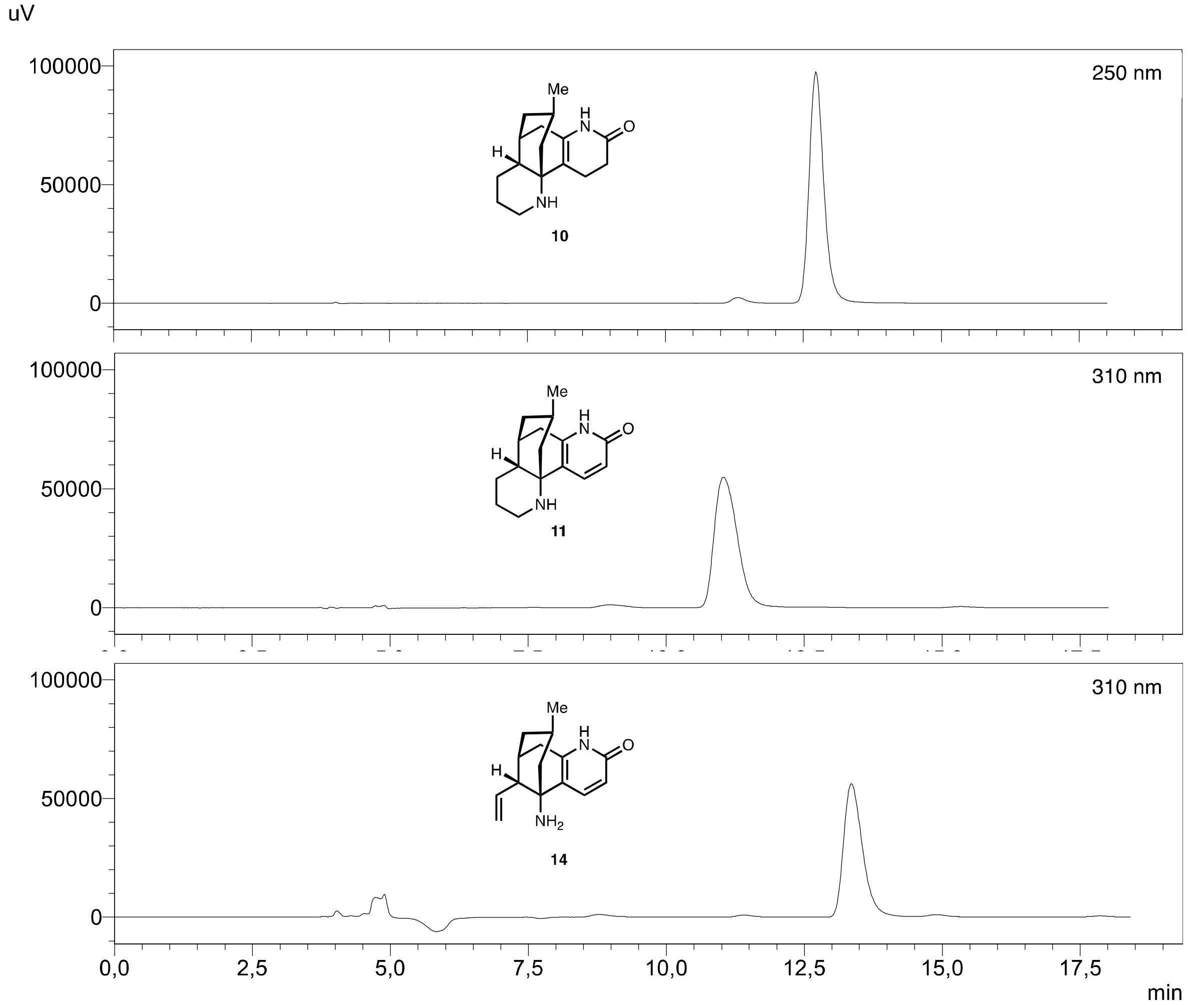

Figure S54. Example chromatograms for the chromatographic separation and detection of **10**, **11** and **14** (standard samples) using *Method B*.

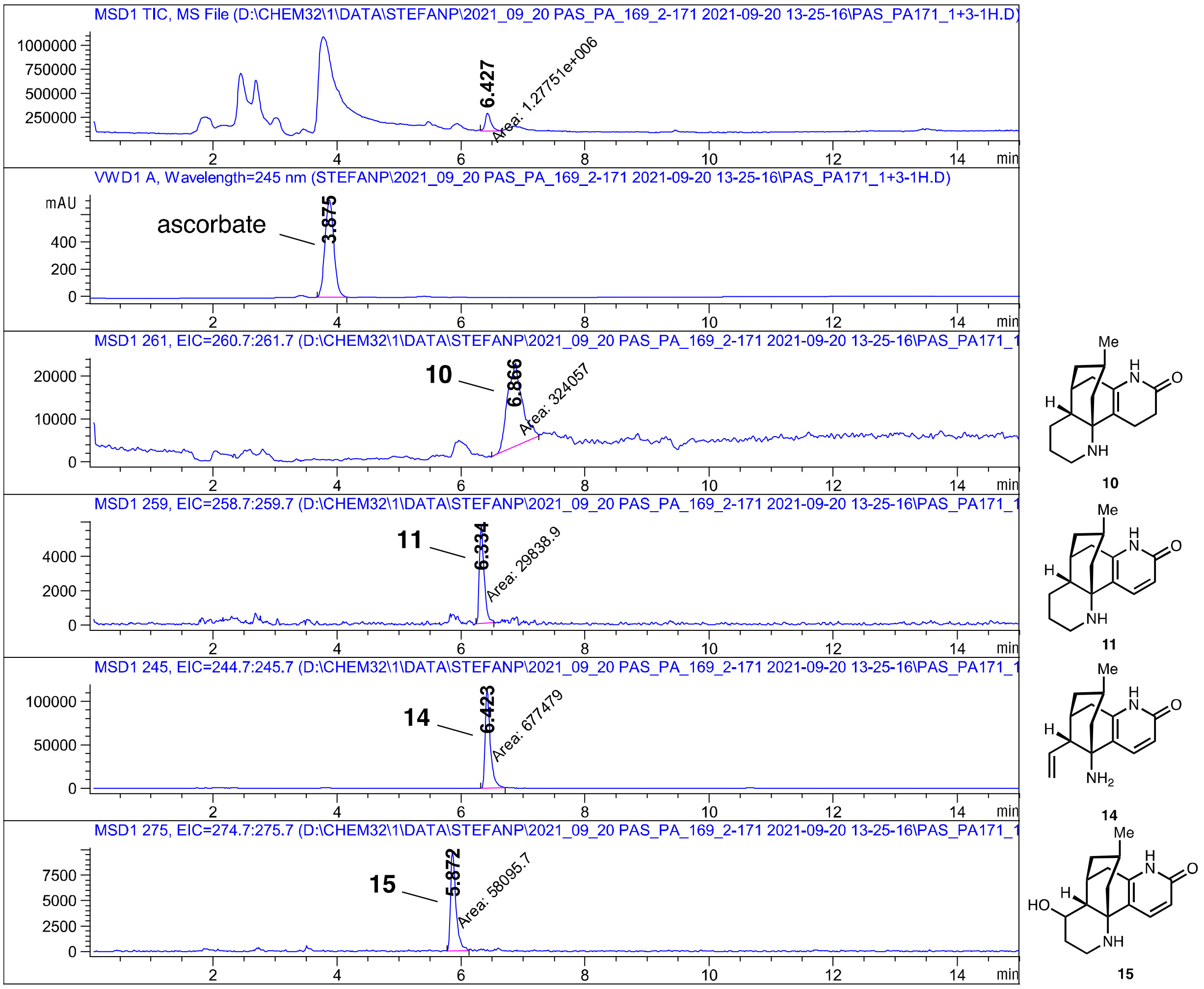

Figure S55. Example chromatograms for the chromatographic separation and mass-selective detection of **10**, **11**, **14** and **15** in a typical biotransformation sample using *Method A.*

10. References

(1) https://www.jameslingford.com/blog/foldseek-cofactors/, accessed on Nov. 11, 2023.

(2) van Kempen, M.; Kim, S.; Tumescheit, C.; Mirdita, M.; Lee, J.; Gilchrist, C.L.M; Söding, J.; and Steinegger, M. Fast and accurate protein structure search with Foldseek. *Nature Biotechnol.*, **2024**, 42, 243–246. DOI: 10.1038/s41587-023-01773-0.

(3) https://search.foldseek.com/search, accessed on Nov. 11, 2023

(4) Mirdita, M.; Schütze, K.; Morikawi, Y.; Heo, L.; Ovchinnikov, S.; Steinegger, M. ColabFold: Making protein folding accessible to all. *Nat. Methods* **2022**, *19*, 679–682. DOI: 10.1038/s41592-022-01488-1.

(5) Aik, WS.; McDonough, M.A.; Thalhammer, A.; Chowdhury, R.; Schofield, C.J. Role of the jelly-roll fold in substrate binding by 2-oxoglutarate oxygenases. *Curr. Opin. Struct. Biol.* **2012**, *22*, 691–700. DOI: 10.1016/j.sbi.2012.10.001.

(6) Pettersen, E.F.; Goddard, T.D.; Huang, C.C.; Couch, G.S.; Greenblatt, D.M.; Meng, E.C.; Ferrin, T.E. UCSF Chimera – a visualization system for exploratory research and analysis. *J. Comput. Chem*. **2004**, *25*, 1605-1612. DOI: 10.1002/jcc.20084.

(7) https://www.rbvi.ucsf.edu/chimera. Accessed on Nov. 3, 2023.

(8) Krieger, E.; Joo, K.; Lee, J.; Lee, J.; Raman, S.; Thompson, J.; Tyka, M.; Baker, D.; Karplus, K. Improving physical realism, stereochemistry, and side-chain accuracy in homology modeling: Four approaches that performed well in CASP8. *Proteins* **2009,** *77*, 114-122. DOI: 10.1002/prot.22570.

(9) http://www.yasara.org/minimizationserver.php. Accessed on Nov. 11, 2023.

(10) Shapalov, M.S., Dunbrack, R.L. Jr., A Smoothed Backbone-Dependent Rotamer Library for Proteins Derived from Adaptive Kernel Destiny Estimates and Regressions. *Structure* **2011**, 19, 844-858. DOI: 10.1016/j.str.2011.03.019.

(11) Hanwell, M.D.; Curtis, D.E.; Lonie, D.C.; Vandermeersch, T.; Zurek, E.; Hutchison, G.R. Avogadro: An advanced semantic chemical editor, visualization, and analysis platform. *J. Cheminf.* **2012**, *4*, 17. DOI: 10.1186/1758-2946-4-17.

(12) http://avogadro.cc/. Accessed on June 5, 2023.

(13) Wilmouth, R.C.; Turnbull, J.J.; Welford, R.W.D.; Clifton, I.J.; Prescott, A.G.; Schofield, C.J. Structure and Mechanism of Anthocyanidin Synthase from Arabidopsis thaliana. *Structure* **2002**, *10*, 93–103. DOI: 10.1016/S0969-2126(01)00695-5.

(14) Butt, S.S.; Badshah, Y.; Shabbir, M.; Rafiq, M. Molecular Docking Using Chimera and Autodock Vina Software for Nonbioinformaticians. *JMIR Bioinform. Biotech*. **2020**, *1*, e14232. DOI: 10.2196/14232.

(15) Takeuchi, A.; Takigawa, T.; Abe, M.; Kawai, T.; Endo, Y.; Yasugi, T.; Endo, G.; Ogino, K. Determination of Formaldehyde in Urine by Headspace Gas Chromatography. *Bull. Environ. Contam. Toxicol.* **2007**, *79*, 1-4. DOI: 10.1007/s00128-007-9172-0.

(16) a) Lee, C.; Yang, W.; Parr, R. G. Development of the Colle-Salvetti correlation-energy formula into a functional of the electron density. *Phys. Rev. B: Condens. Matter Mater. Phys.* **1988**, *37*, 785–789. DOI:

10.1103/PhysRevB.37.785; (b) Becke, A. D. Density-functional thermochemistry. III. The role of exact exchange. *J. Chem. Phys.* **1993**, *98*, 5648–5652, DOI: 10.1063/1.464913; (c) Grimme, S.; Antony, J.; Ehrlich, S.; Krieg, H. A consistent and accurate ab initio parametrization of density functional dispersion correction (DFT-D) for the 94 elements H-Pu. *J. Chem. Phys.* **2010**, *132*, 154104. DOI: 10.1063/1.3382344; (d) Grimme, S.; Ehrlich, S.; Goerigk, L. Effect of the damping function in dispersion corrected density functional theory. *J. Comput. Chem.* **2011**, *32*, 1456–1465. DOI: 10.1002/jcc.21759.

(17) Frisch, M. J.; Trucks, G. W.; Schlegel, H. B.; Scuseria, G. E.; Robb, M. A.; Cheeseman, J. R.; Scalmani, G.; Barone, V.; Petersson, G. A.; Nakatsuji, H.; Li, X.; Caricato, M.; Marenich, A. V.; Bloino, J.; Janesko, B. G.; Gomperts, R.; Mennucci, B.; Hratchian, H. P.; Ortiz, J. V.; Izmaylov, A. F.; Sonnenberg, J. L.; Williams-Young, D.; Ding, F.; Lipparini, F.; Egidi, F.; Goings, J.; Peng, B.; Petrone, A.; Henderson, T.; Ranasinghe, D.; Zakrzewski, V. G.; Gao, J.; Rega, N.; Zheng, G.; Liang, W.; Hada, M.; Ehara, M.; Toyota, K.; Fukuda, R.; Hasegawa, J.; Ishida, M.; Nakajima, T.; Honda, Y.; Kitao, O.; Nakai, H.; Vreven, T.; Throssell, K.; Montgomery, J. A., Jr.; Peralta, J. E.; Ogliaro, F.; Bearpark, M. J.; Heyd, J. J.; Brothers, E. N.; Kudin, K. N.; Staroverov, V. N.; Keith, T. A.; Kobayashi, R.; Normand, J.; Raghavachari, K.; Rendell, A. P.; Burant, J. C.; Iyengar, S. S.; Tomasi, J.; Cossi, M.; Millam, J. M.; Klene, M.; Adamo, C.; Cammi, R.; Ochterski, J. W.; Martin, R. L.; Morokuma, K.; Farkas, O.; Foresman, J. B.; Fox, D. J. Gaussian 16, revision 01,C; Gaussian, Inc.: Wallingford CT, 2016.

(18) Marenich, A. V.; Cramer, C. J.; Truhlar, D. G. Universal solvation model based on solute electron density and on a continuum model of the solvent defined by the bulk dielectric constant and atomic surface tensions. *J. Phys. Chem. B* **2009**, *113*, 6378–6396. DOI: 10.1021/jp810292n.

(19) Haley, H. M. S.; Payer, S. E.; Papidocha, S. M.; Clemens, S.; Nyenhuis, J.; Sarpong, R., Bioinspired Diversification Approach Toward the Total Synthesis of Lycodine-Type Alkaloids. *J. Am. Chem. Soc.* **2021**, *143*, 4732–4740. DOI: 10.1021/jacs.1c00457.

(20) Fischer, D. F.; Sarpong, R., Total Synthesis of (+)-Complanadine A Using an Iridium-Catalyzed Pyridine C–H Functionalization. *J. Am. Chem. Soc.* **2010,** *132*, 5926–5927. DOI: 10.1021/ja101893b.

(21) Fu, J.-G.; Xu, G.-Q.; Ding, R.; Lin, G.-Q.; Sun, B.-F. Asymmetric total synthesis of Lycopodium alkaloids α-obscurine, N-desmethyl-α-obscurine, β-obscurine and N-desmethyl-β-obscurine. *Org. Chem. Front.* **2016**, *3*, 62–65. DOI: 10.1039/C5QO00355E.

(22) Zhu, X.; Xia, D.; Zhou, Z.; Xie, S.; Shi, Z.; Chen, G.; Wang, L.; Pan, K., Lycosquarrines A–R, Lycopodium Alkaloids from *Phlegmariurus squarrosus*. *J. Nat. Prod.* **2020**, *83*, 2831–2843. DOI: 10.1021/acs.jnatprod.9b00815.
