# Supplementary material for: C–C Bond Cleavage in the Late-Stage Biosynthesis of Huperzine Alkaloids Occurs via Enzymatic Retro-Aza-Prins Reaction
Source: J Am Chem Soc. 2025 May 9;147(24):20265–72. doi: 10.1021/jacs.4c10410 (PMC12186474; doi:10.1021/jacs.4c10410)
Supplement: Supplementary file 6 [file ja4c10410_si_006.pdf]

Cartesian coordinates for

C-C Bond Cleavage in the Late-Stage Biosynthesis of Huperzine Alkaloids Occurs via Enzymatic Retro-Aza-Prins Reaction

Stefan E. Payer,[a,b] Mario Prejanò,[c,d] Philipp Kögl,[a] Tamara Reiter,[a] Eva-Maria Pferschy-Wenzig,[e] Fahmi Himo[c]* and Wolfgang Kroutil[a,f]*

[a]Institute of Chemistry, University of Graz, BioTechMed Graz, Heinrichstrasse 28, A-8010 Graz, Austria.

[b]Enzyan Biocatalysis GmbH, Stiftingtalstraße 14, A-8010 Graz, Austria.

[c]Department of Organic Chemistry, Arrhenius Laboratory, Stockholm University, SE-106 91 Stockholm, Sweden.

[d]Dipartimento di Chimica e Tecnologie Chimiche, Università della Calabria, Via P. Bucci, 87036 Rende, Italy.

[e]Institute of Pharmaceutical Sciences, Pharmacognosy, University of Graz, Beethovenstrasse 8, A-8010 Graz, Austria.

[f]Field of Excellence BioHealth – University of Graz, 8010 Graz, Austria.

Corresponding Authors

Fahmi Himo fahmi.himo@su.se

Wolfgang Kroutil wolfgang.kroutil@uni-graz.at

11

C	0.893500	-0.413110	-0.614050
C	1.314700	-1.873960	1.440550
C	1.611430	-3.048710	0.502410
C	0.853900	-2.870040	-0.819760
H	1.899830	-1.953090	2.364930
H	2.690560	-3.094610	0.298760
H	1.335000	-4.000260	0.971810
H	1.106720	-3.667000	-1.526680
H	-0.224150	-2.943700	-0.631740
H	0.257840	-1.912470	1.730030
N	1.106930	-1.584920	-1.480410
H	2.069650	-1.563030	-1.809840
C	1.624260	-0.540240	0.748680
H	2.701910	-0.528820	0.527170
C	1.310480	0.701990	1.598510
H	1.844230	0.622170	2.553950
C	1.449110	0.824480	-1.353320
H	0.956210	0.906980	-2.328190
H	2.519880	0.658850	-1.547710
C	1.826180	1.959130	0.873730
H	2.922370	1.896820	0.824120
H	1.593700	2.854840	1.463400
C	1.281020	2.124800	-0.555400
H	0.206560	2.337080	-0.489810
C	1.946390	3.304610	-1.268000
H	1.538310	3.440060	-2.274810
H	1.794460	4.237570	-0.715100
H	3.027060	3.145910	-1.363450
C	-0.196110	0.768520	1.918930
H	-0.423220	0.123480	2.778540
H	-0.467080	1.787210	2.226610
C	-0.602120	-0.230330	-0.381800
C	-1.071580	0.345530	0.769620
C	-1.555430	-0.572900	-1.389570
H	-1.170610	-1.020910	-2.300210
C	-3.424860	0.221830	-0.013330
C	-2.893820	-0.367660	-1.226410
H	-3.614850	-0.633400	-1.990130
N	-2.418390	0.543210	0.919750
H	-2.770690	0.967890	1.769060
O	-4.601970	0.453340	0.251140

Fe^{IV}=O

O	-2.100495	-0.962290	2.217737
Fe	-3.189673	-0.595130	1.077964
N	-4.552340	-0.076726	-0.451139
C	-5.784795	0.334927	-0.210892
C	-4.362133	-0.028348	-1.817770
H	-6.215664	0.419901	0.773700
C	-5.510318	0.417922	-2.413144
H	-3.417925	-0.316458	-2.249143
H	-5.770292	0.591150	-3.444800
N	-2.429233	1.344084	0.823832
C	-2.792706	2.468566	1.537577
C	-1.481605	1.686453	-0.027998
C	-2.038560	3.521684	1.093920
H	-3.539517	2.407220	2.319268
H	-0.995925	1.010795	-0.714303
H	-2.005272	4.558379	1.387280
C	-2.355949	-2.338044	-0.542514
O	-3.254537	-2.521566	0.342490
O	-1.849291	-1.190531	-0.707181
C	-1.895789	-3.515651	-1.367638
H	-2.733191	-4.184291	-1.576366
H	-1.431030	-3.176582	-2.294718
H	-1.154035	-4.076724	-0.789500
O	-4.500152	1.250697	3.959056
N	-1.216415	3.010186	0.102227
H	-0.524606	3.523834	-0.420940
N	-6.401545	0.642847	-1.377708
H	-7.348969	0.975659	-1.467520
C	-4.510340	0.102520	3.536107
O	-4.632568	-0.174535	2.241054
C	-4.407841	-1.110814	4.433928
H	-3.461237	-1.613907	4.213331
H	-4.441161	-0.815895	5.483269
H	-5.212894	-1.815877	4.207509

Fe^{IV}=O-11

C	4.153180	-0.944347	-0.483295
C	2.153225	-2.540356	-0.512910
C	2.763336	-3.222928	-1.741694
C	3.631933	-2.227804	-2.522300
H	1.590646	-3.258894	0.094999
H	3.385304	-4.072820	-1.424943
H	1.977017	-3.626915	-2.390532
H	4.126848	-2.716647	-3.368460
H	2.991398	-1.441972	-2.941041
H	1.427323	-1.788418	-0.840058
N	4.668392	-1.576438	-1.710998
H	5.360368	-2.271268	-1.438391
C	3.251959	-1.897464	0.344368
H	3.906325	-2.706661	0.703353
C	2.725968	-1.136445	1.571031
H	2.105294	-1.817028	2.167405
C	5.365219	-0.538603	0.383949
H	6.022840	0.111669	-0.204172
H	5.940004	-1.447090	0.621033
C	3.912017	-0.689754	2.445656
H	4.406673	-1.588966	2.840275
H	3.546386	-0.129385	3.315688
C	4.957043	0.151689	1.693054
H	4.496786	1.112994	1.431643

C	6.176049	0.441329	2.572468
H	6.906904	1.064187	2.045826
H	5.886384	0.964874	3.489923
H	6.677972	-0.489036	2.864260
C	1.821754	0.028982	1.130996
H	0.814273	-0.340537	0.910061
H	1.708136	0.753661	1.945226
C	3.369895	0.306822	-0.864841
C	2.317032	0.745403	-0.095701
C	3.742461	1.093362	-1.986861
H	4.581522	0.746058	-2.581678
C	1.926361	2.669321	-1.569768
C	3.064019	2.232287	-2.329387
H	3.332876	2.829390	-3.192430
N	1.642568	1.881342	-0.457400
H	0.831027	2.142058	0.113994
O	1.211385	3.657775	-1.854012
O	-0.945333	-0.854482	-0.355816
Fe	-2.472736	-0.515692	0.072234
N	-4.469335	-0.138381	0.529173
C	-4.905262	0.290523	1.700375
C	-5.567822	-0.296104	-0.293003
H	-4.270534	0.503257	2.545126
C	-6.693243	0.051512	0.403259
H	-5.449012	-0.660059	-1.300246
H	-7.735695	0.069342	0.129878
N	-2.527859	1.174011	-1.132960
C	-3.310889	2.306602	-0.982112
C	-1.363338	1.587990	-1.626705
C	-2.590900	3.396298	-1.390947
H	-4.317195	2.251286	-0.601522
H	-0.513824	0.948906	-1.793696
H	-2.839602	4.444503	-1.431821
C	-3.318972	-2.829170	-0.426429
O	-2.869473	-2.480529	0.714169
O	-3.388182	-1.981062	-1.363708
C	-3.739493	-4.261038	-0.653515
H	-2.890966	-4.810438	-1.074828
H	-4.023327	-4.732834	0.288338
H	-4.560472	-4.304047	-1.371690
O	-2.096111	0.337498	1.822491
N	-1.365132	2.916075	-1.809147
H	-0.473169	3.430059	-1.958039
N	-6.253258	0.419742	1.664526
H	-6.833070	0.719812	2.433009
C	-1.217694	1.264128	2.065229
O	-0.647061	1.962460	1.221862
C	-0.901637	1.430817	3.545591
H	-1.811281	1.389779	4.150128
H	-0.266201	0.595329	3.860041
H	-0.371746	2.368386	3.718434

TS_{HT}

C	-3.383388	-1.093882	0.677746
C	-1.419003	-2.278272	-0.484297
C	-1.070060	-2.945141	0.833363
C	-1.570295	-2.086062	2.000267
H	-1.100117	-2.850348	-1.362079
H	-1.550676	-3.936558	0.879202
H	0.010925	-3.089914	0.908790
H	-1.376965	-2.577240	2.959338
H	-1.017628	-1.140813	2.001916
H	-0.671886	-1.318871	-0.542478
N	-3.003497	-1.766320	1.928980
H	-3.542230	-2.626888	2.004474
C	-2.870268	-1.837514	-0.585656
H	-3.458979	-2.769764	-0.640654
C	-3.223225	-1.008085	-1.832362
H	-2.865827	-1.539429	-2.723178
C	-4.926460	-1.045439	0.596326
H	-5.316226	-0.552874	1.494312
H	-5.307373	-2.077872	0.605209
C	-4.756993	-0.893486	-1.931582
H	-5.160282	-1.897999	-2.123016
H	-5.033708	-0.279201	-2.797824
C	-5.432363	-0.330660	-0.666703
H	-5.159148	0.728200	-0.577468
C	-6.957482	-0.409632	-0.774967
H	-7.440475	0.019834	0.109025
H	-7.320541	0.133373	-1.654139
H	-7.290155	-1.450561	-0.865541
C	-2.509274	0.358149	-1.801146
H	-1.458685	0.238782	-2.090853
H	-2.977455	1.038885	-2.524682
C	-2.858342	0.337226	0.713464
C	-2.511351	0.993162	-0.440228
C	-2.812986	1.068604	1.931076
H	-3.084383	0.536743	2.837456
C	-2.047647	3.084506	0.787720
C	-2.432333	2.383523	1.980521
H	-2.390795	2.939095	2.909511
N	-2.126546	2.304356	-0.367754
H	-1.821896	2.767996	-1.215367
O	-1.652909	4.266311	0.715258
O	0.218282	-0.380988	-0.617495
Fe	1.926211	-0.284530	-0.260492
N	4.034481	-0.153290	0.256985
C	4.811461	-1.210793	0.398973
C	4.765491	0.948308	0.643659
H	4.510979	-2.223406	0.180675
C	6.018574	0.546899	1.021707
H	4.329381	1.933917	0.621740
H	6.877697	1.090152	1.380547
N	1.849542	1.842370	-0.578441

C	2.018216	2.537408	-1.760512
C	1.086557	2.592894	0.206295
C	1.345240	3.728223	-1.671242
H	2.581937	2.108899	-2.572215
H	0.737377	2.297713	1.181594
H	1.239439	4.548419	-2.363349
C	1.717350	-0.791836	2.273302
O	1.901396	-1.730561	1.443349
O	1.666187	0.411719	1.877815
C	1.565870	-1.095358	3.748379
H	0.650687	-0.630746	4.125353
H	1.537673	-2.171732	3.922024
H	2.405372	-0.656409	4.296728
O	2.553544	-2.116379	-1.298610
N	0.772153	3.750184	-0.410617
H	0.016383	4.350709	-0.060087
N	6.029985	-0.829734	0.859842
H	6.804297	-1.445366	1.053773
C	2.792524	-1.470868	-2.367097
O	2.634613	-0.216761	-2.401122
C	3.295265	-2.212321	-3.586333
H	2.699453	-3.114736	-3.744597
H	3.260433	-1.573595	-4.469774
H	4.329721	-2.527618	-3.412135

Fe^{III}-OH-Int1

C	-3.385436	-1.243176	0.625395
C	-1.768189	-2.472089	-0.900603
C	-1.238922	-3.265413	0.248311
C	-1.545092	-2.551949	1.581136
H	-1.356706	-2.626922	-1.896602
H	-1.722613	-4.261316	0.270241
H	-0.163397	-3.439543	0.139255
H	-1.304178	-3.200355	2.429276
H	-0.911133	-1.662780	1.662186
H	-0.142984	-1.125164	-0.959861
N	-2.941039	-2.121355	1.716274
H	-3.548669	-2.937765	1.743045
C	-3.126726	-1.866582	-0.778563
H	-3.858913	-2.694632	-0.861972
C	-3.493602	-0.835299	-1.859286
H	-3.322549	-1.281947	-2.846707
C	-4.906468	-1.007929	0.754392
H	-5.124631	-0.602114	1.748491
H	-5.418257	-1.979407	0.684059
C	-4.996245	-0.512557	-1.740011
H	-5.556999	-1.417895	-2.012272
H	-5.275644	0.255029	-2.472528
C	-5.447486	-0.066544	-0.335060
H	-5.029337	0.929476	-0.144177
C	-6.972422	0.047851	-0.259876
H	-7.295327	0.398780	0.725841
H	-7.353877	0.750216	-1.008611
H	-7.447601	-0.923475	-0.441666
C	-2.581391	0.401654	-1.755117
H	-1.587539	0.182230	-2.165187
H	-3.003211	1.226538	-2.344470
C	-2.677478	0.099747	0.762158
C	-2.367197	0.855727	-0.339914
C	-2.434770	0.665644	2.044075
H	-2.679464	0.057815	2.909450
C	-1.561770	2.722362	1.060408
C	-1.906045	1.918789	2.201164
H	-1.717410	2.348773	3.177285
N	-1.829275	2.100669	-0.160904
H	-1.548691	2.629848	-0.977749
O	-1.058883	3.863061	1.083948
O	0.391020	-0.332291	-1.128369
Fe	2.212293	-0.527667	-0.853093
N	4.354316	-0.362634	-0.199848
C	5.312747	-0.068544	-1.063780
C	4.919448	-0.326733	1.059346
H	5.170034	0.053862	-2.130619
C	6.247930	-0.016172	0.951749
H	4.321398	-0.496542	1.939140
H	7.026684	0.102667	1.687678
N	2.284237	1.595174	-0.788493
C	2.601498	2.532290	-1.752920
C	1.490494	2.188749	0.090796
C	1.974983	3.710577	-1.435089
H	3.226959	2.264468	-2.591903
H	1.041704	1.701592	0.940726
H	1.965766	4.672911	-1.921152
C	1.940796	-1.805860	1.343791
O	2.055441	-2.325601	0.176406
O	1.933948	-0.554933	1.484430
C	1.868546	-2.723169	2.541605
H	1.387946	-2.218574	3.380871
H	1.340589	-3.645040	2.291360
H	2.889317	-2.993679	2.834029
O	2.815084	-1.107438	-2.586429
N	1.284224	3.473865	-0.259012
H	0.562640	4.036674	0.203266
N	6.478804	0.146598	-0.403389
H	7.356577	0.391572	-0.834345
C	3.207358	-0.561208	-3.702236
O	3.927809	0.439595	-3.780361
C	2.715175	-1.282850	-4.944965
H	2.970048	-2.344668	-4.881893
H	1.623217	-1.215745	-4.987877
H	3.148434	-0.842621	-5.843680

TS-C9$_{HT}$

C	-2.949197	-1.066579	0.599038

C	-2.436719	-2.108288	-1.678121
C	-1.396796	-3.017775	-1.016124
C	-0.859808	-2.347881	0.235684
H	-2.893329	-2.599677	-2.546042
H	-1.853365	-3.983327	-0.753847
H	-0.562356	-3.215884	-1.695193
H	-0.090989	-2.915560	0.761159
H	-0.222572	-1.380224	-0.202934
H	-1.923667	-1.211044	-2.040204
N	-1.888548	-1.930806	1.126158
H	-1.518417	-1.599307	2.009851
C	-3.530760	-1.736512	-0.668266
H	-3.998152	-2.672125	-0.332449
C	-4.638207	-0.833821	-1.234143
H	-5.070047	-1.325364	-2.114566
C	-4.065363	-0.978071	1.663653
H	-3.659450	-0.529037	2.580137
H	-4.371616	-2.001111	1.916244
C	-5.758709	-0.676808	-0.188269
H	-6.227662	-1.660697	-0.047867
H	-6.542755	-0.012176	-0.573099
C	-5.278184	-0.167922	1.182944
H	-4.960016	0.875821	1.068682
C	-6.411292	-0.200792	2.211707
H	-6.079151	0.189131	3.179613
H	-7.263786	0.402178	1.881080
H	-6.767915	-1.225628	2.368457
C	-4.051127	0.510971	-1.704959
H	-3.586489	0.386367	-2.692424
H	-4.856511	1.246090	-1.835854
C	-2.469054	0.353419	0.273789
C	-3.016196	1.062428	-0.763313
C	-1.517094	1.023518	1.090361
H	-1.050338	0.474775	1.899092
C	-1.650382	3.057750	-0.250009
C	-1.136450	2.317774	0.871853
H	-0.399519	2.795999	1.503488
N	-2.598527	2.348222	-0.991137
H	-2.972187	2.861079	-1.780816
O	-1.329668	4.205537	-0.609210
O	0.417967	-0.403869	-0.806426
Fe	2.015375	-0.322764	-0.189088
N	3.911939	-0.168848	0.523941
C	4.490001	-1.131708	1.220912
C	4.777008	0.902971	0.495603
H	4.053648	-2.100304	1.405839
C	5.909652	0.576505	1.189122
H	4.509830	1.814612	-0.011422
H	6.811043	1.126995	1.403077
N	2.142047	1.487464	-1.204154
C	1.943101	1.589861	-2.568697
C	1.655795	2.593347	-0.654148

C	1.328173	2.783552	-2.832401
H	2.239300	0.787821	-3.225202
H	1.629413	2.766084	0.410769
H	1.007629	3.238994	-3.755550
C	1.557965	0.609287	2.502019
O	1.392250	-0.408459	1.724427
O	1.979794	1.724321	2.158740
C	1.136106	0.369606	3.949801
H	0.068301	0.598366	4.052620
H	1.285308	-0.673195	4.237811
H	1.685311	1.036119	4.617202
O	2.284629	-2.430320	-0.271041
N	1.170451	3.415198	-1.608534
H	0.497738	4.150795	-1.388878
N	5.709111	-0.718185	1.642208
H	6.356641	-1.264119	2.188761
C	2.750423	-2.405466	-1.463224
O	2.941543	-1.313028	-2.055722
C	3.029817	-3.722639	-2.155299
H	2.079719	-4.143975	-2.501946
H	3.682896	-3.574806	-3.016290
H	3.470129	-4.436940	-1.455617

Fe^{III}-OH-Int1-C9

C	-3.092854	-1.114117	0.542179
C	-2.564196	-2.030638	-1.786272
C	-1.544578	-2.998910	-1.169173
C	-1.069523	-2.477080	0.151359
H	-3.018530	-2.464573	-2.685265
H	-2.018581	-3.991228	-1.051819
H	-0.684383	-3.130801	-1.832267
H	-0.178916	-2.886787	0.613813
H	-0.194773	-0.946758	-0.691755
H	-2.036241	-1.121318	-2.090280
N	-2.045101	-2.015498	1.039647
H	-1.663694	-1.725886	1.932063
C	-3.663973	-1.706113	-0.767516
H	-4.147631	-2.653018	-0.490782
C	-4.750290	-0.756152	-1.296420
H	-5.177841	-1.193421	-2.206856
C	-4.221751	-1.063237	1.594826
H	-3.821232	-0.672662	2.540008
H	-4.549933	-2.092510	1.787097
C	-5.882624	-0.633116	-0.259006
H	-6.370472	-1.614333	-0.176409
H	-6.649430	0.064544	-0.619190
C	-5.412723	-0.205685	1.143100
H	-5.074089	0.836569	1.088824
C	-6.560198	-0.271556	2.154178
H	-6.234316	0.059922	3.145690
H	-7.396730	0.364345	1.845340
H	-6.937740	-1.296261	2.251227

C	-4.132404	0.599835	-1.690129
H	-3.656967	0.517347	-2.676617
H	-4.921586	1.356686	-1.791541
C	-2.579077	0.309800	0.297363
C	-3.098815	1.082240	-0.709945
C	-1.631144	0.922419	1.162780
H	-1.201720	0.337693	1.967740
C	-1.707805	3.022410	-0.075960
C	-1.220752	2.217105	1.011852
H	-0.494047	2.651070	1.685451
N	-2.656432	2.369469	-0.864977
H	-3.008850	2.928199	-1.633215
O	-1.370022	4.185458	-0.365002
O	0.389366	-0.342762	-1.203732
Fe	2.023535	-0.319093	-0.381503
N	4.012091	-0.200787	0.630429
C	4.576543	-1.238340	1.220641
C	4.784238	0.901889	0.921845
H	4.201915	-2.249106	1.175875
C	5.843336	0.519995	1.701081
H	4.513523	1.876801	0.551517
H	6.661106	1.071747	2.135499
N	2.335196	1.699526	-1.016398
C	2.243570	2.031178	-2.356812
C	1.694478	2.648001	-0.339715
C	1.537815	3.197574	-2.474160
H	2.665973	1.387997	-3.112058
H	1.561528	2.623495	0.732068
H	1.249696	3.781592	-3.333344
C	1.460193	0.215437	2.472808
O	1.337299	-0.598180	1.470481
O	1.741853	1.417275	2.401464
C	1.227928	-0.442574	3.829087
H	0.320801	-1.054530	3.811490
H	2.061819	-1.119677	4.043460
H	1.161353	0.309157	4.616513
O	2.526125	-2.384527	-0.636558
N	1.212060	3.582367	-1.183460
H	0.465258	4.226764	-0.911475
N	5.695340	-0.845320	1.882752
H	6.310974	-1.445546	2.408994
C	3.093716	-2.146664	-1.759526
O	3.183167	-0.961700	-2.182104
C	3.658706	-3.301212	-2.552422
H	2.884433	-4.059751	-2.697010
H	4.035276	-2.959385	-3.516839
H	4.470853	-3.767708	-1.985470

Fe^{III}-OH

Fe	0.103886	0.182431	0.855837
O	-0.642691	0.412542	2.508256
N	-1.902396	-0.114038	-0.109462
C	-2.699296	0.826298	-0.730988
C	-2.623285	-1.213756	0.022336
C	-3.927781	0.276898	-0.984541
H	-2.320460	1.817442	-0.920068
H	-2.268891	-2.126162	0.475140
H	-4.818609	0.672723	-1.445184
N	0.902762	-0.082529	-1.198060
C	0.356959	-0.749604	-2.195956
C	2.198993	0.214663	-1.569141
H	-0.646612	-1.142084	-2.208984
C	2.432366	-0.278104	-2.824493
H	2.843036	0.752743	-0.892702
H	3.301014	-0.257030	-3.462497
O	-0.410632	2.290804	0.266965
O	-0.074328	-2.124908	0.758397
O	1.627263	1.766093	0.897625
C	0.790479	2.630670	0.501954
C	1.234157	4.057029	0.270604
H	1.661131	4.134726	-0.735624
H	0.385339	4.739524	0.339089
H	2.009274	4.332608	0.988171
N	1.249426	-0.889051	-3.209798
H	1.080098	-1.359588	-4.085000
N	-3.861550	-1.020038	-0.500564
H	-4.607640	-1.698135	-0.509915
H	-1.275239	1.142918	2.538696
C	1.093532	-2.139060	1.245916
O	1.711572	-1.045875	1.444657
C	1.760543	-3.441590	1.620094
H	1.697735	-3.568410	2.705972
H	1.263692	-4.284044	1.136192
H	2.818942	-3.412301	1.351438

Fe^{II}-OH

Fe	-0.083713	-0.435083	0.791044
O	0.925940	-1.959420	1.416931
N	1.837865	0.185072	-0.404109
C	2.256640	1.093631	-1.348336
C	2.841151	-0.642699	-0.195055
C	3.547849	0.814952	-1.720254
H	1.593586	1.867059	-1.707358
H	2.749798	-1.477308	0.494919
H	4.222195	1.276410	-2.425212
N	-0.772739	1.584400	-0.083967
C	-0.457919	2.747340	0.446052
C	-1.279220	1.828003	-1.346752
H	-0.039035	2.865237	1.434749
C	-1.266375	3.180404	-1.577808
H	-1.633319	1.005655	-1.960411
H	-1.583721	3.776890	-2.419001
O	-0.913376	-1.180229	-0.993091
O	0.029809	0.959992	2.698183

O	-2.807113	-0.859842	-2.157184
C	-2.162364	-1.378569	-1.226631
C	-2.881391	-2.314311	-0.242606
H	-2.317940	-3.247157	-0.127435
H	-2.906192	-1.830505	0.739439
H	-3.899079	-2.532331	-0.577770
N	-0.737888	3.756548	-0.427654
H	-0.598167	4.739810	-0.260912
N	3.908701	-0.297283	-0.972905
H	4.793764	-0.776516	-1.006869
H	0.574282	-2.696492	0.899373
C	-1.119357	0.516791	2.998018
O	-1.782907	-0.270729	2.265805
C	-1.721530	0.937206	4.339635
H	-1.283945	0.319199	5.132180
H	-1.474267	1.980023	4.559429
H	-2.804289	0.793331	4.344205

Int2

C	-0.647670	0.559110	-0.987220
C	-0.802400	1.835890	1.506950
C	-0.944680	3.126450	0.778110
C	-0.245640	2.961750	-0.621870
H	0.171280	1.646740	1.953030
H	-1.994700	3.393250	0.622580
H	-0.382800	3.854350	-1.233640
N	-0.852480	1.824760	-1.313630
H	-1.742380	2.025230	-1.760160
C	-1.687440	0.834640	1.371610
H	-2.660600	1.068090	0.939460
C	-1.465970	-0.620480	1.710250
H	-1.832920	-0.793420	2.730850
C	-1.675760	-0.442540	-1.439770
H	-1.369030	-0.711860	-2.462080
H	-2.657590	0.039450	-1.519440
C	-2.304130	-1.545410	0.799610
H	-3.334920	-1.170330	0.767350
H	-2.357380	-2.531340	1.272890
C	-1.792710	-1.750160	-0.633960
H	-0.795830	-2.204560	-0.593760
C	-2.712370	-2.718210	-1.390460
H	-2.332030	-2.927520	-2.394450
H	-2.790090	-3.668860	-0.857050
H	-3.723210	-2.307980	-1.488420
C	0.021700	-1.020220	1.731370
H	0.488690	-0.601960	2.631750
H	0.080120	-2.108740	1.853550
C	0.681140	0.128050	-0.545440
C	0.942230	-0.638440	0.582500
C	1.791090	0.510140	-1.387660
H	1.577170	1.112370	-2.265660
C	3.377540	-0.713860	0.006440
C	3.064440	0.113750	-1.144070
H	3.887290	0.385980	-1.793050
N	2.229740	-1.006790	0.799920
H	2.456670	-1.544690	1.630590
O	4.463310	-1.143580	0.344580
H	0.821150	2.783320	-0.496680
H	-0.452480	3.962460	1.283350

TS_{retro}

C	-0.857380	0.452650	-0.656090
C	-1.131150	1.918020	1.327660
C	-1.533720	3.130750	0.774580
C	-0.645010	2.842540	-0.904010
H	-0.239490	1.911260	1.950320
H	-2.549390	3.221290	0.396820
H	-0.978820	3.727970	-1.438290
N	-1.091840	1.656590	-1.398020
H	-1.973080	1.679700	-1.900510
C	-1.635600	0.660180	0.821440
H	-2.692730	0.727230	0.551500
C	-1.348670	-0.617520	1.631170
H	-1.864630	-0.524750	2.592030
C	-1.530240	-0.746480	-1.347170
H	-1.049630	-0.885470	-2.321610
H	-2.588570	-0.511810	-1.531100
C	-1.952000	-1.828420	0.898030
H	-3.041680	-1.696430	0.858320
H	-1.775900	-2.730490	1.493080
C	-1.424000	-2.040770	-0.528180
H	-0.361460	-2.306350	-0.471440
C	-2.159860	-3.186570	-1.227710
H	-2.051040	-4.117850	-0.665200
H	-3.230640	-2.972540	-1.316210
H	-1.764600	-3.355510	-2.233560
C	0.157440	-0.751120	1.922320
H	0.431400	-0.150830	2.800460
H	0.375120	-1.789100	2.199600
C	0.615000	0.224680	-0.391230
C	1.051130	-0.356260	0.774390
C	1.577310	0.470160	-1.428640
H	1.226320	0.882120	-2.370470
C	3.407820	-0.352750	-0.017720
C	2.900560	0.210880	-1.259850
H	3.629800	0.403710	-2.036870
N	2.380580	-0.606120	0.929720
H	2.716890	-1.036080	1.784580
O	4.565330	-0.615220	0.256010
H	0.398330	2.858950	-0.608120
H	-1.109840	4.053620	1.162780

Int3

C	-0.845437	0.348989	-0.690413

C	-1.290697	1.990040	1.221306
C	-2.045048	3.090501	1.083437
C	-0.614672	2.673619	-1.442101
H	-0.350411	2.066670	1.761884
H	-3.005838	3.064392	0.574751
H	-1.756313	4.037475	1.528089
H	-1.006453	3.507514	-2.014907
N	0.261121	2.794622	-0.818347
H	-1.125376	1.501125	-1.584211
C	-1.932672	1.393778	-2.195452
H	-1.643568	0.656769	0.640717
C	-2.708175	0.651243	0.381266
H	-1.357951	-0.533082	1.587335
C	-1.921452	-0.362762	2.509469
H	-1.419607	-0.917000	-1.355985
H	-0.895472	-1.100685	-2.300120
C	-2.482608	-0.753535	-1.589318
H	-1.874551	-1.835862	0.950711
H	-2.967055	-1.765172	0.865501
C	-1.673686	-2.674263	1.625961
H	-1.288066	-2.141310	-0.436539
C	-0.217807	-2.353518	-0.325975
H	-1.950039	-3.369399	-1.066026
H	-1.513781	-3.599371	-2.042538
H	-1.821228	-4.247706	-0.427678
C	-3.025366	-3.211361	-1.203424
H	0.138276	-0.573479	1.955518
H	0.344000	0.112001	2.787479
C	0.393941	-1.573494	2.325248
C	0.634524	0.228739	-0.411104
C	1.059247	-0.229353	0.813789
H	1.615595	0.413678	-1.442505
C	1.287314	0.706566	-2.436446
C	3.435286	-0.191704	0.087270
H	2.943084	0.229070	-1.216437
N	3.686090	0.370193	-1.991413
H	2.390587	-0.410251	1.024380
O	2.716544	-0.750739	1.922681
H	4.594322	-0.373659	0.413534

Int1

C	-0.901350	0.461850	-0.608810
C	-1.270570	1.908870	1.413180
C	-1.345280	3.139970	0.576490
C	-0.663690	2.904330	-0.792060
H	-1.141300	1.969380	2.489930
H	-2.402480	3.407080	0.386420
H	-0.878490	3.732380	-1.475090
N	-1.062980	1.657600	-1.448450
H	-2.037120	1.719120	-1.737280
C	-1.626470	0.614620	0.765830
H	-2.707120	0.624340	0.524140
C	-1.351710	-0.634480	1.616220
H	-1.888000	-0.540080	2.568700
C	-1.515460	-0.745160	-1.347920
H	-1.037120	-0.845740	-2.328220
H	-2.581120	-0.539020	-1.529850
C	-1.899370	-1.876280	0.888170
H	-2.994610	-1.790340	0.854860
H	-1.678570	-2.779840	1.470080
C	-1.381510	-2.051740	-0.551230
H	-0.313750	-2.298690	-0.502150
C	-2.096090	-3.207660	-1.255350
H	-1.707490	-3.354660	-2.268240
H	-1.967060	-4.146200	-0.706110
H	-3.172200	-3.013120	-1.334660
C	0.150790	-0.722660	1.942910
H	0.386730	-0.051810	2.779770
H	0.401660	-1.736140	2.283260
C	0.583390	0.214370	-0.379010
C	1.035380	-0.353390	0.783020
C	1.542270	0.484190	-1.403470
H	1.171870	0.923090	-2.324620
C	3.385940	-0.351470	-0.017930
C	2.871830	0.225230	-1.244170
H	3.597290	0.436490	-2.020570
N	2.373130	-0.604610	0.929270
H	2.712850	-1.023200	1.786690
O	4.553990	-0.625680	0.245970
H	0.420970	2.875790	-0.648010
H	-0.896520	4.002200	1.082270

TS_{HCC}

C	-0.889840	0.452500	-0.608510
C	-1.272220	1.878090	1.453840
C	-1.746730	3.058220	0.981100
C	-0.693620	2.896830	-1.116060
H	-0.416270	1.883090	2.123500
H	-2.662270	3.096490	0.397700
H	-1.037380	3.682820	-1.783260
N	-1.108380	1.610480	-1.487100
H	-2.046870	1.595820	-1.870800
C	-1.631900	0.593080	0.770070
H	-2.704550	0.609990	0.530050
C	-1.340970	-0.671180	1.599950
H	-1.872190	-0.586010	2.554970
C	-1.468440	-0.778140	-1.347340
H	-0.978680	-0.866700	-2.323500
H	-2.537030	-0.595050	-1.538930
C	-1.874670	-1.914490	0.868280
H	-2.969210	-1.833030	0.811310
H	-1.661710	-2.815990	1.456410
C	-1.321300	-2.083240	-0.555610
H	-0.249830	-2.308700	-0.483980

C	-1.996110	-3.250330	-1.280170
H	-1.581780	-3.386500	-2.284370
H	-1.860480	-4.187500	-0.730430
H	-3.073870	-3.077340	-1.383000
C	0.164910	-0.762100	1.919160
H	0.401000	-0.138050	2.791220
H	0.421700	-1.790010	2.207800
C	0.599190	0.251940	-0.373760
C	1.051230	-0.334950	0.779560
C	1.563790	0.564040	-1.381410
H	1.199950	1.006790	-2.302970
C	3.410740	-0.258050	0.007630
C	2.896910	0.332870	-1.211950
H	3.625270	0.576730	-1.975860
N	2.392830	-0.557330	0.935720
H	2.731890	-0.989090	1.786940
O	4.581540	-0.510160	0.279190
H	0.331180	2.973610	-0.770740
H	-1.408230	4.007380	1.385510

Int1'

C	-0.823730	0.323390	-0.821660
C	-1.486850	2.035910	0.993780
C	-2.480380	2.807790	1.437180
C	-0.605420	2.621110	-1.752350
H	-0.459480	2.375240	1.100110
H	-3.520570	2.502760	1.348940
H	-0.958560	3.309600	-2.510580
N	-1.002040	1.293350	-1.906160
H	-1.912780	1.194280	-2.339130
C	-1.691930	0.656210	0.430500
H	-2.743500	0.566710	0.127490
C	-1.418960	-0.430080	1.500760
H	-2.049740	-0.210960	2.368850
C	-1.277850	-1.050200	-1.375270
H	-0.699960	-1.269720	-2.279370
H	-2.332660	-0.969540	-1.679840
C	-1.815130	-1.815320	0.963770
H	-2.903660	-1.820420	0.811760
H	-1.604930	-2.582200	1.719900
C	-1.135720	-2.191380	-0.361460
H	-0.064890	-2.335690	-0.171580
C	-1.691820	-3.503330	-0.919910
H	-1.188740	-3.782460	-1.851340
H	-1.558530	-4.323550	-0.206930
H	-2.764120	-3.416360	-1.131240
C	0.046580	-0.345910	1.965580
H	0.152520	0.469500	2.693500
H	0.326590	-1.267080	2.492990
C	0.646500	0.242180	-0.434040
C	1.016580	-0.103890	0.839920
C	1.682980	0.399180	-1.406860
H	1.386700	0.648040	-2.420580
C	3.428670	-0.078810	0.249260
C	3.002500	0.254960	-1.095340
H	3.785080	0.380370	-1.833770
N	2.345490	-0.240510	1.137330
H	2.623450	-0.493630	2.077860
O	4.578700	-0.230470	0.651520
H	0.371630	2.790520	-1.317420
H	-2.293660	3.777640	1.886910

Int$_{reb}$

C	0.711410	0.007230	-0.881920
C	1.685590	-1.651420	0.792110
C	2.085620	-2.541940	-0.384020
C	1.131940	-2.318640	-1.567060
H	2.075990	-3.598040	-0.081920
H	0.127410	-2.664470	-1.295610
H	0.687650	-1.964760	1.136150
N	1.029260	-0.917820	-1.981610
H	1.910530	-0.625420	-2.397780
C	1.633300	-0.188480	0.350650
H	2.654530	0.077150	0.043280
C	1.204140	0.784420	1.461330
H	1.872810	0.648480	2.315650
C	0.907850	1.446880	-1.407900
H	0.278830	1.588130	-2.293600
H	1.953420	1.555540	-1.734210
C	1.356880	2.228890	0.952680
H	2.426560	2.417470	0.784990
H	1.036890	2.935170	1.729110
C	0.598550	2.518740	-0.353840
H	-0.477410	2.475210	-0.143130
C	0.914420	3.920930	-0.879550
H	0.353630	4.137130	-1.794710
H	0.659230	4.686890	-0.139770
H	1.981780	4.022020	-1.108980
C	-0.225000	0.455960	1.933780
H	-0.191120	-0.369090	2.658300
H	-0.647670	1.314430	2.472360
C	-0.744930	-0.183760	-0.470670
C	-1.153030	0.064630	0.814470
C	-1.743430	-0.539450	-1.428570
H	-1.409220	-0.723660	-2.444710
C	-3.522870	-0.426470	0.256780
C	-3.060680	-0.662090	-1.096620
H	-3.814930	-0.937920	-1.823790
N	-2.478570	-0.064300	1.131900
H	-2.785470	0.117690	2.079850
O	-4.674220	-0.507600	0.677300
H	1.445140	-2.909110	-2.433400
H	3.115120	-2.294900	-0.674030
O	2.618090	-1.724820	1.870970

H	2.662020	-2.646210	2.157920

Fe^{II}

Fe	-0.543513	0.265086	0.432369
N	1.616330	0.353523	0.101537
C	2.375499	0.823421	-0.950602
C	2.438582	-0.295720	0.908086
C	3.682723	0.457442	-0.770713
H	1.926082	1.369626	-1.765614
H	2.128187	-0.796954	1.813743
H	4.571423	0.621762	-1.358467
N	-1.091887	2.026805	-0.723938
C	-0.422385	3.154719	-0.874981
C	-2.349727	2.230079	-1.256694
H	0.588566	3.335961	-0.543012
C	-2.431855	3.506441	-1.744889
H	-3.085023	1.441365	-1.215377
H	-3.228599	4.048793	-2.227719
O	-0.807798	-1.302949	-0.878515
O	-0.021997	-0.846434	2.461050
O	-2.690975	-0.556417	0.012133
C	-2.081440	-1.369527	-0.730742
C	-2.826272	-2.460911	-1.469285
H	-2.554821	-2.444877	-2.528684
H	-2.520380	-3.433621	-1.071233
H	-3.904065	-2.342741	-1.352120
N	-1.197195	4.081885	-1.494904
H	-0.926764	5.028099	-1.714134
N	3.703501	-0.251561	0.417652
H	4.512261	-0.681030	0.839803
C	-0.405803	0.238090	2.974112
O	-0.752400	1.232819	2.242927
C	-0.491802	0.398250	4.477476
